# Supplementary material for: Molecular phylogenies map to biogeography better than morphological ones
Source: Commun Biol. 2022 May 31;5:521. doi: 10.1038/s42003-022-03482-x (PMC9156683; doi:10.1038/s42003-022-03482-x)
Supplement: Supplementary file 2 — Supplementary Information [file 42003_2022_3482_MOESM2_ESM.pdf]

Supplementary Information:

Molecular phylogenies map to biogeography better than morphological ones

Jack Oyston[1]*(jwo22@bath.ac.uk), Mark Wilkinson[2], Marcello Ruta[3] &

Matthew A. Wills[1]*(bssmaw@bath.ac.uk)

[1] Milner Centre for Evolution, Department of Biology & Biochemistry, University of Bath, Bath, UK

[2] Vertebrates Division, Department of Life Sciences, Natural History Museum, Cromwell Road, London, UK

[3] School of Life Sciences, Joseph Banks Laboratories, College of Science, University of Lincoln, Lincoln, UK

Supplementary Methods

Sample Collection & Phylogeny Treatment

We sought data for published phylogenies of as wide a range of plant and animal clades as possible, partitioned into two categories: morphological and molecular. Searches were conducted from 1981 to 2016 using Google Scholar and Web of Science, and using clade names (Classes, Orders and Families as well as levels between those ranks), plus *phylog*, *distribution* and *geog* as search terms. We limited our search to clades in which constituent groups (sub-clades or operational taxonomic units) had heterogeneous but unambiguously demarcated ranges and distributions (marine clades and migratory clades were largely omitted for this reason). As far as possible, we sought source papers that presented both morphological and molecular trees, as these tended to have identical taxon sets and sampling procedures for each phylogeny, making them directly comparable. These searches resulted in a sample of 106 published trees from 61 papers, which was reduced to a sample of 96 trees with a morphological and molecular phylogeny selected for 48 clades. Datasets were classed as morphological if they did not include any DNA, RNA or protein data, regardless of whether such characters were soft part, hard part or physiological. Similarly, datasets were classed as molecular if they contained only DNA, RNA or protein data. No distinction was made between different sources of molecular data, although most molecular datasets consisted of DNA sequence data incorporating multiple genes. From a total of 48 trees inferred from morphology, 45 were constructed using cladistic matrices under maximum parsimony, 2 were a consensus of previous phylogenetic studies and 1 was a UPGMA dendrogram. From a total of 48 trees inferred from molecular data, 30 were constructed using maximum parsimony, 12 using maximum likelihood, 5 using Bayesian inference and 1 as a consensus of previous phylogenetic studies. Phylogenies were preferentially taken from the main text of the paper, with supplementary material only being used if there were no suitable trees figured in the main text. In some cases, papers presented a combined analysis of all data, with separate morphological and molecular trees as supplementary materials. In other cases, multiple

morphological or molecular phylogenies were presented in the main paper or as supplementary information, in which case we used that preferred by the authors (either on grounds of analytical rigor or inclusivity of data). In cases where no preference was expressed, the most inclusive (in terms of taxa, then in terms of characters) were used. Finally, if all possible candidate trees contained the same number of taxa and characters, the most resolved topologies were used. In order to control for the size and nature of the taxon sample, the minimum number of leaves were pruned from one or both trees to make the leaf sets identical. The percentage of taxa removed from source matrices was relatively low, (11% for morphological datasets and 22% for molecular data) and most clades had their full original taxon set (70% of morphological datasets, 66% of molecular). The dataset comprised mostly clades of animals (85%, with 73% vertebrates and 13% insects), with the remainder (15%) being plants. Mammals had the greatest representation (46%), followed by reptiles (19%), birds (6%) and amphibians (2%). Constituent taxa were predominantly terrestrial (85%) and freshwater, with strong patters of endemism. Only 10% of clades contained one or more marine taxa.

Characterising Biogeographic Regions

Biogeographic data were obtained from The IUCN Red List of Threatened Species, Version 2019-2 [1] (http://www.iucnredlist.org), the Global Biodiversity Information Facility, accessed 29th Dec 2019 [2] (https://www.gbif.org) and The Reptile Database, accessed 24th Dec 2019 [3] (http://www.reptile-database.org). These data were checked and augmented using any biogeographic data from the source publications. In some cases, distributional data were available as recorded point occurrences, but more frequently biogeographic data took the form of a list of geographic areas in which the taxon was present.

As the sampling of point occurrences was extremely uneven (both spatially and across taxa in the same clade) these were grouped and summarised as a list of areas in which the taxon was found, using the smallest discrete areas given for *other* taxa in the clade. This ensured that biogeographic resolution was consistent across the group and appropriate for the clade

in question. This initial range map of regions at the highest possible resolution for the clade was then used to produce a data matrix of taxon presence/absence within each region (Supplementary Figure 16).

Operational taxonomic units (hereafter "taxa") were scored "1" if present in and "0" if absent from the smallest regions listed. If the regions listed were at different scales for different taxa (e.g., districts for some, countries for others), the larger region was broken up into its constituent sub-regions to match the finest scale given, with taxa coded as present in the larger region also coded as present in all the constituent sub-regions. For example, if one taxon was listed as occurring in 'North America' but several other taxa had distributions listed as being limited to specific states, the 'North American' taxon would be recorded as occurring in all the states harbouring the other taxa. This helped to ensure that all regions for a clade were summaries of biogeographic distributions at the same scale. Regions were then checked to ensure none of them overlapped or were duplicates of the same geographical area to produce a full list of the least inclusive regions in which the members of the clade were found.

The list for each taxon was converted into a single matrix for the clade, within which presence in a region was encoded by 1s and absences by 0s. Several steps were taken to reduce such matrices down and eliminate redundant or duplicate information. Firstly, regions with identical taxon codings (i.e., the same taxon set was present in both regions) were combined, defining a new set of regions with unique presence/absence codings. By this process, adjacent regions with identical codings were combined into a single larger region until all regions were adjacent to regions with different taxon compliments. Although duplicate regions or regions with the same presence/absence codings strengthen the associations between certain taxa in the matrix they are not biogeographically distinct (unlike regions with unique taxon compositions) and so were amalgamated. Conversely, automorphic characters serve no role in determining the fit of region characters onto phylogenies. As our region characters serve only to identify groupings of taxa with overlapping or proximal distributions, such autapomorphic region characters could serve no purpose. The second step therefore involved removing all regions

containing only a single taxon. In cases where this would result in a taxon being removed from the matrix, the “autapomorphic” region was instead combined with the geographically most proximate region and the region containing fewest taxa in the case of ties. This ensured that all automorphic characters were removed from the matrix while still retaining distributional data on all taxa in the clade. Finally, the list of biogeographic regions was checked to ensure that they were broadly comparable in terms of biogeographic separation (for example continents or island archipelagos, neighbouring islands in a chain). For clades with a global distribution, this approach typically resulted in biogeographic regions broadly congruent with the modified version of Wallace’s biogeographic regions from Holt *et al.* (2013) [4].

Dataset Properties

Our source trees differed considerably in their size (number of taxa), balance, taxonomic scope, biogeographic range and the number of regions distinguished, as well as whether they were fully resolved trees or contained polytomies (Supplementary Table 5).

All of these might be expected to potentially influence or bias statistics intended to represent the goodness of fit of the biogeographic data to the tree. However, all of these properties except tree balance and resolution (number of resolved nodes and whether they contained polytomies) were controlled in our sample of morphological and molecular tree pairs. We nevertheless summarise these variables (Supplementary Table 3).

Colless’s index [5] of tree imbalance (*C*) for a given tree (T) was calculated using the GHOSTS 2.4 script [6]. This index tallies the number of terminal taxa subtended by the right hand (T_R) and left hand (T_L) branches at each internal node, then scales this value by a function of the number of taxa in the tree (n):

$$C(T) = \frac{\sum T_R - T_L}{(n-1)(n-2)/2} \quad (1)$$

Index values of 0 reflect a perfectly balanced tree, with values increasing as the topology becomes more imbalanced up to a value of 1 for a completely pectinate tree.

Measures of Biogeographic Fit

Biogeographic congruence for each clade was evaluated by parsimoniously optimising the corresponding biogeographic matrix onto both morphological and molecular trees using PAUP* 4.0 [7]. Both the ensemble consistency index (CI) and the retention index (RI) were then compared (Supplementary Figure 5). In addition to comparing the distributions of values for morphological and molecular trees, the differences in values in each clade pair were compared to the pseudomedian calculated from both distributions. The pseudomedian is a non-parametric measure of centrality in populations [8] and is the median of the distribution of:

$$(Z_1 + Z_2)/2 \quad (2)$$

Where both Z_1 and Z_2 have the same distribution F. If F is symmetric then the pseudomedian will coincide with the median. Differences were calculated as the value for the molecular tree minus the morphological tree, so a skew of positive values relative to the pseudomedian indicates better fit (higher values) for molecular trees while values evenly distributed around the pseudomedian indicate no general difference in fit.

Consistency index

The CI [9] is given by the minimum possible number of state changes (the total number of states in the matrix, minus the number of characters) divided by the number of observed state changes on the tree. A 1:1 correspondence of phylogeny and biogeography (i.e., all regions correspond to monophyletic clades) results in a CI of 1.0.

$$CI = \frac{MinSteps}{ObsSteps} \quad (3)$$

It is well recorded [10] that the CI is negatively correlated with the number of taxa in the dataset and to a lesser degree the number of characters [11]. This means that CI values are only really comparable for trees derived from the same data (since only *ObsSteps* varies and *MinSteps* is invariant for the same character matrix). However, in our study both the taxon set and the region characters were identical for the morphological and molecular trees being compared,

with only the tree topologies differing. Therefore, neither of these factors should bias comparisons made in this study.

Retention index

The retention index (RI) [12] is an index of retained synapomorphy (shared, derived states), and is less sensitive to the numbers of taxa and characters in the matrix [13]. The RI is the maximum number of possible steps minus the observed number of steps, divided by the maximum number of possible steps minus the minimum number of steps. An RI of 1 means the character set fits onto the tree perfectly, an RI of 0 means the character set fits the tree as poorly as possible.

$$RI = \frac{MaxSteps - ObsSteps}{MaxSteps - MinSteps} \tag{4}$$

The RI is still sensitive to the number of states per character, with values becoming increasingly inflated as the number of character states increases (and the number of taxa with any given state decreases) [14]. States shared by fewer taxa have fewer homoplastic configurations, resulting in increasingly inflated RI values with more unique character states.

Biogeographic homoplasy excess ratio

The homoplasy excess ratio (HER) [11,13,15,16] was designed to overcome these biases in CI (and to a much lesser extent the RI) caused by the differences in the dimensions of datasets, particularly the anticipated increase in homoplasy with increasing numbers of taxa. The HER is given by the observed homoplasy excess (the number of steps observed on the minimum-length tree minus the minimum possible number of steps) divided by the expected homoplasy excess (the mean number of steps for minimum-length trees for randomly permuted biogeographic data, minus the minimum possible number). In the original implementation of the index, data are randomised (to infer the mean number of steps for minimum-length trees for randomly permuted biogeographic data) by reassigning states across taxa but within each character. This breaks down character correlations and the inter-nested structure necessary to infer phylogeny. Hence:

$$HER = 1.0 - \frac{ObsSteps - MinSteps}{MeanSteps - MinSteps} \quad (5)$$

The ratio of the observed homoplasy excess/expected homoplasy excess is subtracted from 1.0 so that the HER will be 1.0 when no homoplasy is present. Completely phylogenetically random data has an expected HER of 0.0.

The biogeographic HER (bHER) modified this procedure in two ways. Firstly, we treated the biogeographic data as a single column, randomly reassigning these to rows (i.e., across species). This means that no new biogeographic distribution patterns were generated, but rather the existing patterns were randomly reassigned to species. Secondly, for the original HER, each randomised matrix is reanalysed to yield most parsimonious trees. For our new index, we optimised the biogeographic characters onto the *original* tree (effectively randomising the assignment of species and their biogeographic distributions across the same topological branching structure). We did this 10,000 times to calculate a mean (*MeanSteps*). *ObsSteps* was then derived from the most parsimonious optimisation, while *MinSteps* was equal to the number of region characters (more precisely the total number of states in the matrix, minus the number of characters, but all characters had 2 states).

Significance values for CI and RI

We used randomisation tests to determine whether the observed values of CI and RI differed from those expected for the given topology and dataset. As outlined above, the HER already scales its measure of homoplasy relative to the amount of homoplasy expected given the data and tree, while the CI and RI do not. Therefore, we randomly reassigned the block of region character codings for each taxon across the terminals 10,000 times, to produce 10,000 randomly permuted region matrices (the same underlying procedure as for the bHER above). CI and RI were then calculated for each randomisation to produce distributions of expected CI and RI values. Observed CI and RI values were then compared to these distributions (rather than its mean, as in the case of the bHER). Observed values that fell beyond the 95th percentile were taken to show biogeographic congruence that deviated significantly from the null expectation.

Testing for Dataset Biases

Before further statistical analyses, we performed Shapiro-Wilks tests for normality on the distributions of the phylogenetic characters used in the source trees, the number of taxa, the number of biogeographic regions, publication year, Colless's index of tree balance, the proportion of resolved nodes, CI, RI, CI & RI p-value and bHER (Supplementary Table 13).

Because most data partitions (morphological trees only, molecular trees only or all trees) were non-normally distributed we subsequently employed non-parametric statistical tests. This consisted of stepwise linear regression selecting the combinations of variables with lowest AIC (Supplementary Table 4), as well as subsequent paired two-tailed Wilcoxon signed-rank tests of model residuals for both tree types. To test whether variables other than data type (morphological or molecular) affected our indices of biogeographic fit, we produced several nested linear models with each fit metric as the dependent variable (Table 2). Model fit was evaluated using the Akaike information criterion (AIC). AIC values indicated that the best-supported models included data type, but also the age of the root node, the proportion of resolved nodes, number of region characters, number of taxa, publication year and the ratio of the number of phylogenetic characters underlying the trees to the number of taxa as additional variables, depending on the fit metric used.

Residuals from these models for CI, RI and bHER (and from minimum adequate models selected by the AIC) all demonstrated better biogeographic congruence for molecular trees, and these differences being significant for the residual CI and residual bHER (Table 2). The CI had the highest correlation with these variables and the minimum adequate model (MAM) chosen by the AIC retained the number of taxa and number of geographical regions. The RI was more weakly correlated and the MAM retained the proportion of resolved nodes and the number of biogeographic regions. The bHER showed the weakest correlation overall as well as showing a weak correlation with the best explanatory variables according to AIC, namely the proportion of resolved nodes and tree balance. Adding tree type (morphological or molecular) to the bHER model resulted in a model with regions, publication year and the

proportion of resolved nodes being favoured by AIC, although correlations were still weak (R^2 = 0.1419, p = 0.00271). We cannot therefore rule out the number of taxa, number of geographic regions, publication year and the proportion of resolved nodes influencing the observed patterns. We note that the number of taxa and the number of geographical regions is the same for a given pair of morphological and molecular trees, whereas tree resolution, balance and publication year often differ. The bHER was designed specifically to mitigate against the effects of differences in tree balance as a potentially confounding factor.

Greater numbers of characters and later publication date might both be expected to correlate positively with phylogenetic accuracy and therefore (by implication) the biogeographic congruence of those phylogenies. To investigate this, we plotted each metric against and the year of publication of those papers (Supplementary Figure 15), the number of phylogenetic characters used to construct the phylogenies in the source papers (Supplementary Figure 17), the number of terminal taxa (Supplementary Figure 18) and the ratio of region characters to taxa (Supplementary Figure 19).

Because the data are non-normal (Supplementary Table 13) and there is no reason to suppose that the relationships are linear (Supplementary Table 14), the Spearman-rank correlation was the preferred method of quantifying the correlation between variables for the whole dataset for the publication year (Supplementary Table 10), number of phylogenetic characters (Supplementary Table 15), number of terminal taxa (Supplementary Table 16) and the ratio of region characters to taxa (Supplementary Table 17). Additionally, a small number of data sets had markedly more characters than the others. While Spearman-rank correlation is robust to outliers (being a correlation of ordered ranks), Pearson's correlations are not, even when data approximate a normal distribution and are not highly heteroskedastic. As Pearson's correlations may provide an informative (though incomplete) description of the linear relationships between variables even when the distributions of variables are non-normal, separate analysis using Pearson's correlation coefficient was used to assess correlations on a subset of the data with outlying large matrices (more than 9,000 characters) removed.

The number of phylogenetic characters underpinning trees was found to correlate positively with bHER (r_s = 0.263, p-value = 0.010) and negatively with CI (r_s = -0.212, p-value = 0.038), but did not correlate with the RI (r_s = 0.144, p = 0.1603). P-values from the randomisation tests are also negatively correlated with the number of phylogenetic characters (r_s = -0.232, p = 0.023). Across our morphological data, bHER and p-values from randomisation tests were significantly correlated with the number of phylogenetic characters in the matrices (bHER, r_s = 0.321, p-value = 0.026; CI & RI p-values, r_s = -0.325, p-value = 0.024), but not with the ratio of phylogenetic characters to taxa (bHER, r_s = 0.227, p-value = 0.130; CI & RI p-values, r_s = -0.041 p-value = 0.778). Molecular trees show neither a significant correlation between these metrics and either the number of phylogenetic characters (bHER, r_s = 0.159, p-value = 0.280; CI & RI p-values, r_s = -0.208, p-value = 0.156) or the ratio of phylogenetic characters to taxa (bHER, r_s = 0.166 p-value = 0.259; CI & RI p-values, r_s = -0.042, p-value = 0.779). There is no clear reason why the CI of biogeographic region characters should be lower for trees underpinned by more phylogenetic characters, in fact, one might expect phylogenetic accuracy to improve for larger datasets, resulting in better biogeographic fit. The fact that CI decreases significantly with increasing numbers of terminal taxa (r_s = -0.747, p-value = $2.2x10^{16}$), strongly suggests that the decrease in CI is driven by an increase in tree size, rather than the size of the matrix itself. When outliers are removed, the number of phylogenetic characters in source datasets shows a significant positive correlation with the number of terminal taxa (r_s = 0.260, p-value = 0.013, n = 90). We found no evidence that any of our fit metrics showed a significant correlation between CI and either the ratio of phylogenetic characters to terminal taxa (SupplementaryTable 18 or the ratio of region characters to terminal taxa (Supplementary Table 19). The bHER showed a weak positive correlation with the ratio of phylogenetic characters to terminal taxa for the whole dataset (r_s = 0.240, p = 0.020), suggesting that increasing numbers of characters underpinning relationships relative to the number of taxa in the tree improves biogeographic congruence.

Compatibility Tests of Biogeographic Region Matrix Structure

Intuitively, differences in biogeographic congruence are most impressive when the structure of the biogeographic binary presence/absence matrices are consistent with its containing phylogenetic signal. This was assessed using tree independent matrix compatibility permutation tail probability (MCPTP) tests, using the MATRIX program in PICA 4.0 [17]. Firstly, the total number of observed pairwise incompatibilities for the matrix of region characters, the incompatibility score, was calculated. Two characters are incompatible if there is no tree on which they are both free of homoplasy. The observed pairwise incompatibility score was then compared to the distribution of pairwise incompatibility scores from 999 matrices in which the assignments of taxa to states are randomly permuted within characters, preserving the relative numbers of 1s and 0s but reducing any compatibility between characters to that expected by chance alone. This formed the basis of the permutation tail probability (PTP) test, namely whether the observed pairwise incompatibility score was lower than the number of inconsistencies for 95% of the randomly permuted region matrices (Supplementary Table 2). In addition, two descriptive indices based on character compatibility were calculated, both analogous to the HER. The incompatibility excess ratio (1) (IER_1) was the mean incompatibility count for the randomly permuted region matrices minus the original observed incompatibility count, all divided by the mean count for the randomised matrices:

$$\mathrm{IER}_1 = \frac{Mean\ Random\ Count - Observed\ Count}{Mean\ Random\ Count} \quad (6)$$

The second index, the incompatibility excess ratio (2) (IER_2), replaced the mean incompatibility count for randomised matrices with the 95% cut-off point in the distribution of randomised incompatibility counts:

$$\mathrm{IER}_2 = \frac{95\%\ Cutoff\ Point - Observed\ Count}{95\%\ Cutoff\ Point} \quad (7)$$

Comparing Biogeographic Congruence in Morphological and Molecular Trees

The biogeographic congruences of morphological and molecular trees were compared in several different ways. All statistical analyses were implemented in R [18]. Firstly, we tested the number of clades for which each index of biogeographic congruence favoured morphological versus molecular trees against a null of 1:1 (a sign test: Supplementary Table 1).

As the full dataset does not take into account the fact that some topologies may have fit values that are indistinguishable from a random mapping of region characters on the trees (e.g., Supplementary Figure 1), we then repeated the analysis on only those datasets where at least one of the trees showed CI & RI p-values significantly different from the random distributions we generated (p-value < 0.05 for each test, Supplementary Figures 2, 3 & 4).

Secondly, we also performed paired two-tailed Wilcoxon signed-rank tests on all of our measures of biogeographic fit as well as publication year, number of phylogenetic characters in the source papers, the ratio of phylogenetic characters to terminal taxa, the proportion of resolved nodes and Colless's index of tree balance (Table 1).

We find evidence for tree type influencing all three biogeographic congruence metrics, with the bHER showing the biggest difference. Paired Wilcoxon signed-rank tests of model residuals (Table 2) show a significant difference in favour of molecular trees for CI and bHER but not for RI. We therefore think it unlikely that the significant differences in CI and bHER between morphological and molecular trees are driven by other properties of the trees themselves. As RI was found to correlate significantly with the proportion of resolved nodes (r_s = 0.306, p-value = 0.002), and molecular trees were found to have a significantly greater proportion of resolved nodes than their morphological counterparts (Wilcoxon signed-rank: W = 534, Z = 2.75, rc = 0.519, p = 0.006035), we cannot rule out the possibility that differences in RI are driven in part by differences in tree resolution. We might also expect the accuracy of trees to correlate with the volume of data underpinning them. Morphological and molecular datasets contain markedly different numbers of phylogenetic characters overall (Wilcoxon signed-rank: W = 1174, Z = 6.01, rc = 0.997, p = 2.132×10^{-14}), but these two character types

are not directly comparable in terms of information content [24–26]. We also note that morphological and molecular trees conform to the same distribution of Colless's index of tree balance (Wilcoxon signed-rank W = 508.5, Z = -0.815, rc = -0.135, p = 0.4178).

Collecting Stratigraphic Data

In addition to analysing biogeographic congruence, we also assessed the consistency of the phylogenies with stratigraphy. Using the Fossilworks Portal of the Paleobiology Database [19] and The Fossil Record 2 [20], clades that had published fossil material for at least 50% of the terminal clades in the phylogenies were selected from the dataset used in the biogeographic analysis. In total, pairs of morphological and molecular phylogenies for 23 clades (Supplementary Table 6: 18 mammal, 3 reptile, 1 bird & 1 plant) were analysed. For each taxon in the clade, fossil dates were used to assign first occurrences (and last, although these were not used in calculating indices) at the stage-level for all taxa with available material, using the International Chronostratigraphic Chart [21], the Geologic Timescale 2004 [22] and the GeoWhen database [23]. Dates were only used for taxa that could be unambiguously assigned to terminal taxon groups. Taxa can only appear in the fossil record after they evolve, with low preservation potential in many cases ensuring the appearance of fossils in the record lags behind their time of origin. The 'Signor-Lipps effect' also means that taxa are likely to disappear from the fossil record prior to their real extinction as they become scarcer. In consideration of these phenomena, in cases where stratigraphy was unresolved at the stage level, taxa were assigned to the first stage in the time interval given for their first occurrence and the last stage of the time interval for their last occurrence.

Measures of Stratigraphic Fit

Phylogenies were time-calibrated using Strap [24] in R and other indices of stratigraphic fit calculated using the GHOSTS 2.4 program [25]. A brief summary of each metric follows.

The relative completeness index, or SCI [26], measures the proportion of internal nodes in a tree that are stratigraphically consistent. A node is stratigraphically consistent if its sister node is as old or older than it is. Specifically:

$$SCI = \frac{C}{N'} \tag{8}$$

Where C is the number of stratigraphically consistent nodes and N' is the total number of internal nodes in the tree. The SCI is known to be biased by tree balance. In perfectly balanced trees in which each taxon first appears at a different time interval, the SCI cannot fall below 0.5 [6,27].

Another index, the modified Manhattan stratigraphic measure [28] was proposed to deal with the problems inherent to the SCI. It uses the optimization of a Sankoff, Camin-Sokal parsimony character coded from the first occurrence ages of taxa. The cost of each character transition is defined by an asymmetrical step matrix based on the difference in first occurrences between pairs of taxa. These transformation costs penalise transitions between taxa with larger stratigraphic gaps and prohibit reversals with an infinite transition cost. The length of the character optimized onto the tree (L_o) is then compared to the minimum possible length (L_m) in a manner analogous to the CI.

$$MSM^{*} = \frac{L_m}{L_o} \tag{9}$$

The MSM* is also theoretically affected by tree balance, with pectinate trees having higher theoretical maxima than their non-pectinate equivalents. In practice simulations have shown that the MSM* is relatively insensitive to differences in tree balance but tends towards slightly higher median values if first occurrence dates are concentrated towards the base of the tree (bottom-heavy) [6].

The GER [29] is calculated as the difference between the MIG and the minimum possible ghost range for any tree (G_{min}), itself given as a proportion of the range of possible values for the stratigraphic data on any tree. Specifically:

$$GER = 1 - \frac{MIG - G_{min}}{G_{max} - G_{min}} \tag{10}$$

Where MIG is the total minimum implied gap, G_{min} is the minimum possible ghost range and G_{max} is the maximum possible ghost range. The GER is still somewhat sensitive to tree shape

as most non-pectinate trees cannot have MIG values that reach G_{min} or G_{max} and therefore show less extreme maximum values than fully pectinate trees.

The topological GER (GERt) is the GER calculated for a specific topology rather than any topology [19]:

$$GERt = 1 - \frac{MIG - Gt_{min}}{Gt_{max} - Gt_{min}} \quad (11)$$

Where MIG is the minimum implied gap, and Gt_{min} and Gt_{max} are the minimum and maximum possible ghost range on a specified topology. In practice, Gt_{min} and Gt_{max} are estimated by randomization, and the long tails and skewed distribution of possible ghost ranges make it probable that the true bounds will not be found. Moreover, because there are many more ways to achieve a poor stratigraphic fit than a good one, it is likely that Gt_{min} will be overestimated relative to Gt_{max} to yield overestimates of GERt.

Our preferred index of stratigraphic congruence is the modified GER (GER*). This estimates the distribution of MIG values by randomisation, rather than attempting to set minimum and maximum bounds. The GER* is estimated from the proportion of the area under a curve of permuted ghost ranges of stratigraphic unit length (MIGu) which corresponds to an MIGu value greater than the observed. Therefore:

$$GER^{*} = 1 - (Fraction\ of\ distribution \leq MIGu) \quad (12)$$

The GER* offers several advantages over other measures of stratigraphic congruence and, unlike many metrics, GER* estimates are relative to the expected values for a given topology, making it much less sensitive to differences in tree shape.

Comparing Biogeographic Congruence in Morphological and Molecular Trees

We implemented sign tests for each stratigraphic fit metric in the same way that we tested biogeographic congruence, determining whether the proportion of times molecular trees were selected over morphological ones deviated significantly from 0.5, or a 50/50 chance (Supplementary Table 7, Supplementary Figure 12). We then tested whether morphological

and molecular trees showed different median indices of stratigraphic fit (SCI, MSM*, GER, GERt and GER*) using paired two-tailed Wilcoxon signed-rank tests (Supplementary Table 8, Supplementary Figure 11). Finally, we implemented further tests of biogeographic congruence using only those clades included in the stratigraphic analyses, to determine the impact of this smaller and more taxonomically uneven sample on statistical tests of biogeographic congruence.

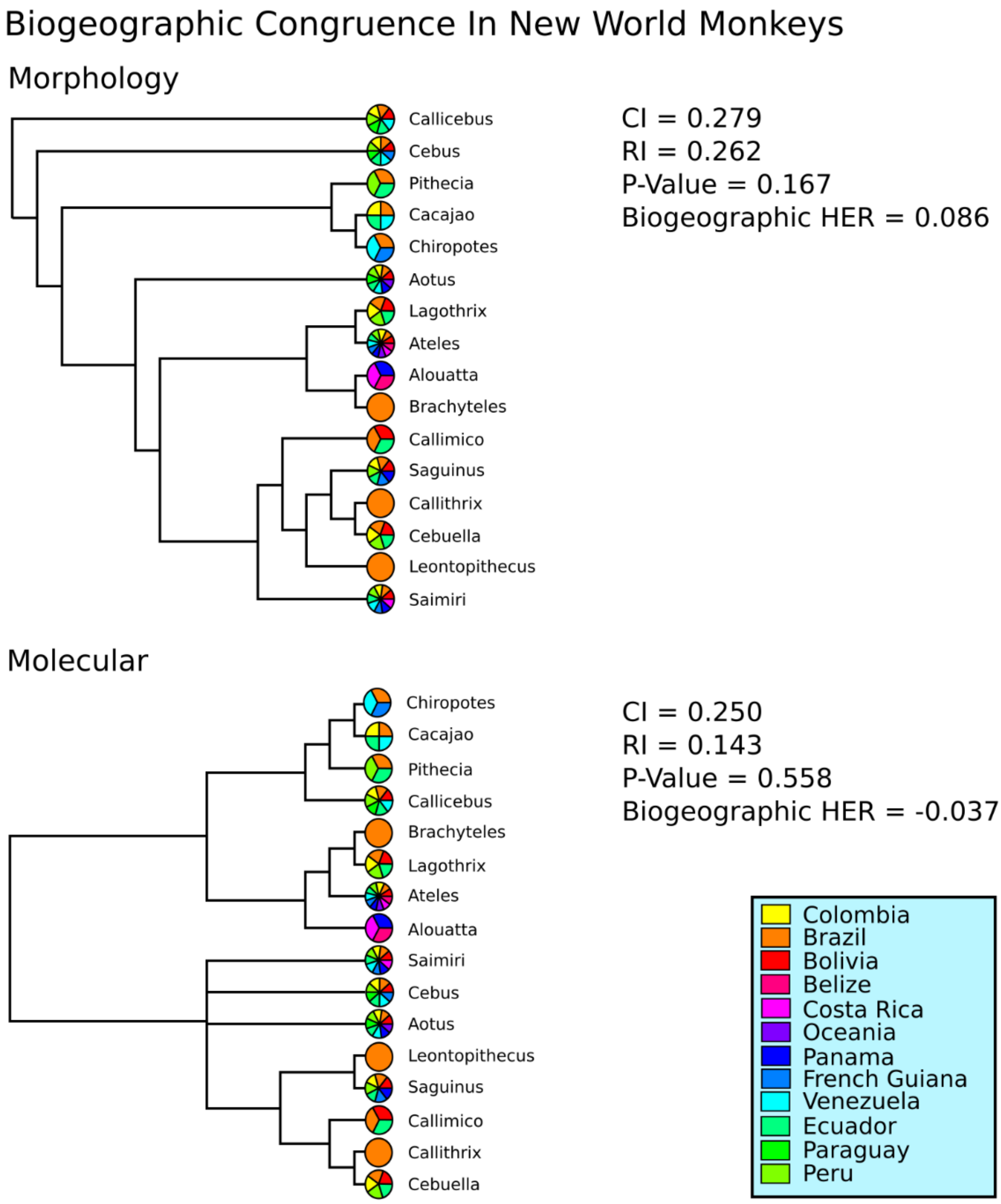

Supplementary Figure 1: Region characters mapped onto phylogenetic trees for the new world monkeys (Ceboidea). Regions coded as present are shown as pie slices for each terminal taxon. The morphological tree is from Kay 1990, the molecular tree is from Schneider *et al.* 1993.

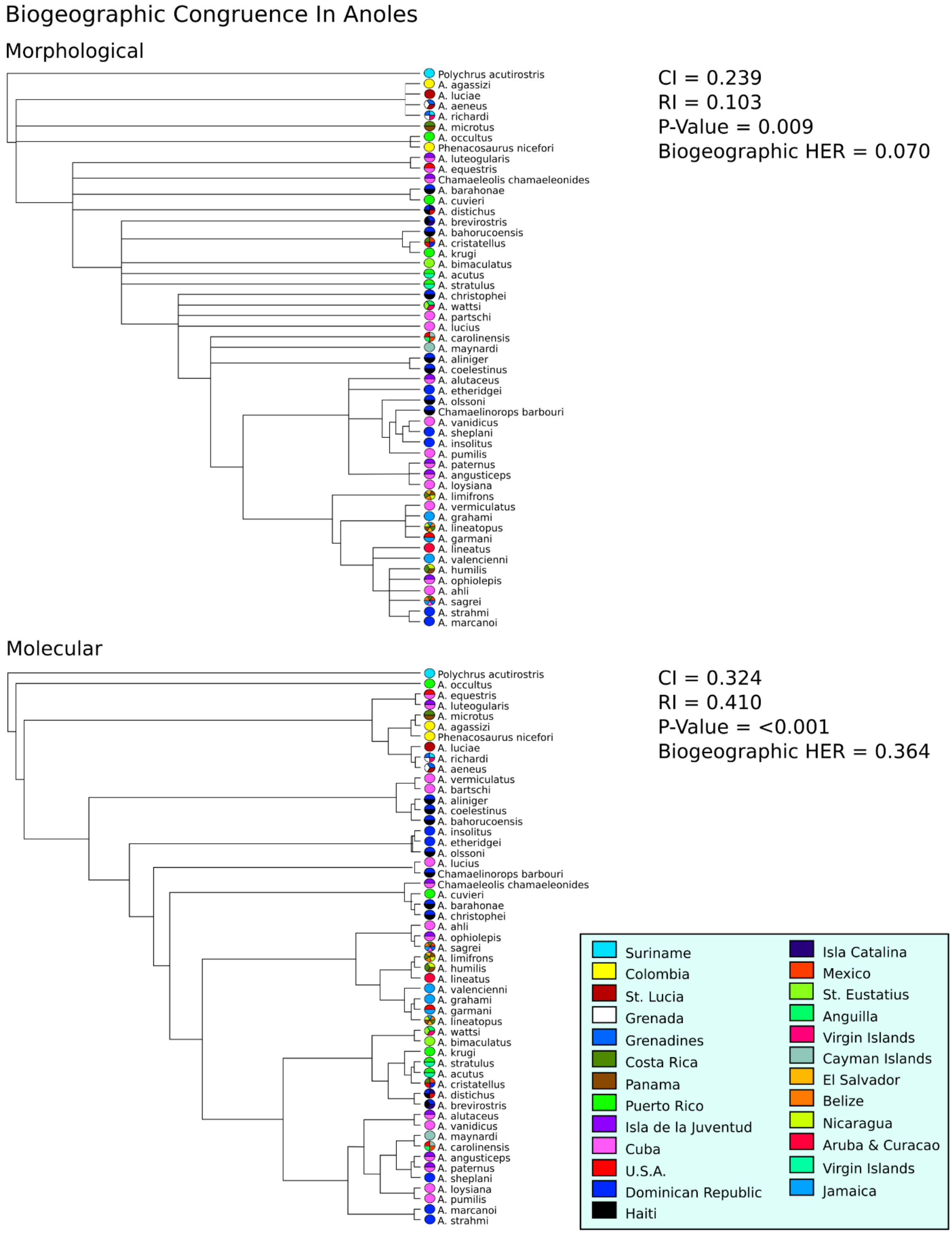

Supplementary Figure 2: Region characters mapped onto phylogenetic trees for lizards of the genus *Anolis*. Regions coded as present are shown as pie slices for each terminal taxon. Both the morphological and molecular trees are from Jackman *et al.* 1999.

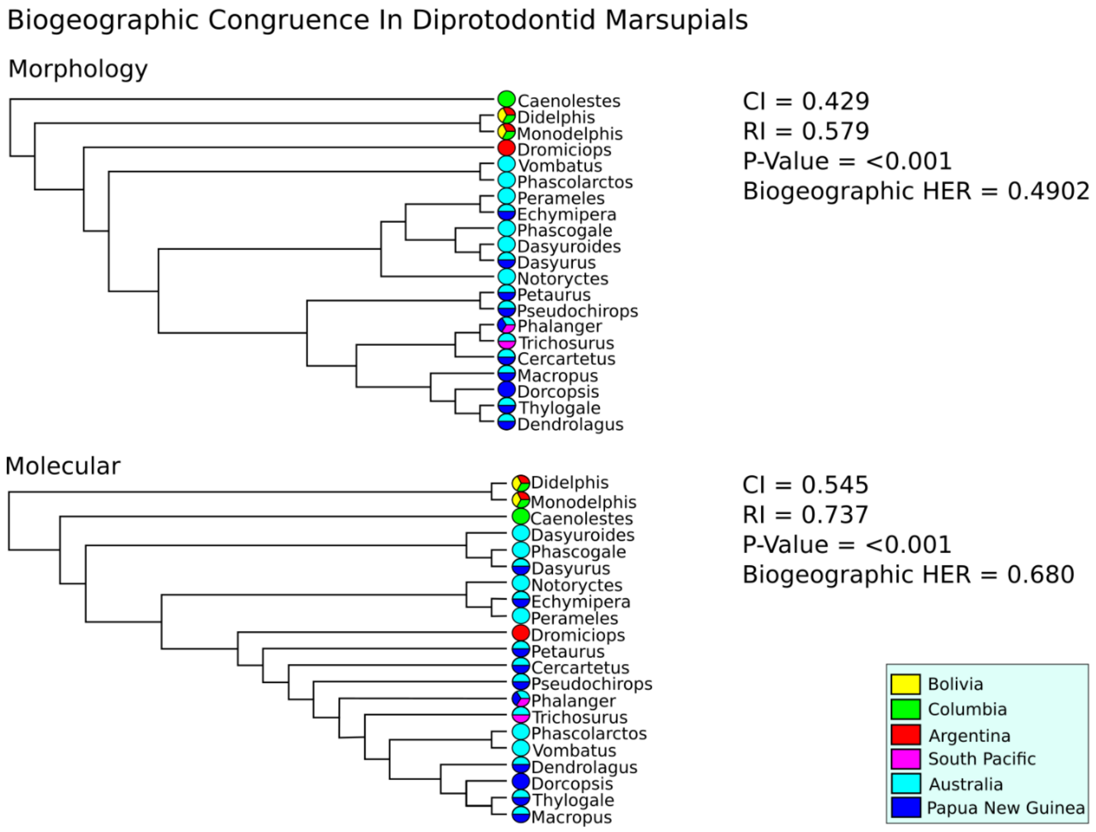

Supplementary Figure 3: Region characters mapped onto phylogenetic trees for diprotodontid marsupials (Diprotodontia). Regions coded as present are shown as pie slices for each terminal taxon. The morphological tree is from Horovitz *et al.* 2003, the molecular tree is from Meredith *et al.* 2009.

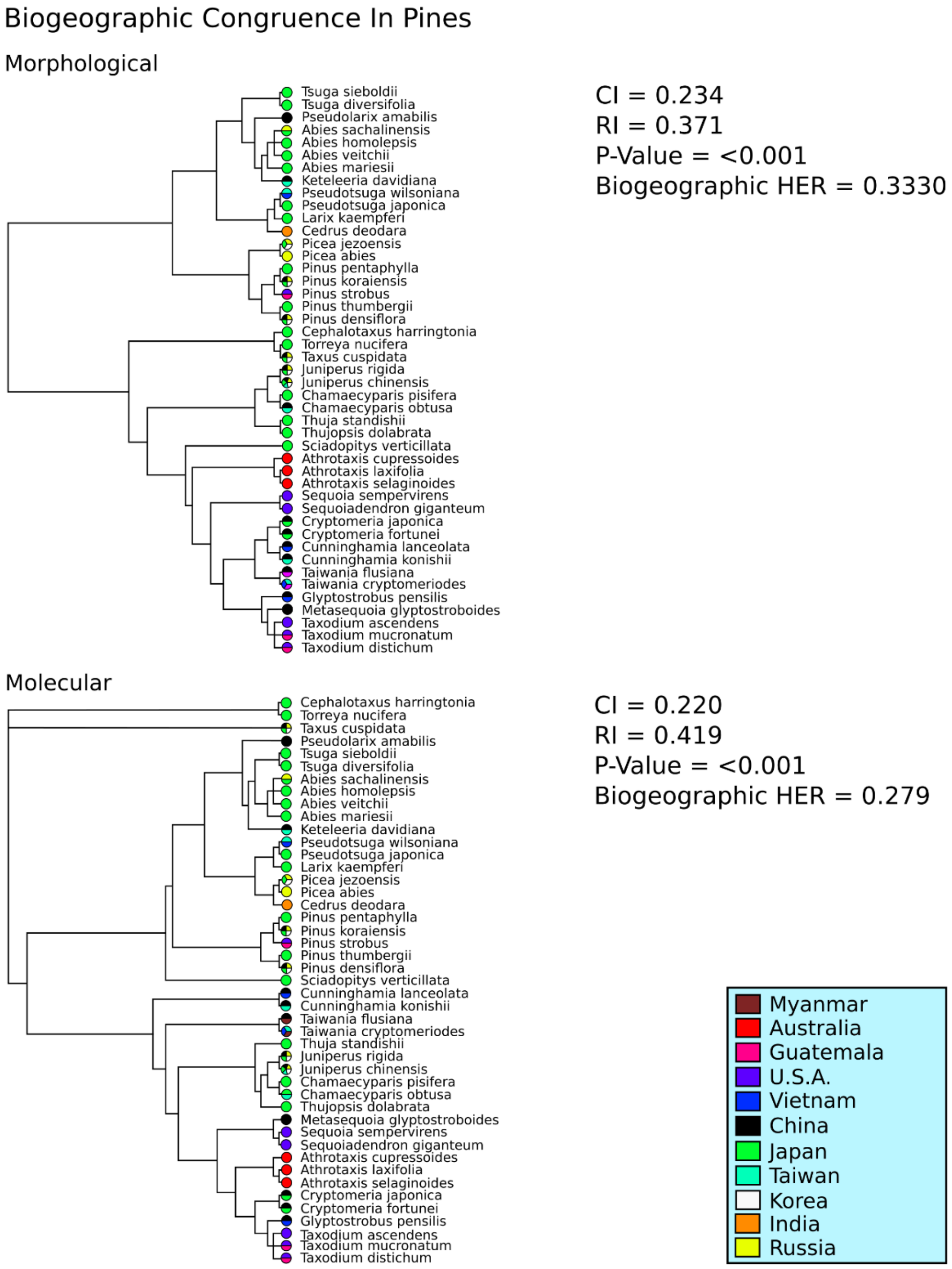

Supplementary Figure 4: Region characters mapped onto phylogenetic trees for the pine family (Pinaceae). Regions coded as present are shown as pie slices for each terminal taxon. The morphological tree is from Klymiuk & Stockey 2012, the molecular tree is from Wang *et al.* 2000.

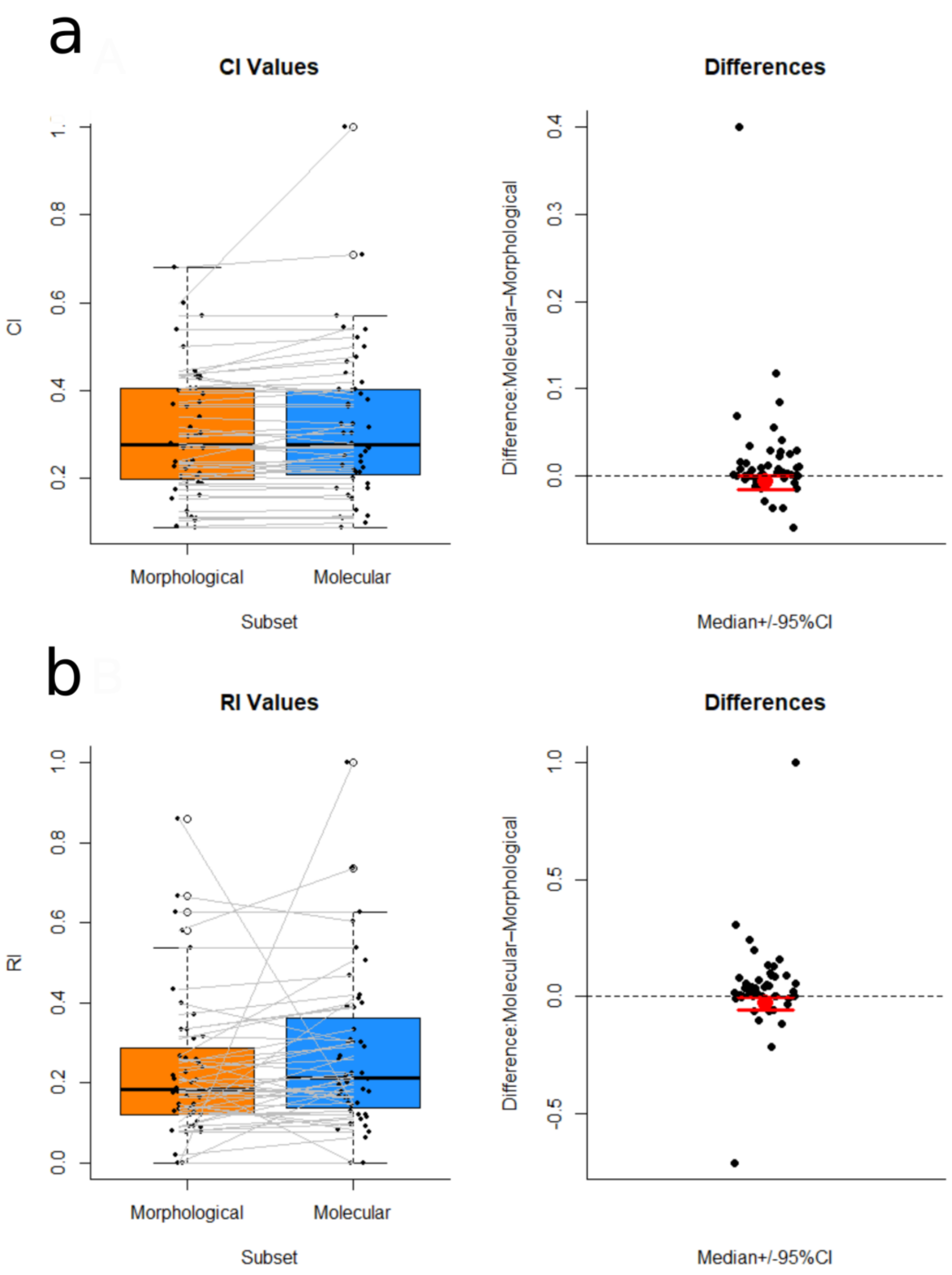

Supplementary Figure 5: boxplots of raw values and differences in values between morphological and molecular trees for (**a)** consistency index (W = 685, Z = 2.22, rc = 0.384, p =0.027) and (**b)** retention index (W = 695, Z = 2.33, rc = 0.404, p = 0.020). Boxes delimit the upper and lower quartiles of the data, while central bars are median values. Whiskers delimit plus or minus 1.5 times the inter-quartile range, from the first and third quartiles. Grey lines connect pairs of values from the same clade. Differences given are molecular values minus morphological, with positive differences indicating higher values in the molecular subsample. In the null case, difference values would be randomly distributed around the estimated pseudomedian shown in red, with upper and lower 95% confidence intervals. N = 48 biologically independent pairs of morphological and molecular phylogenies.

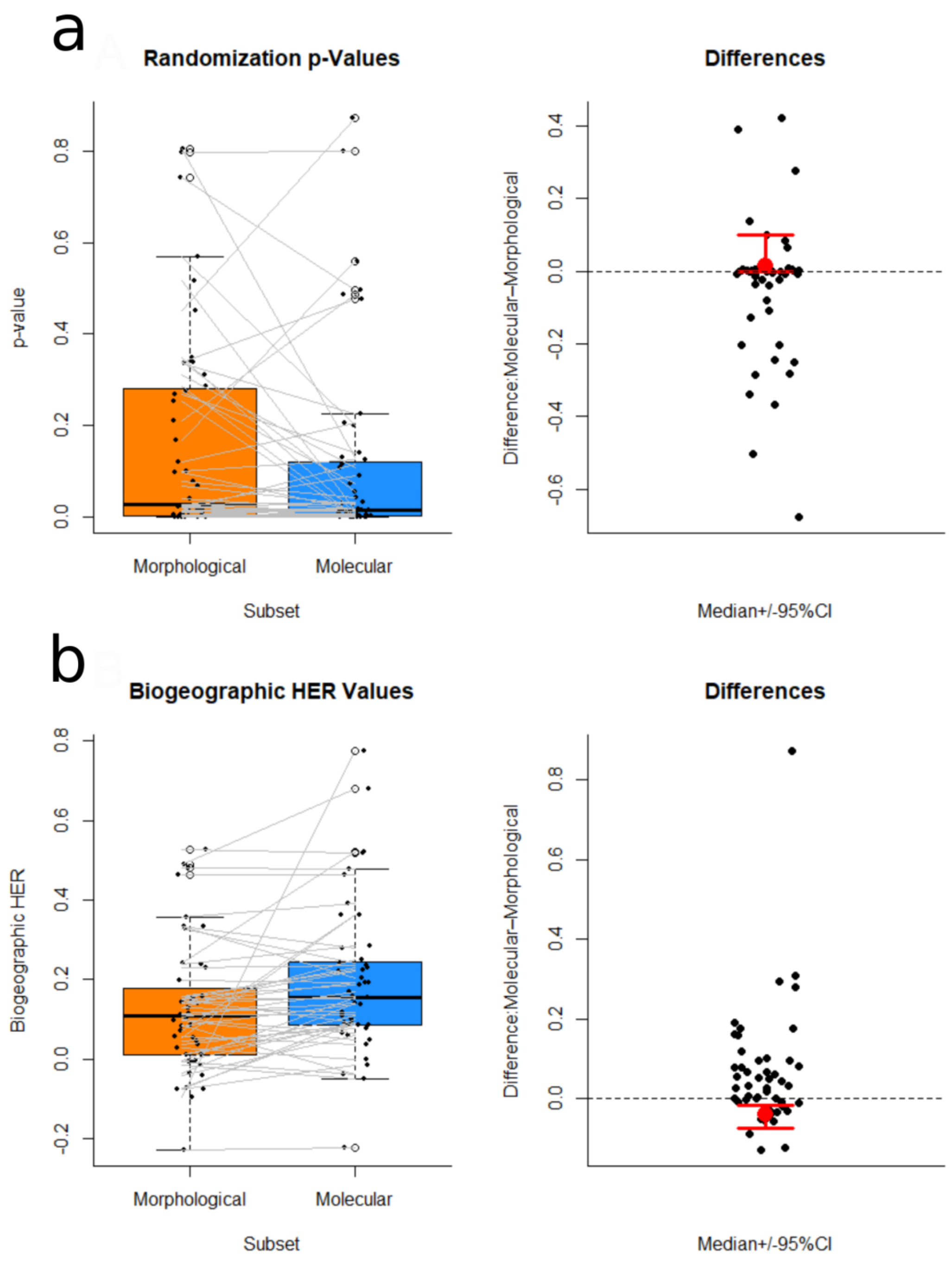

Supplementary Figure 6: Boxplots of raw values and differences in values between morphological and molecular trees for (**a**) P-values for the CI & RI randomisations (W = 373, Z = -1.63, rc = -0.279, p-value = 0.104) and (**b)** Biogeographic HER (W = 888, Z = 3.08, rc = 0.51, p-value = 0.002). Boxes delimit the upper and lower quartiles of the data, while central bars are median values. Whiskers delimit plus or minus 1.5 times the inter-quartile range, from the first and third quartiles. Grey lines connect pairs of values from the same clade. Differences given are molecular values minus morphological, with positive differences indicating higher values in the molecular subsample. In the null case, difference values would be randomly distributed around the estimated pseudomedian shown in red, with upper and lower 95% confidence intervals. N = 48 biologically independent pairs of morphological and molecular phylogenies.

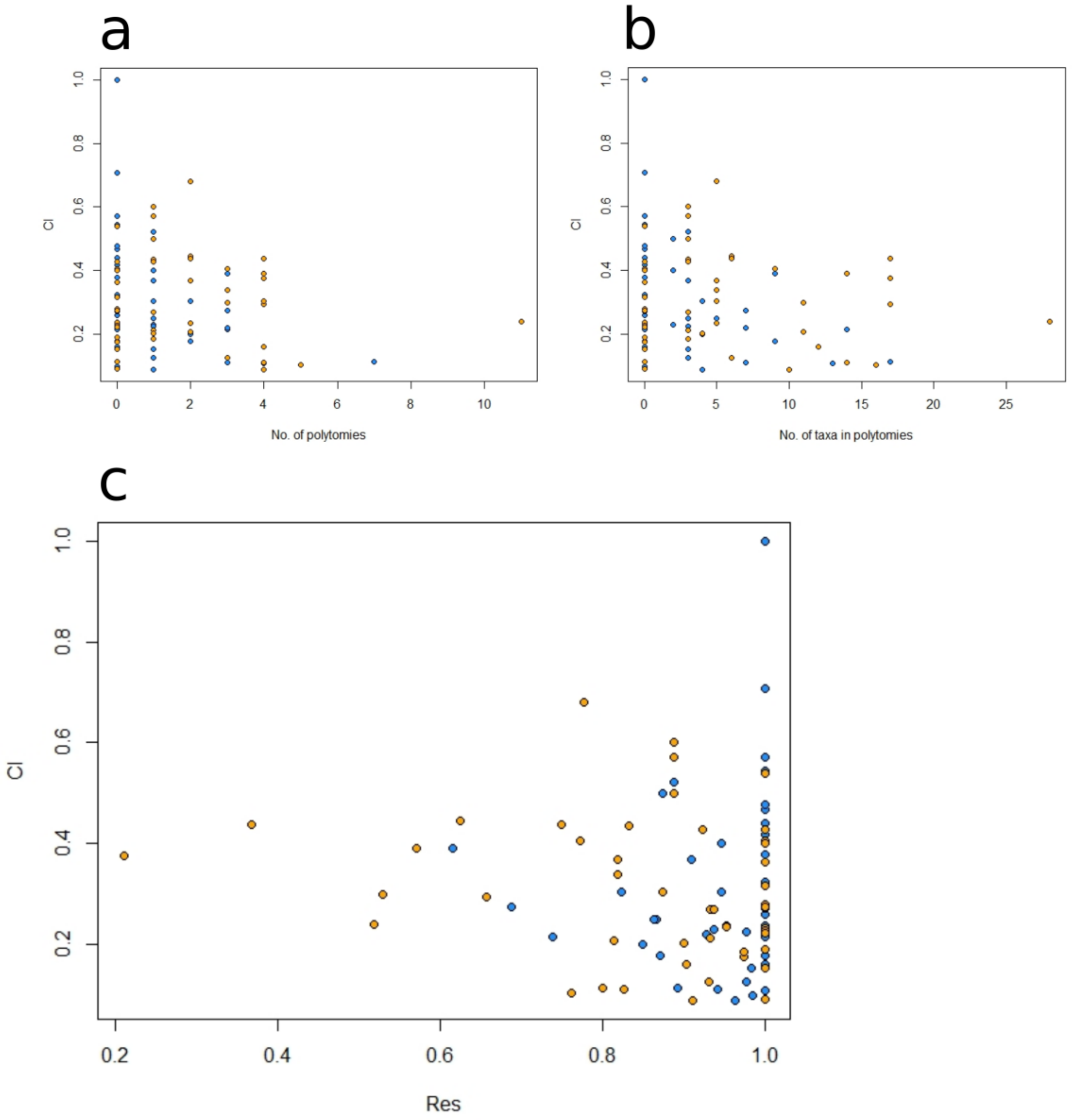

Supplementary Figure 7: Scatterplots of the consistency index (CI) (y) vs different measures of tree resolution. Datapoints for molecular trees are shown in blue, points for morphological trees are shown in orange. **(a)** Number of polytomies (x) vs. CI (y) (robust linear regression F = 3.8983, p-value = 0.05127) **(b)** Log number of taxa in polytomies (x) vs. CI (y) (robust linear regression F = 3.3472, p-value = 0.07049) **(c)** proportion of resolved nodes (x) vs. CI (y) (robust linear regression F = 0.93274, p-value = 0.3366). N = 96 morphological and molecular phylogenies.

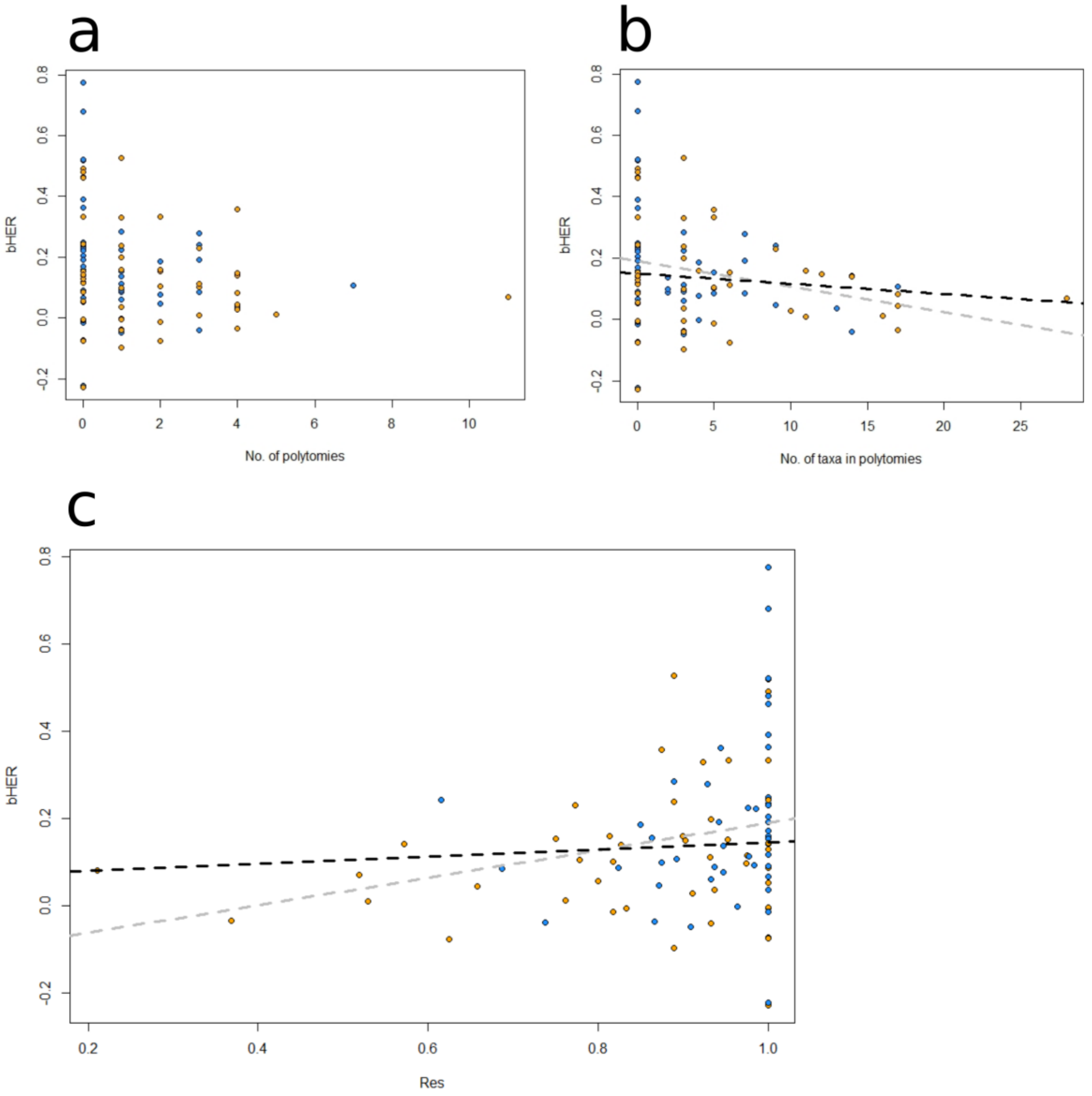

Supplementary Figure 8: Scatterplots of the biogeographic homoplasy excess ratio (bHER) (y) vs different measures of tree resolution. Datapoints for molecular trees are shown in blue, points for morphological trees are shown in orange. **(a)** Number of polytomies (x) vs. bHER (y) (robust linear regression F = 2.8438, p-value = 0.09504) **(b)** Number of taxa in polytomies (x) vs. bHER (y) (robust linear regression F = 5.0222, p-value = 0.02738) **(c)** Proportion of resolved nodes (x) vs. bHER (y) (robust linear regression F = 5.0917, p-value = 0.02636). Black dashed lines indicate regression slopes after applying inverse variance weighting. N = 96 morphological and molecular phylogenies.

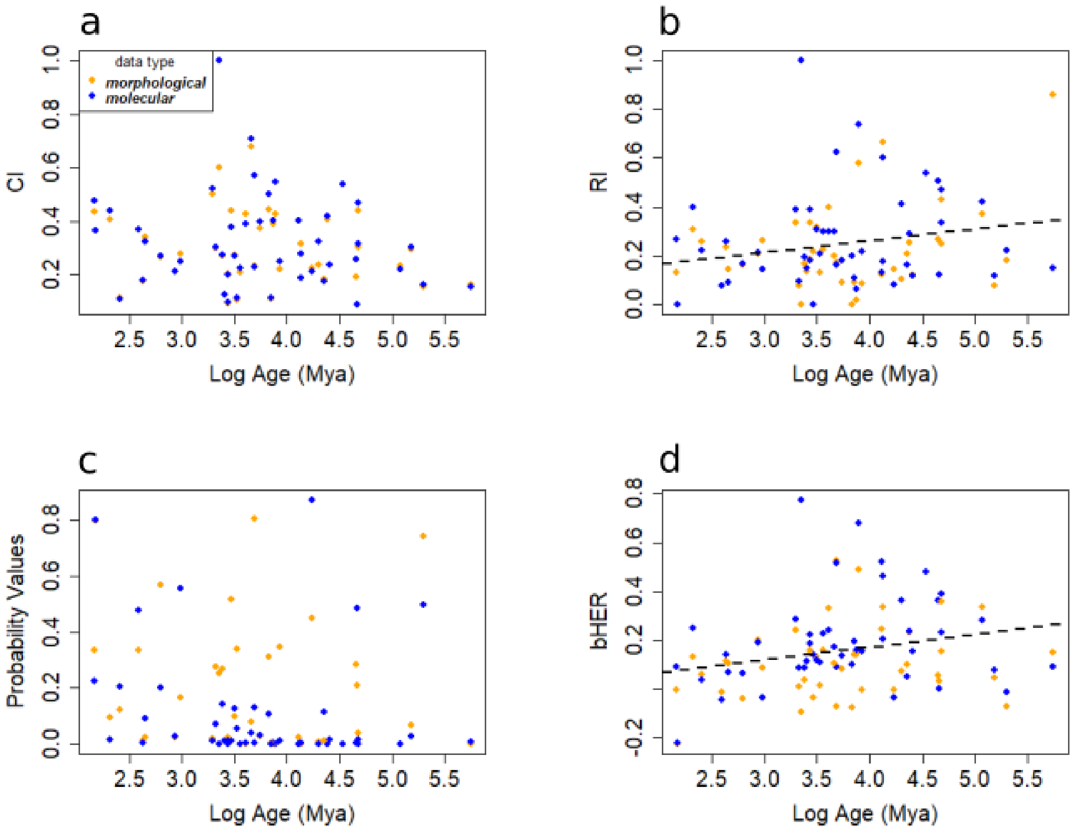

Supplementary Figure 9: Scatterplots of log median age of each clade's root node in millions of years (x) vs. biogeographic fit metrics (y). **(a)** Consistency index values (R^2 = 0.01974, p-value = 0.1722), **(b)** Retention index values (R^2 = 0.04437, p = 0.0394), **(c)** P-values of CI & RI values falling within the null distribution for randomly permuted biogeographic data (R^2 = 0.03642, p-value = 0.06252), **(d)** Biogeographic HER values (R^2 = 0.05894, p-value = 0.01716). Molecular trees in blue, morphological in orange. Significant linear regression slopes are shown as dashed lines. N = 96 morphological and molecular phylogenies.

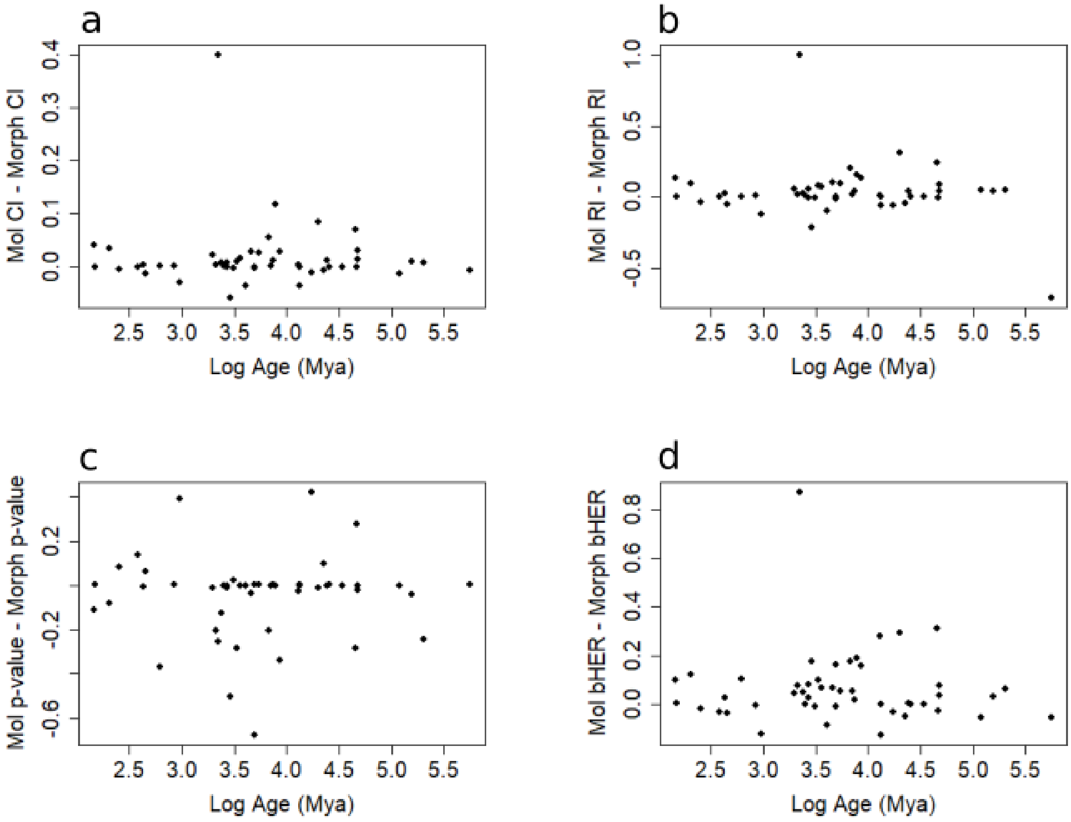

Supplementary Figure 10: Scatterplots of log median age of each clade's root node in millions of years (x) vs. differences in biogeographic fit metrics (molecular tree fit – morphological tree fit) (y). **(a)** Consistency index values (R^2 = 0.001122, p-value = 0.8212), **(b)** Retention index values (R^2 = 0.02846, p-value = 0.2517), **(c)** P-values of CI & RI values falling within the null distribution for randomly permuted biogeographic data (R^2 = 0.0006764, p-value = 0.8607), **(d)** Biogeographic HER values (R^2 = 0.001277, p-value = 0.8094). N = 96 morphological and molecular phylogenies.

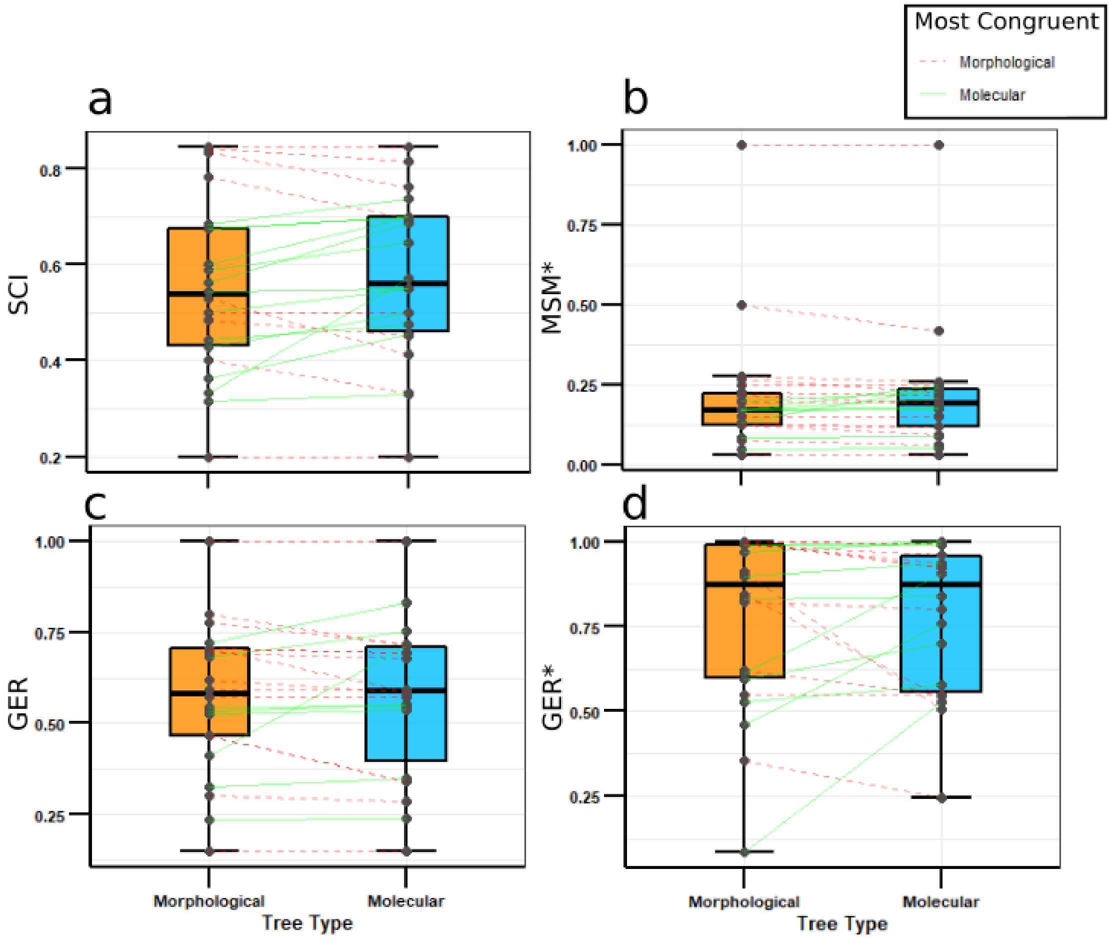

Supplementary Figure 11: Boxplots of raw values and differences in stratigraphic congruence values between morphological and molecular trees for **(a)** Stratigraphic consistency index (SCI), **(b)** Modified Manhattan stratigraphic measure (MSM*), **(c)** Gap excess ratio (GER) and **(d)** Modified gap excess ratio (GER*). Boxes delimit the upper and lower quartiles of the data, while central bars are median values. Whiskers delimit plus or minus 1.5 times the inter-quartile range, from the first and third quartiles. Coloured lines connected pairs of values from the same clade, where red dashed lines indicate the morphological tree is most biogeographically congruent and green solid lines indicate the molecular tree is most biogeographically congruent. n = 23 biologically independent pairs of morphological and molecular phylogenies.

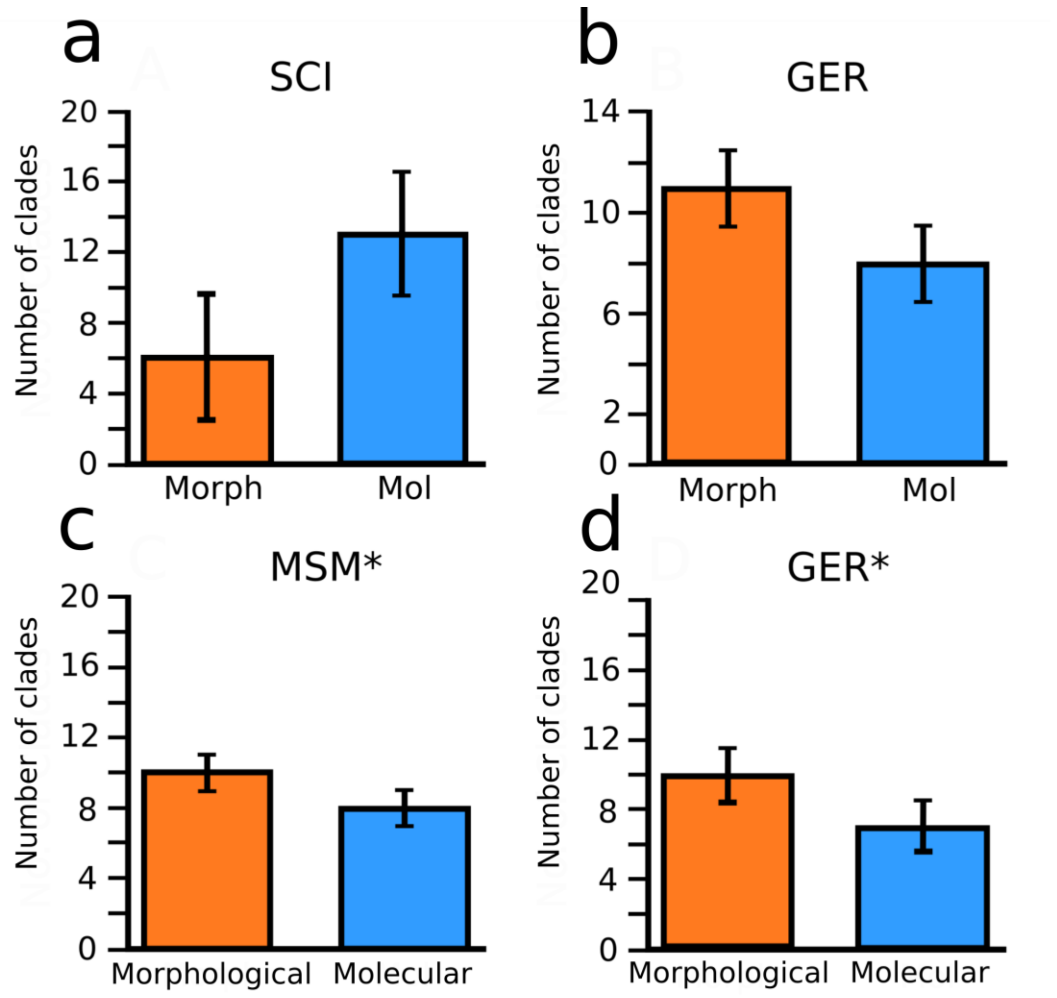

Supplementary Figure 12: Comparison of the number of trees in each sample (morphological or molecular) with greater stratigraphic fit. **(a)** Stratigraphic consistency index (SCI), **(b)** Gap excess ratio (GER), **(c)** Modified Manhattan stratigraphic measure (MSM*), **(d)** Modified gap excess ratio (GER*). Bars show the number of clades in each subset, with binomial confidence intervals calculated using the approach of Clopper & Pearson [30] . n = 23 biologically independent pairs of morphological and molecular phylogenies.

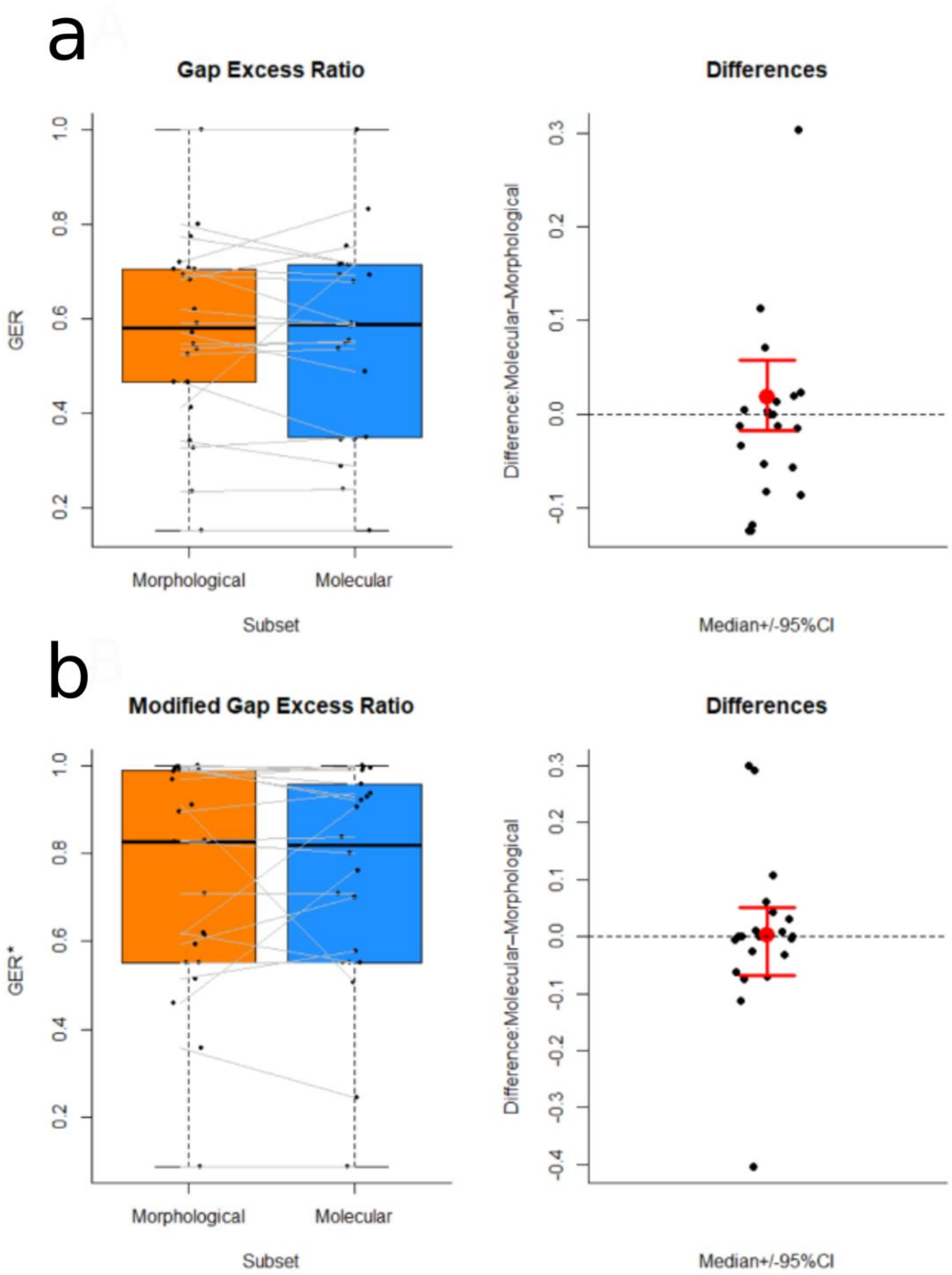

Supplementary Figure 13: Boxplots of stratigraphic consistency and differences in stratigraphic consistency between morphological and molecular trees for **(a)** Gap excess ratio (GER) and **(b)** Modified gap excess ratio (GER*). Boxes delimit the first and third quartiles of the data, while central bars are median values. Whiskers delimit plus or minus 1.5 times the inter-quartile range, from the first and third quartiles. Grey lines connect pairs of values from the same clade. Differences given are molecular values minus morphological, with positive differences indicating higher values in the molecular subsample. In the null case, difference values would be randomly distributed around the estimated pseudomedian shown in red, with upper and lower 95% confidence intervals. n = 23 biologically independent pairs of morphological and molecular phylogenies.

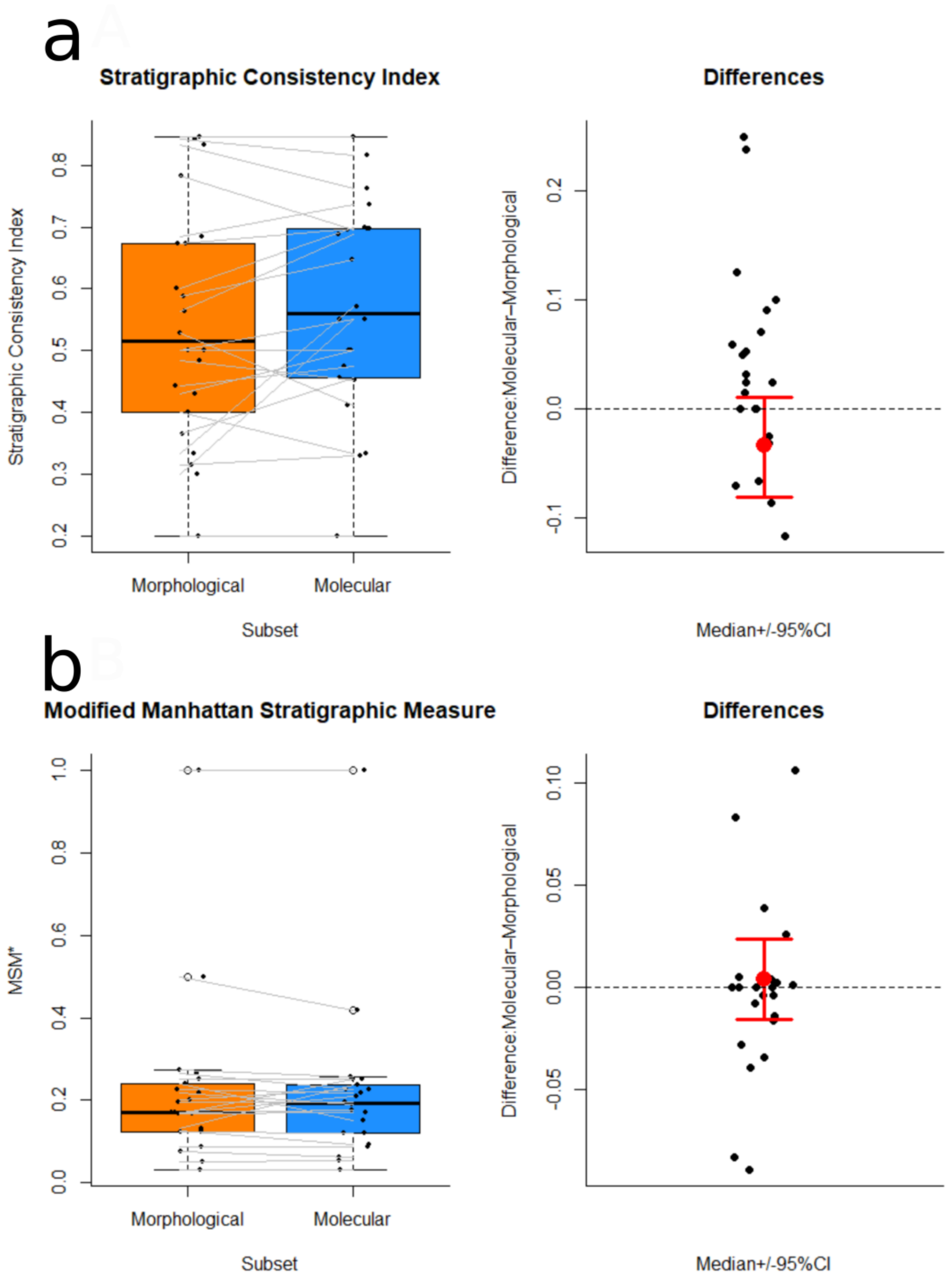

Supplementary Figure 14: Boxplots of raw values and differences in stratigraphic consistency between morphological and molecular trees for **(a)** Stratigraphic consistency index and **(b)** Modified Manhattan stratigraphic measure. Boxes delimit the first and third quartiles of the data, while central bars are median values. Whiskers delimit plus or minus 1.5 times the inter-quartile range, from the first and third quartiles. Grey lines connect pairs of values from the same clade. Differences given are molecular values minus morphological, with positive differences indicating higher values in the molecular subsample. In the null case, difference values would be randomly distributed around the estimated pseudomedian shown in red, with upper and lower 95% confidence intervals. n = 23 biologically independent pairs of morphological and molecular phylogenies.

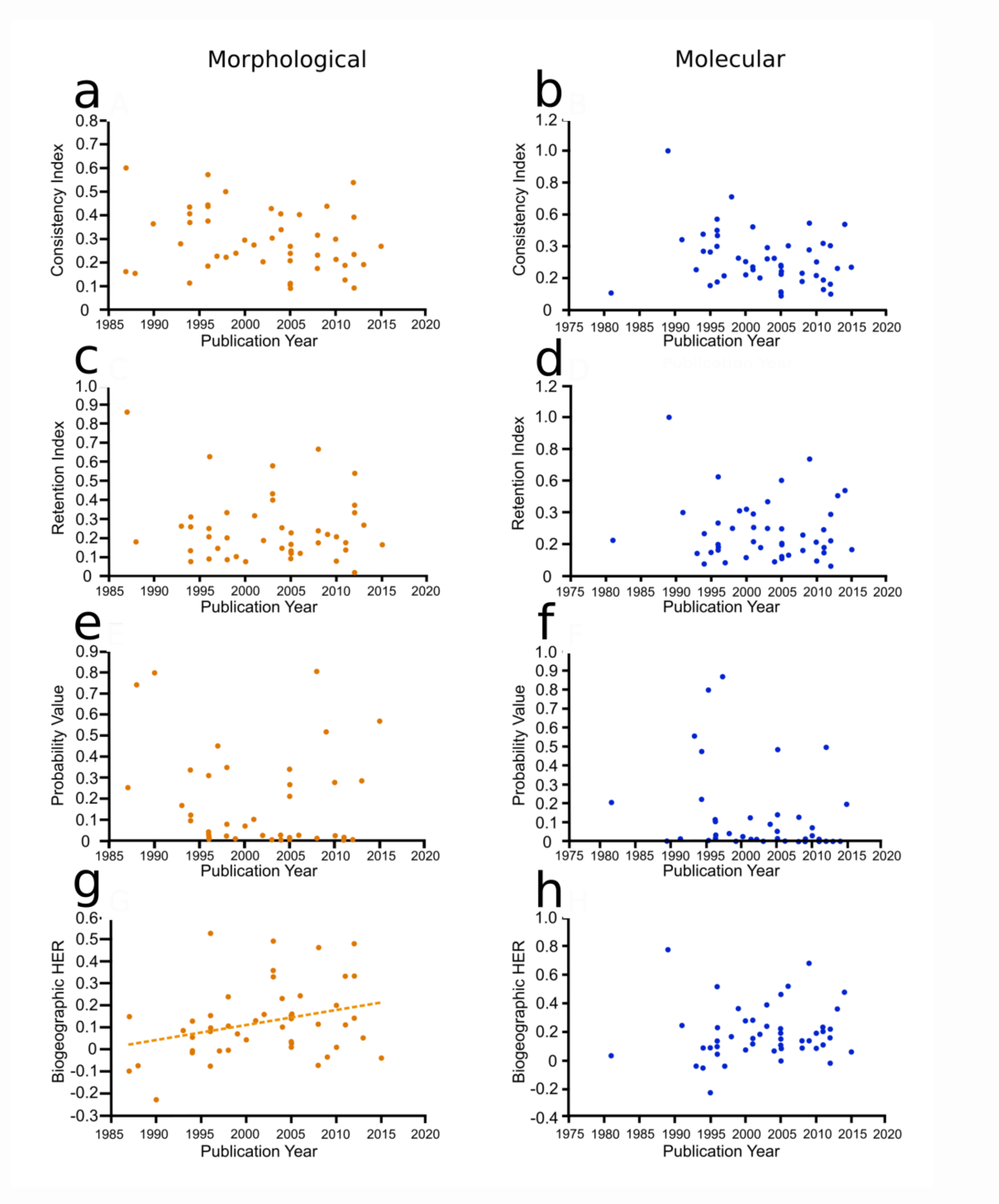

Supplementary Figure 15: Scatterplots of publication year (x) vs. biogeographic fit metrics (y), with outliers removed. Dotted trendlines indicate statistically significant linear regression lines. **(a)** Consistency index values for morphological trees, **(b)** Consistency index values for molecular trees, **(c)** Retention index values for morphological trees, **(d)** Retention index values for molecular trees, **(e)** P-values of CI & RI values falling within the null distribution for morphological trees, **(f)** P-values of CI & RI values falling within the null distribution for molecular trees, **(g)** Biogeographic HER values for morphological trees ($R^2 = 0.1$, p-value = 0.029), **(h)** Biogeographic HER values for molecular trees. N = 96 morphological and molecular phylogenies.

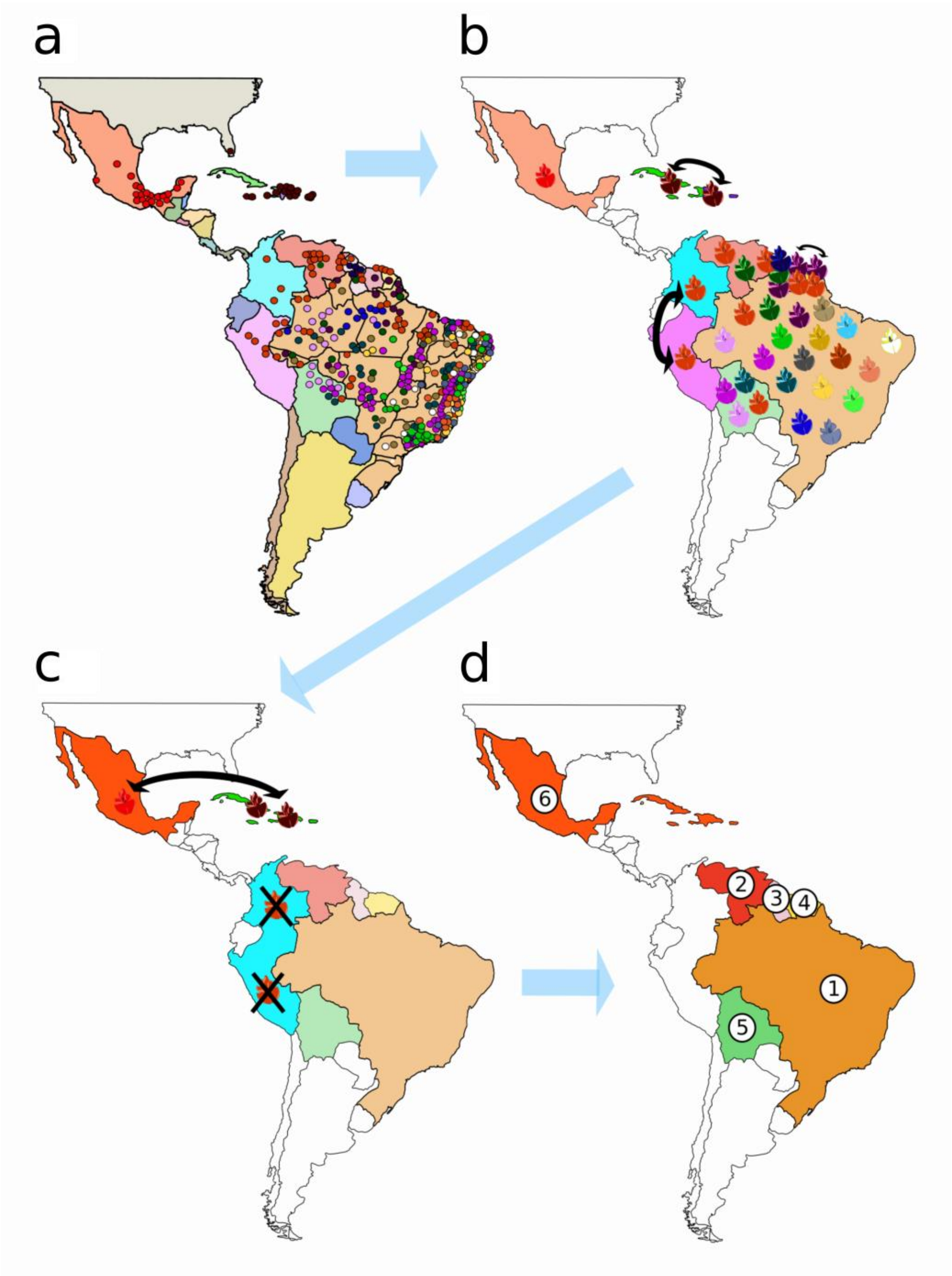

Supplementary Figure 16: Characterising biogeographic regions. Taxa of the flowering plant genus *Andira*, with occurrence data taken from GBIF. **(a)** Occurrence data for each terminal/taxon are collated from online repositories (GBIF, IUCN Redlist). Different coloured points represent different taxa, and the delineated coloured areas are countries**. (b)** The occurrence data are used to codify taxon presence/absences in each geographical unit (countries in this case). Coloured areas are countries containing taxa, with taxon presence shown by coloured symbols. Arrows indicate adjacent countries with identical taxon sets**. (c)** Adjacent countries with identical taxon sets are combined into new regions. In this example, both Mexico (orange) and the Caribbean (green) contain only endemic taxa and are therefore combined (geographically closest). The Colombia+Peru region (light blue) has only one taxon which is also found in other regions and is therefore removed. **(d)** Final numbered regions with unique taxon compositions.

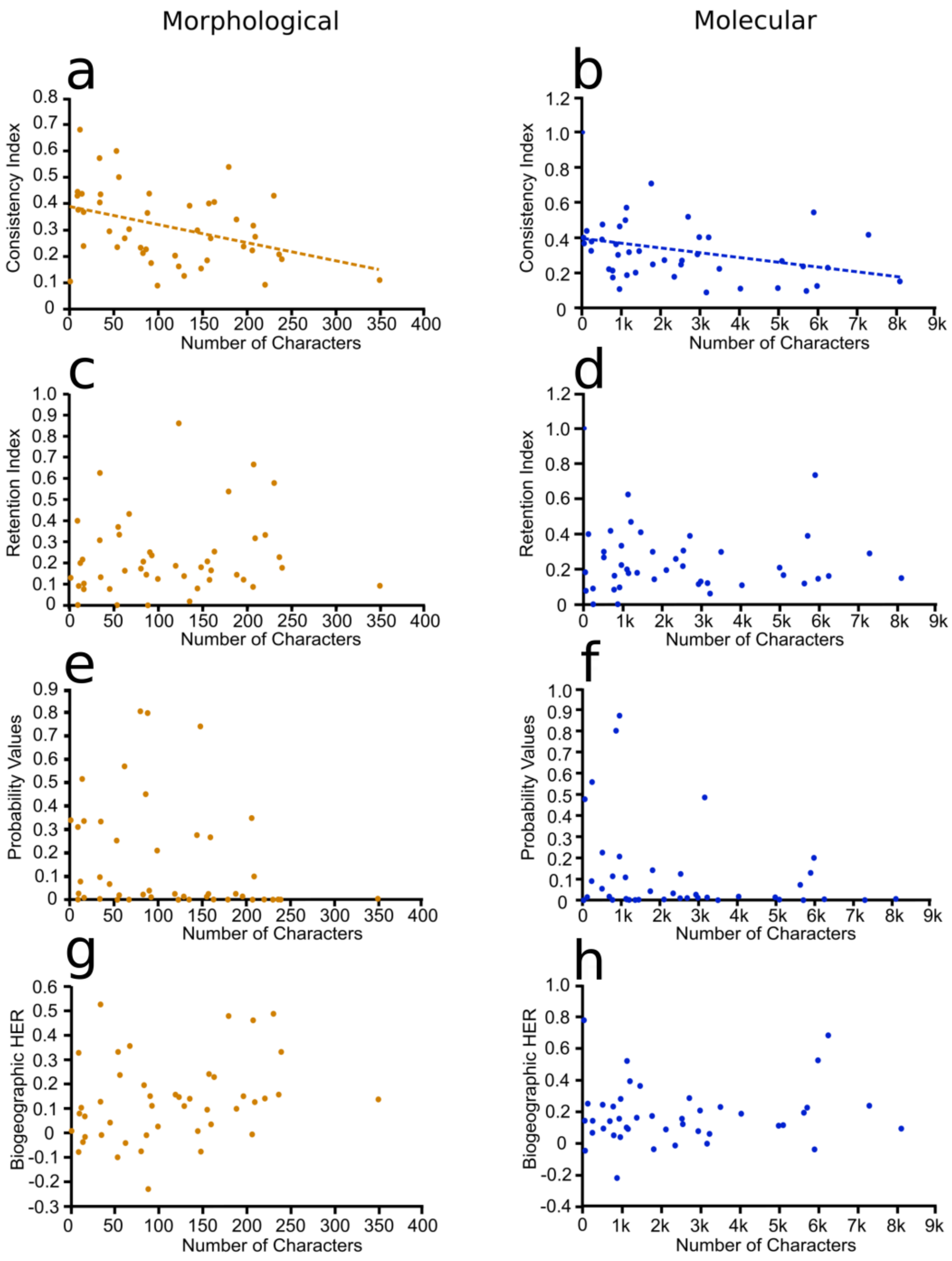

Supplementary Figure 17: Scatterplots of the number of phylogenetic characters (x) vs. biogeographic fit metrics (y), with outliers removed. Dotted trendlines indicate statistically significant linear regression lines. **(a)** Consistency index values for morphological trees (R^2 = -0.389, p-value = 0.008), **(b)** Consistency index values for molecular trees (R^2 = -0.332, p-value = 0.030), **(c)** Retention index values for morphological trees, **(d)** Retention index values for molecular trees, **(e)** P-values of CI & RI values falling within the null distribution for morphological trees, **(f)** P-values of CI & RI values falling within the null distribution for molecular trees, **(g)** Biogeographic HER values for morphological trees, **(h)** Biogeographic HER values for molecular trees. N = 96 morphological and molecular phylogenies.

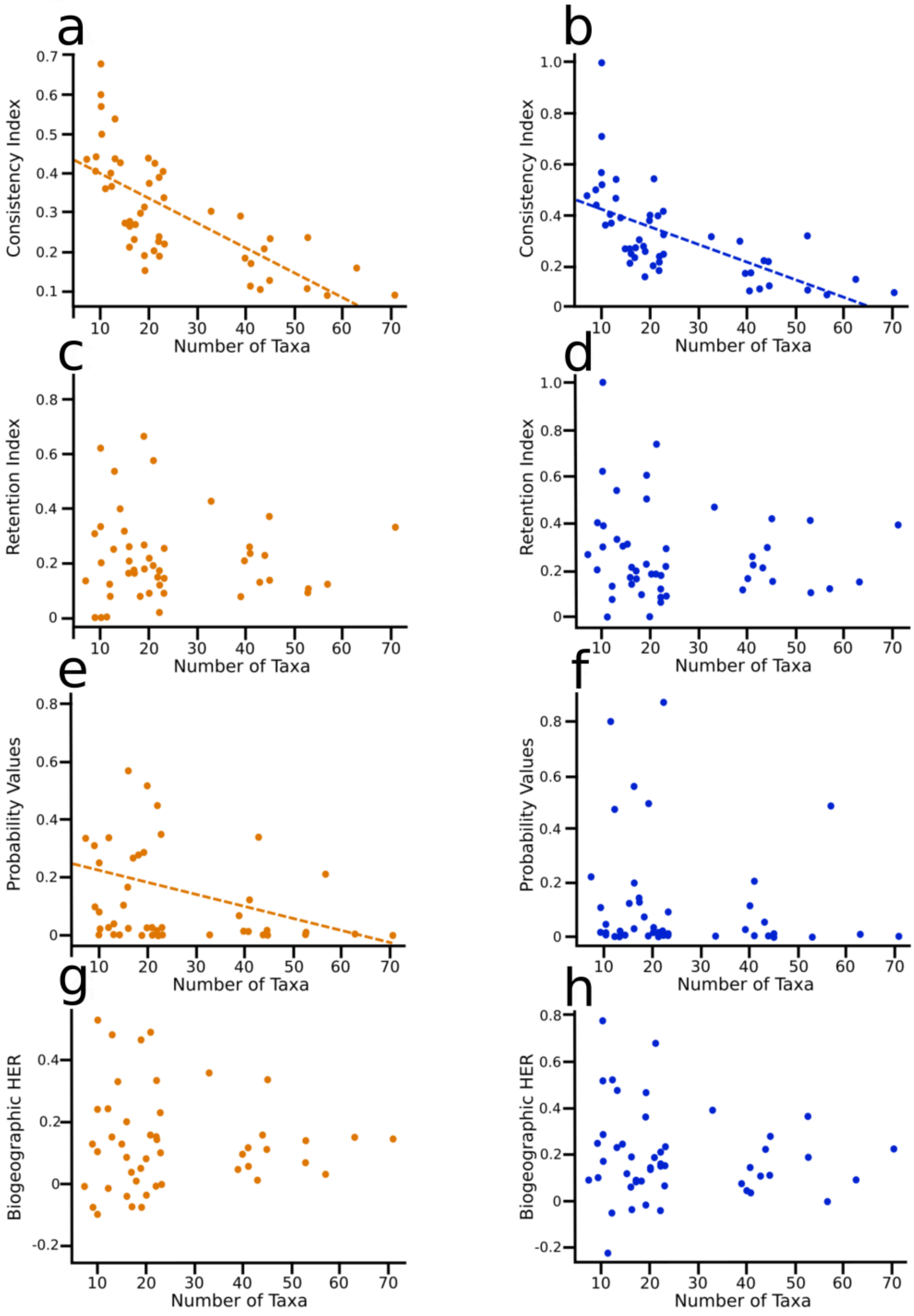

Supplementary Figure 18: Scatterplots of the number of terminal taxa (x) vs. biogeographic fit metrics (y), with outliers removed. Dotted trendlines indicate statistically significant linear regression lines. **(a)** Consistency index values for morphological trees (R^2 = -0.7043572, p-value = $2.318\text{x}10^{8}$), **(b)** Consistency index values for molecular trees (R^2 = -0.6269879, p-value = $1.859\text{x}10^{6}$), **(c)** Retention index values for morphological trees, **(d)** Retention index values for molecular trees, **(e)** P-values of CI & RI values falling within the null distribution for morphological trees, **(f)** P-values of CI & RI values falling within the null distribution for molecular trees, **(g)** Biogeographic HER values for morphological trees, **(h)** Biogeographic HER values for molecular trees. N = 96 morphological and molecular phylogenies.

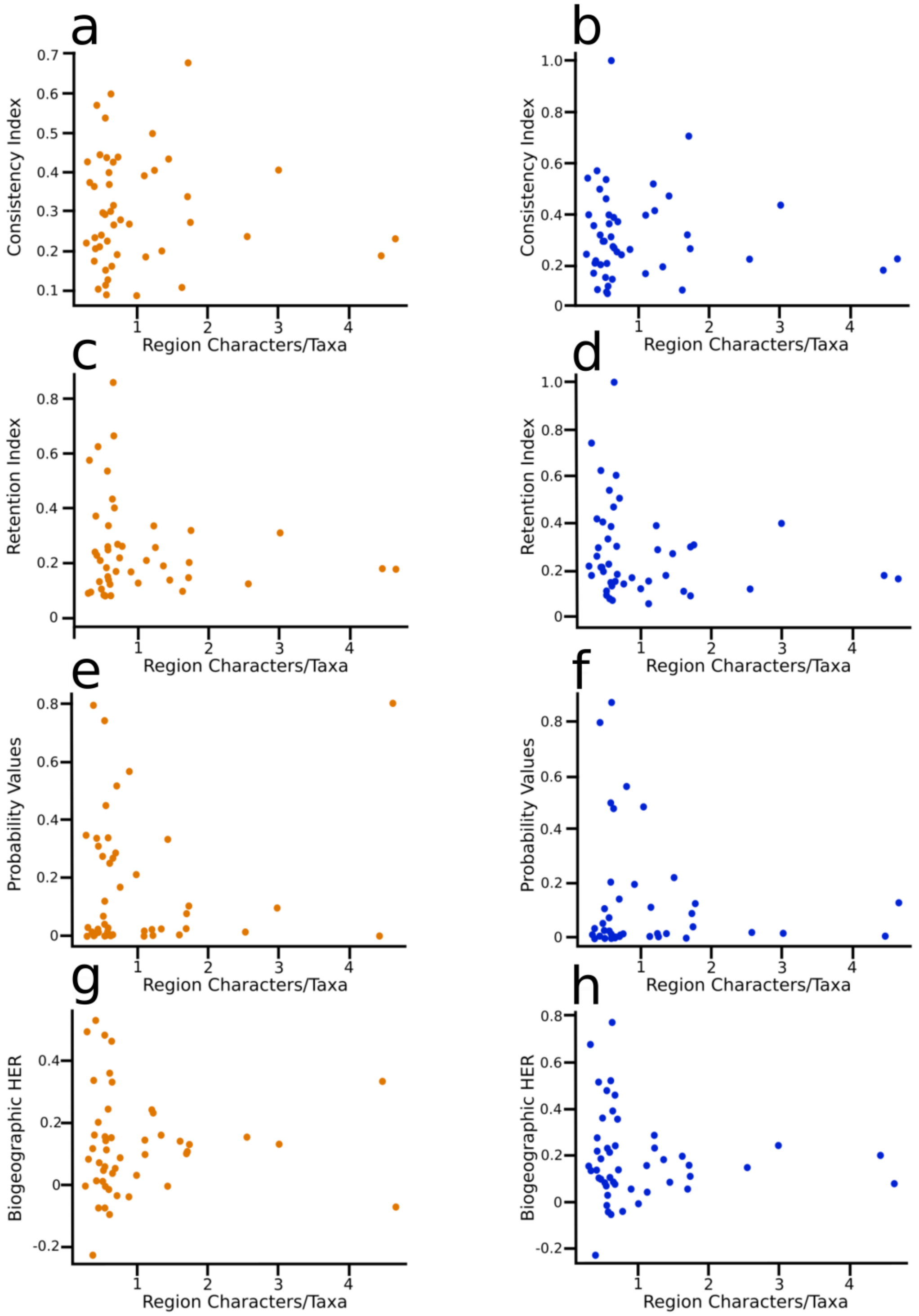

Supplementary Figure 19: Scatterplots of the ratio of region characters to terminal taxa (x) vs. biogeographic fit metrics (y), with outliers removed. **(a)** Consistency index values for morphological trees (R^2 = 0.151, p-value = 0.008), **(b)** Consistency index values for molecular trees (R^2 = 0.110, p-value = 0.030), **(c)** Retention index values for morphological trees, **(d)** Retention index values for molecular trees, **(e)** P-values of CI & RI values falling within the null distribution for morphological trees, **(f)** P-values of CI & RI values falling within the null distribution for molecular trees, **(g)** Biogeographic HER values for morphological trees, **(h)** Biogeographic HER values for molecular trees. N = 96 morphological and molecular phylogenies.

Dataset	Metric	Sample size	Morphology higher	Success p-value	Molecular higher	Success p-value	Binomial p-value
Whole dataset	CI	48	15	0.313	29	0.604	0.193
	RI	48	14	0.292	30	0.625	0.111
	CI & RI p-value	48	20	0.417	25	0.521	0.885
	bHER	48	15	0.313	33	0.688	0.013
All cases with difference	CI	44	15	0.341	29	0.659	0.049
	RI	44	14	0.318	30	0.682	0.020
	CI & RI p-value	45	20	0.444	25	0.556	0.552
	bHER	48	15	0.313	33	0.688	0.013
Either fit significantly different from random	CI	33	9	0.273	22	0.667	0.014
	RI	32	7	0.219	23	0.719	0.005
	CI & RI p-value	33	13	0.394	17	0.515	0.999
Either fit significantly different from random & one fit better than the other	CI	31	9	0.290	22	0.710	0.029
	RI	30	7	0.233	23	0.767	0.005
	CI & RI p-value	30	13	0.433	17	0.567	0.585

Supplementary Table 1: Results of sign tests for the number of cases molecular trees are selected over morphological trees based on the following measures of biogeographic fit: consistency index (CI), retention index (RI), CI & RI randomisation p-values (CI & RI p-value) and biogeographic homoplasy excess ratio (bHER). Tests were carried out on the whole dataset, only those datasets where there was a difference in fit values, only those datasets in which at least one of the CI or RI values significantly differed from a distribution of 10,000 randomisations and only those datasets in which at least one of the CI or RI values significantly differed from a distribution of 10,000 randomisations and there was a difference in fit value. Statistically significant results are highlighted in green. N = 48 biologically independent pairs of morphological and molecular phylogenies.

Clade	Observed incompatibilities	Mean random	Error	Normal deviate	95% cutoff	P-value	Incompatibility excess ratio (1)	Incompatibility excess ratio (2)
Anas	171	213.464	7.352	5.776	200	0.001	0.199	0.145
Andira	5	5.025	1.83	0.014	2	0.626	0.005	-1.5
Anolis	36	63.123	6.187	4.384	54	0.001	0.43	0.333
Arctoidea	2191	2323.012	19.123	6.903	2291	0.001	0.057	0.044
Bothropis	55	58.739	4.145	0.902	52	0.228	0.064	-0.058
Canidae	206	281.81	11.963	6.337	263	0.001	0.269	0.217
Ceboidea	36	39.513	2.734	1.285	35	0.14	0.089	-0.029
Chiroptera 1	38	56.334	2.621	6.996	52	0.001	0.325	0.269
Chiroptera 2	3251	3754.94	24.156	20.862	3716	0.001	0.134	0.125
Chrysochloridae	7	11.835	2.04	2.37	9	0.017	0.409	0.222
Crocodylia	75	119.087	8.857	4.978	105	0.001	0.37	0.286
Cupressaceae	22	52.349	5.581	5.438	44	0.001	0.58	0.5
Didelphidae	132	132.128	3.713	0.035	126	0.536	0.001	-0.048
Didelphinae	190	178.347	4.572	-2.549	171	0.993	-0.065	-0.111
Diprotodontia	3	7.082	1.727	2.364	4	0.015	0.576	0.25
Drosophila	0	2.6	1.201	2.165	1	0.038	1	1
Echymyidae	50	57.139	4.09	1.745	51	0.049	0.125	0.02
Epicrates	0	1.301	0.853	1.526	0	0.171	1	-
Erinaceidae	-	-	-	-	-	-	-	-
Eutheria	34	63.167	1.636	17.831	60	0.001	0.462	0.433
Feliformia	2574	2513.461	20.708	-2.923	2481	0.998	-0.024	-0.037
Glires	1123	946.181	17.175	-10.295	919	1	-0.187	-0.222
Heliconius	127	164.109	4.373	8.487	157	0.001	0.226	0.191
Iguanidae 1	57	89.273	6.131	5.264	80	0.001	0.362	0.288
Iguanidae 2	5	8.94	2.225	1.771	5	0.057	0.441	0
Josiini	54	42.214	3.347	-3.521	37	0.999	-0.279	-0.459
Krigia	18	27.394	3.173	2.96	22	0.006	0.343	0.182
Macropodidae	13	18.683	1.333	4.262	16	0.003	0.304	0.188
Megachiroptera	27	59.046	4.821	6.647	51	0.001	0.543	0.471
Mormoopidae	156	235.767	5.888	13.547	226	0.001	0.338	0.31
Neckeraceae	23	24.673	4.08	0.41	18	0.398	0.068	-0.278
Ophraella	5	2.626	1.191	-1.993	1	0.992	-0.904	-4
Opluridae	0	0	0	0	0	1	-	-
Phrynosomatidae	405	508.664	13.058	7.939	488	0.001	0.204	0.17
Phyllostomidae 1	543	500.27	9.537	-4.48	484	0.999	-0.085	-0.122
Phyllostomidae 2	274	273.478	7.254	-0.072	262	0.558	-0.002	-0.046
Physalaemus	6	6.447	1.5	0.298	4	0.515	0.069	-0.5
Pinacea	13	25.049	2.833	4.253	21	0.001	0.481	0.381
Pinales	477	393.174	11.889	-7.051	373	1	-0.213	-0.279
Platynini	10	10.241	1.655	0.146	8	0.55	0.024	-0.25
Plecotini	17	25.257	3.215	2.568	20	0.014	0.327	0.15
Ratites	1	10.142	2.207	4.142	7	0.001	0.901	0.857
Rhopalocera	1302	1319.906	10.571	1.694	1304	0.041	0.014	0.002
Sciuridae	13	19.25	2.839	2.202	15	0.021	0.325	0.133
Sphenisciformes	27	36.35	3.243	2.883	31	0.003	0.257	0.129
Sphenostylis	12	10.262	2.14	-0.812	7	0.854	-0.169	-0.714
Squamata	39	44.541	0.683	8.111	43	0.001	0.124	0.093
Talpidae	5	9.543	2.182	2.081	6	0.024	0.476	0.167

Supplementary Table 2: Results of permutation tail probability (PTP) tests of character covariance in the biogeographic matrices of each clade in this study. Clade: the phylogenetic group analysed. Observed incompatibilities: number of conflicting binary character states in the minimum length cladogram produced from the region matrix. Mean random: average number of conflicting binary character states in minimum length cladograms produced from 999 randomisations of the region matrix. Error: standard error for the mean of each distribution of random matrices. Normal deviate: standard normal deviate of the number of observed incompatibilities relative to the mean random number. 95% cutoff: number of observed incompatibilities which 95% of random values are equal to or less than. P-value: probability the number of observed incompatibilities is less than the range of random values. Incompatibility excess ratio: equivalent to a homoplasy excess ratio for region character inconsistencies on observed and random minimum length cladograms.

Clade	Data type	Phylogenetic characters	Publication year	Number of taxa	Number of region characters	Colless's index (*C*)	Root node age (Mya)	Res
Anas	Morph	34	1994	9	27	0.357143	10.1	1
	Mol	119	1991	9	27	0.321429	10.1	1
Andira	Morph	10	1996	20	6	0.087719	42	0.210526
	Mol	38	1996	20	6	0.222222	42	0.947368
Anolis	Morph	16	1999	53	24	0.055807	74	0.519231
	Mol	1,455	1999	53	24	0.239065	74	1
Arctoidea	Morph	80	2008	17	79	0.308333	40	1
	Mol	6243	2008	17	79	0.375	40	0.9375
Bothropis	Morph	92	2008	41	15	0.264103	13.9	0.975
	Mol	2343	2008	41	15	0.352564	13.9	1
Canidae	Morph	188	2004	23	39	0.484848	14.2	0.818182
	Mol	609	2004	23	39	0.489177	14.2	1
Ceboidea	Morph	0	1993	16	12	0.542857	19.7	1
	Mol	1,800	1993	16	12	0.114286	19.7	0.866667
Chiroptera 1	Morph	207	2008	19	12	0.379085	62	1
	Mol	13,700	2005	19	12	0.196078	62	1
Chiroptera 2	Morph	239	2011	22	98	0.618182	62	1
	Mol	1140	2011	22	98	0.328571	62	1
Chrysochloridae	Morph	144	2010	18	9	0.029411	27.8	0.529412
	Mol	913	2010	18	9	0.110294	27.8	0.823529
Crocodylia	Morph	163	2004	23	28	0.25974	80	0.772727
	Mol	7,282	2011	23	28	0.207792	80	1
Cupressaceae	Morph	45	2000	39	20	0.146515	179	0.657895
	Mol	2930	2000	39	20	0.337127	179	0.947368
Didelphidae	Morph	1	2005	43	18	0.211382	34	0.761905
	Mol	4982	2005	43	18	0.101045	34	0.892857
Didelphinae	Morph	129	2011	45	25	0.194503	30	0.931818
	Mol	5,977	2011	45	25	0.172304	30	0.977273
Diprotodontia	Morph	230	2003	21	6	0.563158	49	1
	Mol	5,894	2009	21	6	0.436842	49	1
Drosophila	Morph	9	1996	9	4	0.107143	46	0.625
	Mol	1100	1996	9	4	0.214286	46	0.875
Echymyidae	Morph	62	2015	16	14	0.580952	16.3	0.933333
	Mol	5,086	2015	16	14	0.390476	16.3	0.933333
Chilabothrus/Epicrates	Morph	53	1987	10	6	0.694444	28.5	0.888889
	Mol	24	1989	10	6	0.388889	28.5	1
Erinaceidae	Morph	135	2012	22	24	0.176190	48	0.571429
	Mol	3,218	2012	22	24	0.147619	48	1
Eutheria	Morph	4541	2013	19	13	0.202614	105	1
	Mol	35,603	2013	19	13	0.20915	105	1
Feliformia	Morph	349	2005	53	85	0.10181	47	0.826923
	Mol	4,026	2005	53	85	0.11463	47	0.942308
Glires	Morph	196	2005	22	56	0.27143	82	0.952381
	Mol	5,623	2005	22	56	0.257143	82	1
Heliconius	Morph	0	1994	41	22	0.311538	11.1	0.8
	Mol	950	1981	41	22	0.144872	11.1	1
Iguanidae 1	Morph	67	2003	33	20	0.114919	108	0.875
	Mol	1200	2003	33	20	0.302419	108	1
Iguanidae 2	Morph	90	1996	13	7	0.242424	108	0.75
	Mol	959	1996	13	7	0.469697	108	1
Josiini	Morph	86	1997	22	12	0.452381	69	1
	Mol	774	1997	22	12	0.133333	69	0.738095

Supplementary Table 3: Summary metrics for the 96 phylogenetic trees included in the analysis. Clade: the phylogenetic group analysed. Data type: the type of character data used to construct the phylogeny (morphological or molecular). Phylogenetic characters: the number of phylogenetic characters used to construct the tree. Publication year: the year the source tree was published. Number of taxa: the number of terminal taxa analysed. Number of region characters: the number of characters in the biogeographic matrix. Colless's index: Colless's index of tree imbalance (higher values indicate less symmetrical, more pectinate trees). Root node age: the age of the root node for the clade in millions of years. Res: The proportion of resolved nodes within the phylogeny.

Clade	Data type	Phylogenetic Characters	Publication Year	Number of taxa	Number of region characters	Colless's Index (*C*)	Root node age (Mya)	Res
Krigia	Morph	35	1994	7	10	0.4	8.7	0.833333
	Mol	514	1994	7	10	0.333333	8.7	1
Macropodidae	Morph	83	2010	16	7	0.542857	18.8	0.933333
	Mol	43,616	2010	16	7	0.390476	18.8	1
Megachiroptera	Morph	236	2005	44	17	0.095238	35	0.813953
	Mol	3,500	2005	44	17	0.262458	35	0.976744
Mormoopidae	Morph	209	2001	15	26	0.384615	33	1
	Mol	2,538	2001	15	26	0.406593	33	1
Neckeraceae	Morph	14	2009	20	14	0.116959	32	0.368421
	Mol	242	2009	20	14	0.385965	32	1
Ophraella	Morph	88	1990	11	4	0.466667	8.8	1
	Mol	866	1995	11	4	0.444444	8.8	1
Opluridae	Morph	34	1996	10	4	0.444444	40	0.888889
	Mol	1129	1996	10	4	0.466667	40	1
Phrynosomatidae	Morph	155	1996	40	44	0.240216	77.9	0.974359
	Mol	779	1996	40	44	0.202429	77.9	0.871795
Phyllostomidae 1	Morph	220	2012	71	39	0.009937	31	1
	Mol	5,705	2012	71	39	0.189234	31	0.985714
Phyllostomidae 2	Morph	119	2002	21	28	0.178947	31	0.9
	Mol	1,362	2002	21	28	0.252632	31	0.85
Physalaemus	Morph	12	1998	10	17	0.25	39	0.777778
	Mol	1,757	1998	10	17	0.194444	39	1
Pinacea	Morph	54	2012	45	17	0.109937	160	0.953488
	Mol	686	2000	45	17	0.108879	160	0.928571
Pinales	Morph	123	1987	63	39	0.085669	313.5	0.903226
	Mol	8091	1995	63	39	0.177155	313.5	0.983871
Platynini	Morph	206	1998	23	6	0.268398	51	1
	Mol	2,516	2001	23	6	0.316017	51	0.863636
Plecotini	Morph	56	1998	10	12	0.361111	26.9	0.888889
	Mol	2,700	2001	10	12	0.277778	26.9	0.888889
Ratites	Morph	179	2012	13	7	0.393939	93	1
	Mol	15,731	2014	13	7	0.348485	93	1
Rhopalocera	Morph	99	2005	57	56	0.264935	106	0.910714
	Mol	3,159	2005	57	56	0.201948	106	0.964286
Sciuridae	Morph	9	2003	14	9	0.384615	37	0.923077
	Mol	507	2003	14	9	0.115385	37	0.615385
Sphenisciformes	Morph	159	2005	17	11	0.191667	29.5	0.9375
	Mol	2,100	2005	17	11	0.183333	29.5	0.6875
Sphenostylis	Morph	16	1994	12	7	0.309091	13.3	0.818182
	Mol	53	1994	12	7	0.436364	13.3	0.909091
Squamata	Morph	148	1988	19	10	0.20915	201	1
	Mol	33,717	2012	19	10	0.27451	201	1
Talpidae	Morph	157	2006	12	7	0.672727	61	1
	Mol	2,979	2006	12	7	0.618182	61	1

Supplementary Table 3 continued

Model	Linear Regression		
	AIC	R^2	P-value
CI ~ Type + Age + *C* + log(Regions) + log(Taxa) + S/T + Year + Res	-422.33	0.5896	$5.06x10^{-14}$
CI ~ Type + Age + *C* + log(Regions) + log(Taxa) + S/T + Res	-424.30	0.5894	$1.151 x10^{-14}$
CI ~ Type + Age + log(Regions) + log(Taxa) + S/T + Year + Res	-424.06	0.5884	$1.279 x10^{-14}$
CI ~ Type + Age + *C* + log(Regions) + log(Taxa) + S/T + Year	-423.39	0.5855	$1.722x10^{-14}$
CI ~ Type + *C* + log(Regions) + log(Taxa) + S/T + Year + Res	-421.49	0.5772	$3.978 x10^{-14}$
CI ~ Age + *C* + log(Regions) + log(Taxa) + S/T + Year + Res	-420.68	0.5736	$5.683x10^{-14}$
CI ~ Type + Age + *C* + log(Taxa) + S/T + Year + Res	-419.96	0.379	$3.257x10^{-07}$
CI ~ Type + Age + *C* + log(Regions) + log(Taxa) + Year + Res	-418.28	0.5628	$1.631x10^{-13}$
CI ~ Type + Age + *C* + log(Regions) + S/T + Year + Res	-384.57	0.3594	$3.644x10^{-07}$
CI ~ Type + Age + log(Regions) + log(Taxa) + S/T + Res	-426.02	0.379	$3.257x10^{-07}$
CI ~ Type + Age + *C* + log(Regions) + log(Taxa) + S/T	-425.35	0.5853	$3.633x10^{-15}$
CI ~ Type + *C* + log(Regions) + log(Taxa) + S/T + Res	-423.43	0.577	$8.587 x10^{-15}$
CI ~ Age + *C* + log(Regions) + log(Taxa) + S/T + Res	-422.68	0.5736	$1.207 x10^{-14}$
CI ~ Type + Age + *C* + log(Taxa) + S/T + Res	-421.91	0.5702	$1.699 x10^{-14}$
CI ~ Type + Age + *C* + log(Regions) + log(Taxa) + Res	-419.47	0.5592	$5.074 x10^{-14}$
CI ~ Type + Age + *C* + log(Regions) + S/T + Res	-385.28	0.3705	$1.774 x10^{-07}$
**CI ~ Type + Age + log(Regions) + log(Taxa) + S/T**	**-427.35***	**0.5853***	**$6.784 x10^{-16}$***
CI ~ Type + log(Regions) + log(Taxa) + S/T + Res	-425.35	0.577	$8.587 x10^{-15}$
CI ~ Age + log(Regions) + log(Taxa) + S/T + Res	-424.68	0.5736	$2.307 x10^{-15}$
CI ~ Type + Age + log(Taxa) + S/T + Res	-423.45	0.5702	$1.699 x10^{-14}$
CI ~ Type + Age + log(Regions) + log(Taxa) + Res	-421.14	0.5576	$1.163 x10^{-14}$
CI ~ Type + Age + log(Regions) + S/T + Res	-379.41	0.3168	$1.279 x10^{-06}$
CI ~ Type + log(Regions) + log(Taxa) + S/T	-426.88	1.625	$1.279 x10^{-14}$
CI ~ Age + log(Regions) + log(Taxa) + S/T	-426.56	0.5731	$4.1 x10^{-16}$
CI ~ Type + Age + log(Regions) + log(Taxa) + S/T + Year	-425.39	0.5882	$2.683 x10^{-15}$
CI ~ Type + Age + log(Regions) + log(Taxa) + S/T + *C*	-425.35	0.5853	$3.633 x10^{-15}$
CI ~ Type + Age + log(Taxa) + S/T	-424.05	0.5618	$1.321x10^{-15}$
CI ~ Type + Age + log(Regions) + log(Taxa)	-422.11	0.5529	$3.258 x10^{-15}$
CI ~ Type + Age + log(Regions) + S/T	-381.40	0.3167	$4.598 x10^{-07}$
CI ~ Type	-76.683	0.00289	0.6028
CI ~ Age	-80.375	0.04051	0.04926
CI ~ *C*	-87.843	0.1123	0.000844
CI ~ log(Regions)	-108.60	0.2849	$2.143 x10^{-08}$
CI ~ log(Taxa)	-147.70	0.5242	$< 2.2 x10^{-18}$
CI ~ S/T	-76.643	0.0025	0.6305
CI ~ Year	-82.757	0.0640	0.01287
CI ~ Res	-76.728	0.0034	0.5749

Supplementary Table 4: Results of models predicting measures of consistency index (CI), retention index (RI) and biogeographic homoplasy excess ratio (bHER) of geographic region characters as a function of whether the tree was morphological or molecular (Type), the age of the clade root (Age), Colless's index of tree balance (*C*), log of the number of regions (log(Regions)), log of the number of terminal taxa (log(Taxa)), the ratio of phylogenetic characters divided to number of taxa (S/T), phylogeny publication year (Year) and the proportion of resolved nodes (Res). Models listed are the result of stepwise regression in both directions removing and adding each variable individually at each stage and selecting the model with the lowest Akaike information criterion (AIC), as well as separate models for each variable individually. In each case the minimum adequate model (MAM) is indicated in bold with asterisks. N = 96 morphological and molecular phylogenies.

Model	Linear Regression		
	AIC	R^2	P-value
bHER ~ Type + Age + *C* + log(Regions) + log(Taxa) + S/T + Year + Res	-332.14	0.1745	0.0277
bHER ~ Type + Age + *C* + log(Regions) + S/T + Year + Res	-334.12	0.1743	0.01544
bHER ~ Type + Age + *C* + log(Regions) + log(Taxa) + Year + Res	-333.63	0.1701	0.01831
bHER ~ Type + Age + log(Regions) + log(Taxa) + S/T + Year + Res	-333.41	0.1682	0.01977
bHER ~ Type + *C* + log(Regions) + log(Taxa) + S/T + Year + Res	-332.15	0.1572	0.03048
bHER ~ Age + *C* + log(Regions) + log(Taxa) + S/T + Year + Res	-332.07	0.1565	0.03126
bHER ~ Type + Age + *C* + log(Regions) + log(Taxa) + S/T + Year	-331.95	0.1554	0.03259
bHER ~ Type + Age + *C* + log(Taxa) + S/T + Year + Res	-331.34	0.1501	0.0399
bHER ~ Type + Age + *C* + log(Regions) + log(Taxa) + S/T + Res	-327.18	0.1124	0.1481
bHER ~ Type + Age + *C* + log(Regions) + Year + Res	-335.63	0.1701	0.00944
bHER ~ Type + Age + log(Regions) + S/T + Year + Res	-335.15	0.166	0.01128
bHER ~ Type + *C* + log(Regions) + S/T + Year + Res	-334.04	0.1563	0.01699
bHER ~ Age + *C* + log(Regions) + S/T + Year + Res	-334.04	0.1562	0.01702
bHER ~ Type + Age + *C* + log(Regions) + S/T + Year	-333.95	0.1554	0.01761
bHER ~ Type + Age + *C* + S/T + Year + Res	-332.51	0.1427	0.02963
bHER ~ Type + Age + *C* + log(Regions) + S/T + Res	-328.98	0.1106	0.09941
bHER ~ Type + Age + log(Regions) + Year + Res	-336.69	0.162	0.00640
bHER ~ Age + *C* + log(Regions) + Year + Res	-335.96	0.1555	0.00855
bHER ~ Type + *C* + log(Regions) + Year + Res	-335.88	0.1548	0.00885
bHER ~ Type + Age + *C* + log(Regions) + Year	-335.60	0.1524	0.00985
bHER ~ Type + Age + *C* + Year + Res	-334.47	0.1423	0.01535
bHER ~ Type + Age + *C* + log(Regions) + Res	-330.85	0.1094	0.06
bHER ~ Age + log(Regions) + Year + Res	-337.57	0.1521	0.00435
bHER ~ Type + log(Regions) + Year + Res	-337.50	0.1515	0.00448
bHER ~ Type + Age + log(Regions) + Year + Res + S/T	-335.15	0.166	0.01128
bHER ~ Type + Age + log(Regions) + Year + Res + log(taxa)	-334.84	0.1633	0.01265
bHER ~ Type + Age + Year + Res	-333.96	0.1196	0.01955
bHER ~ Type + Age + log(Regions) + Year	-333.39	0.1143	0.02475
bHER ~ Type + Age + log(Regions) + Res	-332.08	0.1022	0.04182
**bHER ~ log(Regions) + Year + Res**	**-338.42***	**0.1419***	**0.00271***
bHER ~ Age + log(Regions) + Year + Res + *C*	-335.96	0.1555	0.00855
bHER ~ Age + log(Regions) + Year + Res + log(Taxa)	-335.69	0.1532	0.00951
bHER ~ Age + log(Regions) + Year + Res + S/T	-335.67	0.153	0.00958
bHER ~ Age + Year + Res	-334.69	0.1079	0.01434
bHER ~ Age + log(Regions) + Res	-332.84	0.0905	0.03246
bHER ~ Age + log(Regions) + Year	-332.22	0.08463	0.04246
bHER ~ log(Regions) + Year + Res + Type	-337.50	0.1515	0.00448
bHER ~ log(Regions) + Year + Res + *C*	-336.51	0.1427	0.00679
bHER ~ log(Regions) + Year + Res + S/T	-336.43	0.142	0.00704
bHER ~ log(Regions) + Year + Res + log(Taxa)	-336.42	0.1419	0.00705
bHER ~ Year + Res	-336.28	0.1041	0.00603
bHER ~ log(Regions) + Res	-334.26	0.08497	0.0161
bHER ~ log(Regions) + Year	-332.85	0.07149	0.03177
bHER ~ Type	-56.565	0.03349	0.07432
bHER ~ Age	-53.722	0.004441	0.5188
bHER ~ *C*	-56.331	0.03113	0.0855
bHER ~ log(Regions)	-54.447	0.01194	0.2893
bHER ~ log(Taxa)	-53.601	0.003187	0.5848
bHER ~ S/T	-55.166	0.0193	0.177
bHER ~ Year	-57.617	0.04403	0.04018
bHER ~ Res	-59.755	0.06509	0.01212

Supplementary Table 4 continued

Model	Linear regression		
	AIC	R^2	p-value
RI ~ Type + Age + *C* + log(Regions) + log(Taxa) + S/T + Year + Res	-320.84	0.1685	0.03462
RI ~ Type + Age + *C* + log(Regions) + log(Taxa) + S/T + Res	-322.83	0.1684	0.01959
RI ~ Type + Age + *C* + log(Regions) + log(Taxa) + Year + Res	-322.79	0.1681	0.01982
RI ~ Age + *C* + log(Regions) + log(Taxa) + S/T + Year + Res	-322.79	0.1681	0.01986
RI ~ Type + Age + *C* + log(Regions) + S/T + Year + Res	-322.77	0.1679	0.01997
RI ~ Type + Age + log(Regions) + log(Taxa) + S/T + Year + Res	-322.07	0.1619	0.02538
RI ~ Type + *C* + log(Regions) + log(Taxa) + S/T + Year + Res	-319.32	0.1374	0.06351
RI ~ Type + Age + *C* + log(Taxa) + S/T + Year + Res	-318.83	0.1331	0.07419
RI ~ Type + Age + *C* + log(Regions) + log(Taxa) + S/T + Year	-314.80	0.09586	0.2444
RI ~ Age + *C* + log(Regions) + log(Taxa) + S/T + Res	-324.77	0.1679	0.01036
RI ~ Type + Age + *C* + log(Regions) + S/T + Res	-324.77	0.1679	0.01038
RI ~ Type + Age + *C* + log(Regions) + log(Taxa) + Res	-324.75	0.1677	0.01045
RI ~ Type + Age + log(Regions) + log(Taxa) + S/T + Res	-324.07	0.1619	0.01344
RI ~ Type + *C* + log(Regions) + log(Taxa) + S/T + Res	-321.20	0.1364	0.03797
RI ~ Type + Age + *C* + log(Taxa) + S/T + Res	-320.76	0.1324	0.04431
RI ~ Type + Age + *C* + log(Regions) + log(Taxa) + S/T	-316.80	0.09586	0.1644
RI ~ Age + *C* + log(Regions) + log(Taxa) + Res	-326.72	0.1675	0.00496
RI ~ Age + *C* + log(Regions) + S/T + Res	-326.72	0.1675	0.00497
RI ~ Age + log(Regions) + log(Taxa) + S/T + Res	-326.07	0.1619	0.00643
RI ~ *C* + log(Regions) + log(Taxa) + S/T + Res	-323.00	0.1346	0.02136
RI ~ Age + *C* + log(Taxa) + S/T + Res	-322.74	0.1322	0.02365
RI ~ Age + *C* + log(Regions) + log(Taxa) + S/T	-318.12	0.0895	0.1272
RI ~ Age + *C* + log(Regions) + Res	-328.65	0.1669	0.00211
RI ~ Age + log(Regions) + log(Taxa) + Res	-327.97	0.161	0.00283
RI ~ Age + *C* + log(Regions) + log(Taxa) + Res + Year	-324.76	0.1679	0.0104
RI ~ *C* + log(Regions) + log(Taxa) + Res	-324.76	0.1324	0.01096
RI ~ Age + *C* + log(Regions) + log(Taxa) + Res + Type	-324.75	0.1677	0.01045
RI ~ Age + *C* + log(Taxa) + Res	-324.53	0.1304	0.01203
RI ~ Age + *C* + log(Regions) + log(Taxa)	-319.44	0.08301	0.09262
**RI ~ Age + log(Regions) + Res**	**-329.97***	**0.1609***	**0.00102***
RI ~ Age + *C* + log(Regions) + Res + S/T	-326.72	0.1675	0.00497
RI ~ Age + *C* + log(Regions) + Res + Year	-326.68	0.1671	0.00504
RI ~ Age + *C* + log(Regions) + Res + Type	-326.68	0.1671	0.00505
RI ~ *C* + log(Regions) + Res	-326.67	0.1316	0.00453
RI ~ Age + *C* + Res	-324.42	0.111	0.01238
RI ~ Age + *C* + log(Regions)	-321.44	0.08301	0.04569
RI ~ Age + log(Regions) + Res + S/T	-328.06	0.1618	0.00273
RI ~ Age + log(Regions) + Res + Year	-328.00	0.1612	0.00280
RI ~ Age + log(Regions) + Res + Type	-327.97	0.161	0.00283
RI ~ Age + log(Regions) + Res + log(Taxa)	-327.97	0.161	0.00283
RI ~ log(Regions) + Res	-326.73	0.1139	0.00360
RI ~ Age + Res	-326.41	0.111	0.00421
RI ~ Age + log(Regions)	-322.84	0.07721	0.02384
RI ~ Type	-43.559	0.009056	0.3564
RI ~ Age	-46.610	0.04006	0.0506
RI ~ *C*	-43.079	0.00410	0.5356
RI ~ log(Regions)	-44.999	0.02381	0.1333
RI ~ log(Taxa)	-42.876	0.00199	0.6661
RI ~ S/T	-44.432	0.01803	0.1921
RI ~ Year	-42.827	0.00148	0.7098
RI ~ Res	-50.489	0.07808	0.0058

Supplementary Table 4 continued

Clade	Morphological trees		Molecular trees	
	**Number of branches in polytomies**	**Number of polytomies**	**Number of branches in polytomies**	**Number of polytomies**
Anas	0	0	0	0
Andira	17	4	2	1
Anolis	28	11	0	0
Arctoidea	0	0	2	1
Bothropis	0	0	0	0
Canidae	5	3	0	0
Ceboidea	0	0	3	1
Chiroptera 1	0	0	0	0
Chiroptera 2	0	0	0	0
Chrysochloridae	11	3	5	1
Crocodylia	9	3	0	0
Cupressaceae	17	4	4	2
Didelphidae	16	5	17	7
Didelphinae	6	3	3	1
Diprotodontia	0	0	0	0
Drosophila	6	2	2	1
Echymyidae	3	1	3	1
Chilabothrus/Epicrates	3	1	0	0
Erinaceidae	14	4	0	0
Eutheria	0	0	0	0
Feliformia	14	4	7	3
Glires	0	0	0	0
Heliconius	0	0	13	4
Iguanidae 1	5	4	0	0
Iguanidae 2	6	2	0	0
Josiini	0	0	14	3
Krigia	3	1	0	0
Macropodidae	3	1	0	0
Megachiroptera	11	2	3	1
Mormoopidae	0	0	0	0
Neckeraceae	17	4	0	0
Ophraella	0	0	0	0
Opluridae	3	1	0	0
Phrynosomatidae	3	1	9	2
Phyllostomidae 1	0	0	0	0
Phyllostomidae 2	4	1	4	2
Physalaemus	5	2	0	0
Pinacea	5	2	7	3
Pinales	12	4	3	1
Platynini	0	0	5	1
Plecotini	3	1	3	1
Ratites	0	0	0	0
Rhopalocera	10	4	4	1
Sciuridae	3	1	9	3
Sphenisciformes	3	1	7	3
Sphenostylis	5	2	3	1
Squamata	0	0	0	0
Talpidae	0	0	0	0

Supplementary Table 5: Counts of the number of branches that are part of polytomies and number of polytomies within each group.

Clade	Type	SRL	MIG	Gmin	Gmax	GER	GERt	GER*	RCI (%)	SCI	MSM*
Arctoidea	Morph	154	62	17	135	0.619	0.635	0.826	59.740	0.400	0.274
	Mol	154	66	17	135	0.585	0.630	0.801	57.143	0.333	0.258
Chiroptera 1	Morph	96	46	10	94	0.571	0.810	0.992	52.083	0.588	0.217
	Mol	96	53	10	94	0.488	0.717	0.929	44.792	0.647	0.189
Chiroptera 2	Morph	235	118	18	161	0.301	0.455	0.565	49.787	0.545	0.153
	Mol	235	120	18	161	0.287	0.481	0.577	48.936	0.550	0.150
Chrysochloridae	Morph	58	24	6	50	0.591	0.806	0.999	58.621	0.563	0.250
	Mol	58	24	6	50	0.591	0.750	0.996	58.621	0.688	0.250
Crocodylia	Morph	138	124	21	345	0.682	0.809	0.988	10.145	0.333	0.169
	Mol	138	101	21	345	0.753	0.874	0.998	26.812	0.571	0.208
Didelphidae	Morph	220	89	11	275	0.705	1.254	0.550	59.545	0.674	0.124
	Mol	220	92	11	275	0.693	1.175	0.550	58.182	0.698	0.120
Echymyidae	Morph	31	48	10	122	0.705	1.254	0.550	59.545	0.674	0.124
	Mol	31	55	8	97	0.693	1.175	0.550	58.182	0.698	0.120
Erinaceidae	Morph	98	76	10	122	0.411	0.590	0.970	22.449	0.600	0.132
	Mol	98	42	10	122	0.714	0.946	1.000	57.143	0.700	0.238
Eutheria	Morph	295	57	12	85	0.384	0.256	0.337	80.678	0.471	0.211
	Mol	295	42	12	85	0.589	0.686	0.979	85.763	0.529	0.286
Feliformia	Morph	154	372	12	482	0.234	0.421	0.356	-141.558	0.314	0.032
	Mol	154	370	12	482	0.238	0.358	0.243	-140.260	0.329	0.032
Glires	Morph	147	84	14	161	0.524	0.598	0.829	42.857	0.500	0.167
	Mol	147	82	14	161	0.537	0.605	0.838	44.218	0.550	0.171
Iguanidae	Morph	155	209	18	439	0.546	0.477	0.594	-34.839	0.484	0.086
	Mol	155	208	18	439	0.549	0.523	0.701	-34.194	0.452	0.087
Macropodidae	Morph	47	35	6	122	0.326	0.303	0.620	25.532	0.429	0.171
	Mol	47	34	6	122	0.349	0.258	0.550	27.660	0.500	0.176
Megachiroptera	Morph	16	16	2	72	0.800	1.000	1.000	0.000	0.833	0.125
	Mol	16	22	2	72	0.714	0.792	0.995	-37.500	0.762	0.091
Mormoopidae	Morph	32	4	5	28	1.000	1.000	0.990	87.500	0.846	1.000
	Mol	32	4	5	28	1.000	1.000	0.990	87.500	0.846	1.000
Phrynosomatidae	Morph	335	53	12	145	0.692	1.097	0.708	84.179	0.842	0.226
	Mol	335	55	12	145	0.677	1.145	0.708	83.582	0.816	0.218
Phyllostomidae 1	Morph	24	66	11	207	0.719	0.534	0.613	-175.000	0.684	0.167
	Mol	24	44	11	207	0.832	0.678	0.905	-83.333	0.737	0.250
Phyllostomidae 2	Morph	68	52	4	216	0.774	0.882	0.997	23.529	0.783	0.077
	Mol	68	64	4	216	0.717	0.706	0.922	5.882	0.696	0.063
Pinales	Morph	879	814	41	1704	0.535	0.636	0.895	7.395	0.443	0.050
	Mol	879	814	41	1704	0.554	0.694	0.937	11.035	0.475	0.052
Plecotini	Morph	28	10	5	22	0.706	0.667	0.990	64.286	0.500	0.500
	Mol	28	12	5	22	0.588	0.667	0.959	57.143	0.500	0.417
Ratites	Morph	63	60	12	93	0.466	0.522	0.460	4.762	0.364	0.200
	Mol	63	53	12	93	0.342	0.276	0.760	15.873	0.455	0.226
Squamata	Morph	397	117	31	192	0.466	0.522	0.910	70.529	0.529	0.265
	Mol	397	137	31	192	0.342	0.276	0.506	65.491	0.412	0.226
Talpidae	Morph	68	56	11	64	0.151	0.053	0.087	17.647	0.200	0.196
	Mol	68	46	11	64	0.151	0.053	0.087	17.647	0.200	0.196

Supplementary Table 6: Summary metrics for the 46 phylogenetic trees included in the analysis of stratigraphic congruence. Columns are: category (morphological or molecular), standard range length (SRL), minimum implied gap (MIG), minimum possible gap (G_{min}), maximum possible gap (G_{max}), gap excess ratio (GER), topological gap excess ratio (GERt), modified gap excess ratio (GER*), relative completeness index (RCI), stratigraphic consistency index (SCI) and modified Manhattan stratigraphic measure (MSM*).

Metric	Sample size	Morphology higher	Success p-value	Molecular higher	Success p-value	Binomial p-value
GER*	23	10	0.4347826	8	0.3478261	0.21
MSM*	23	10	0.4347826	9	0.3913043	0.4049
GER	23	11	0.4782609	9	0.3913043	0.4049
GERt	23	11	0.4782609	9	0.3913043	0.4049
SCI	23	6	0.2608696	14	0.6086957	0.4049
MIG	23	10	0.4347826	11	0.4782609	0.9999
RCI	23	9	0.3913043	11	0.4782609	0.9999

Supplementary Table 7: Results of sign tests for the number of cases molecular trees are selected over morphological trees based on the following measures of stratigraphic fit: stratigraphic consistency index (SCI), minimum implied gap (MIG), relative completeness index (RCI), modified Manhattan stratigraphic measure (MSM*), gap excess ratio (GER), topological gap excess ratio (GERt), modified gap excess ratio (GER*). n = 23 biologically independent pairs of morphological and molecular phylogenies.

Metric	Wilcoxon signed-rank test statistic (W)	Z-score	Effect size (rc)	P-value
Year	2.5	1.68	0.762	0.1148
Size	276	4.2	1	2.384×10^{-07}
S/T	276	4.2	1	2.384×10^{-07}
Res	99	2.22	0.65	0.02877
C	105	-1.00	-0.239	0.3294
CI	181	1.77	0.431	0.07958
RI	176	1.61	0.391	0.176
CI & RI p-value	72	-1.51	-0.377	0.135
bHER	218	2.43	0.58	0.01353
MIG	88	-0.262	-0.0667	0.8079
RCI	110	0.187	0.0476	0.8666
SCI	140.5	1.33	0.338	0.1913
MSM*	92	-0.121	-0.0316	0.9198
GER	91	-0.523	-0.133	0.6142
GERt	87	-0.672	-0.171	0.5135
GER*	90	0.196	0.0526	0.8617

Supplementary Table 8: Results of paired Wilcoxon signed-rank tests testing morphological and molecular trees used in the stratigraphic congruence analysis study. Tests were carried out for the following metrics: publication year (Year), number of phylogenetic characters (Size), Colless's index of tree balance (*C*), consistency index (CI), retention index (RI), probability of CI & RI values falling within the null distribution (CI & RI p-value), biogeographic homoplasy excess ratio (bHER), minimum implied gap (MIG), relative completeness index (RCI) stratigraphic consistency index (SCI), modified Manhattan stratigraphic measure (MSM*), gap excess ratio (GER), topological gap excess ratio (GERt) and modified gap excess ratio (GER*) Statistically significant results are highlighted in green. n = 23 biologically independent pairs of morphological and molecular phylogenies.

Dataset	Metric	Sample size (n)	Morphology higher	Success p-value	Molecular higher	Success p-value	Binomial p-value
Whole dataset	CI	23	8	0.364	14	0.609	0.405
	RI	23	7	0.318	15	0.652	0.21
	CI & RI p-value	23	11	0.5	10	0.434	0.678
	bHER	23	6	0.273	17	0.739	0.035
All cases with a difference	CI	22	8	0.381	14	0.636	0.286
	RI	22	7	0.333	15	0.682	0.134
	CI & RI p-value	21	11	0.550	10	0.476	0.999
	bHER	23	6	0.273	17	0.739	0.035
Either fit significantly different from random	CI	17	6	0.375	10	0.588	0.629
	RI	17	5	0.313	11	0.647	0.332
	CI & RI p-value	16	6	0.4	8	0.5	0.999
Either fit significantly different from random & one fit better than the other	CI	16	6	0.4	10	0.625	0.455
	RI	16	5	0.333	11	0.688	0.210
	CI & RI p-value	14	6	0.462	8	0.571	0.790

Supplementary Table 9: Results of sign tests for the number of cases molecular trees are selected over morphological trees used in the stratigraphic congruence analysis study, based on the following measures of biogeographic fit: consistency index (CI), retention index (RI), CI & RI randomisation p-values (CI & RI p-value) and biogeographic homoplasy excess ratio (bHER). Tests were carried out on the whole dataset, only those datasets where there was a difference in fit values, only those datasets in which at least one CI or RI values significantly differed from a distribution of 10,000 randomisations and only those datasets in which at least one of the CI or RI values significantly differed from a distribution of 10,000 randomisations and there was a difference in fit value.

Metric	Data partition	Sample size (N)	Spearman's rho value (R_S)	P-value	Pearson's *r*	P-value
Taxa	All	96	0.284	0.005	0.180	0.088
	Morphological	48	0.308	0.033	0.177	0.229
	Molecular	48	0.258	0.076	0.097	0.510
Regions	All	96	0.238	0.019	0.270	0.010
	Morphological	48	0.278	0.056	0.243	0.096
	Molecular	48	0.200	0.172	0.193	0.188
C	All	96	-0.080	0.438	-0.082	0.424
	Morphological	48	-0.105	0.479	-0.089	0.549
	Molecular	48	-0.042	0.775	-0.073	0.623
CI	All	96	-0.239	0.019	-0.267	0.011
	Morphological	48	-0.245	0.093	-0.246	0.092
	Molecular	48	-0.228	0.120	-0.266	0.068
RI	All	96	0.038	0.715	-0.099	0.351
	Morphological	48	0.101	0.494	0.009	0.953
	Molecular	48	-0.032	0.828	-0.090	0.544
CI & RI p-value	All	96	-0.284	0.005	-0.237	0.024
	Morphological	48	-0.281	0.053	-0.193	0.189
	Molecular	48	-0.274	0.060	-0.258	0.077
bHER	All	96	0.257	0.012	0.181	0.087
	Morphological	48	0.292	0.044	0.316	0.029
	Molecular	48	0.184	0.210	0.112	0.449

Supplementary Table 10: Results of tests for correlation between the publication year and the following metrics: number of taxa (Taxa), number of region characters (Regions), Colless's Index of tree balance (*C*), consistency index (CI), retention index (RI), probability of CI & RI values falling within the null distribution (CI & RI p-value) and the biogeographic homoplasy excess ratio (bHER). Spearman's rank-order correlations were calculated for the whole dataset, as well as for a subset of the data in which outlying high values were removed before calculating correlation coefficients. Pearson correlation coefficients were only calculated for the dataset with outliers (> 9,000 characters) removed. Statistically significant results are highlighted in green.

Clade	Author	Category	Data Type
Anas	Omland 1994[31]	Morphological	Parsimony 34 characters, adult plumage, natal plumage, soft part, trachea, skeleton
		Molecular	Parsimony, mtDNA 119 characters
Andira	Pennington 1996[32]	Morphological	Parsimony, 10 characters, 1 growth habit, 1 seedling, 2 vegetative, 4 floral, 2 fruit
		Molecular	Parsimony, 38 restriction site characters (cpDNA)
Anolis	Jackman *et al.* 1999[33]	Morphological	Parsimony, 16 characters, 8 craniomandibular, 8 postcranial
		Molecular	Parsimony, mtDNA 1,455bp, ND2 gene, tRNA
Arctoidea	Finarelli 2008[34]	Morphological	Parsimony, 80 characters, 35 cranial, 45 dental
	Flynn *et al.* 2005[35]	Molecular	Parsimony, DNA 6243bp, mitochondrial 3266bp (CYTB 1149bp, 12S 1067, ND2 1050), nuclear 2977 (TR-i-1 1491bp, IRBP 1043bp, TBG 443bp)
Bothropis	Fenwick *et al.* 2009[36]	Morphological	Parsimony, 92 characters, 38 scale, 18 external soft parts, 6 male genitalia, 2 vertebral, 28 craniomandibular
		Molecular	Maximum likelihood, 2343bp DNA, 12S rRNA, 16S rRNA, ND4, cyt b
Canidae	Zrzavý & Řičánková 2004[37]	Morphological	Parsimony, 188 characters, 29 craniomandibular, 36 dental, 14 postcranial, 36 soft part, 9 developmental, 48 behavioural, 14 chromosomal
		Molecular	Parsimony,235 characters CYTB, 180 characters COI,194 characters COII
Ceboidea	Kay 1990[38]	Morphological	Biosystematic consensus, dental characters
	Schneider *et al.* 1993[39]	Molecular	Parsimony, DNA 1,800bp e-globin gene
Chiroptera 1	Simmons *et al.* 2008[40]	Morphological	Parsimony, 207 characters, 8 dentary, 15 craniomandibular, 10 inner ear, 78 postcranial, 93 soft part
	Teeling *et al.* 2005[41]	Molecular	Maximum likelihood, 17 nuclear genes 13,700bp
Chiroptera 2	Fracasso *et al.* 2011[42]	Morphological	Parsimony, 239 characters, 48 dental, 93 soft part, 80 postcranial, 18 craniomandibular
	Agnarsson *et al.* 2011[43]	Molecular	Bayesian, 1140bp CYTB
Chrysochloridae	Asher *et al.* 2010[44]	Morphological	Parsimony, 144 characters, 45 postcranial, 37 dentition & mandible, 62 cranium
		Molecular	Parsimony, 913bp nuclear GHR gene
Crocodylia	Gatesy *et al.* 2004[45]	Morphological	Parsimony, 163 characters, 34 postcranial, 6 osteoderm, 124 craniomandibular
	Oaks *et al.* 2011[46]	Molecular	Bayesian, DNA 7,282bp, 4 mtDNA, 9 nuclear
Cupressaceae	Gadek *et al.* 2000[47]	Morphological	Parsimony, 45 characters, 3 growth, 8 stem and wood, 16 leaves, 2 pollen, 5 megagametophyte and archegonia, 9 embryonic & ovular, 1 female cone, 1 chromosomal
		Molecular	Parsimony, DNA 2930bp, matK 1530bp, rbcL 1400bp
Didelphidae	Jansa *et al.* 2005[48]	Morphological	Parsimony, 1 character dorsal pelage pattern
		Molecular	Maximum likelihood, DNA 4982bp, mtDNA, cytB gene 1149bp, 4 nuclear gene, BRCA1 946bp, IRBP 1158bp, SLC38 884bp, OGT 653bp
Didelphinae	Oliveira *et al.* 2011[49]	Morphological	Parsimony, 129 characters, 39 soft part, 49 craniomandibular, 45 dentary, 4 karyological
	Voss & Jansa 2009[50]	Molecular	Maximum Likelihood, 5 nuclear genes 5977bp, 2,100bp BRCA1, 1,000bp vWF, 1158bp IRBP, 1176 DMP1, 543bp RAG1
Diprotodontia	Horovitz *et al.* 2003[51]	Morphological	Parsimony, 230 characters, 149 postcranial, 26 dental, 50 cranial, 5 soft part
	Meredith *et al.* 2009[52]	Molecular	Maximum likelihood, DNA 5894bp, ApoB, BRCA1, IRBP, Rag1, vWF
Drosophila	Piano *et al.* 1997[53]	Morphological	Parsimony, 9 characters chorion ultrastructure
		Molecular	Parsimony, Yp1 gene 1,100bp
Echymyidae	Olivares & Verzi 2015[54]	Morphological	Parsimony, 62 characters, 15 dentary, 47 craniomandibular
		Molecular	Parsimony,5086bp DNA, 2 mitochondrial genes (1140bp CYTB, 932bp 12S rRNA), 3 nuclear exons (801bp growth hormone receptor exon 10, 1149bp vWF, 1064bp RAG1)

Supplementary Table 11: Source papers and summary of the methods and characters used to infer molecular and morphological phylogenies for the 48 clades in this study.

Clade	Author	Category	Data Type
Chilabothrus/Epicrates	Kluge 1989[55]	Morphological	Parsimony, 53 characters, 8 external soft parts, 39 craniomandibular, 6 postcranial
	Tolson 1987[56]	Molecular	Parsimony, Skin & scent gland lipids, 24 characters
Erinaceidae	He *et al.* 2012[57]	Morphological	Parsimony, 135 characters, 61 cranial, 59 dentary, 6 postcranial, 9 pelage
		Molecular	Bayesian, mtDNA 3,218bp, 982bp 12S rRNA, 1,140bp CYTB, 1,047bp ND2
Eutheria	O'Leary *et al.* 2013[58]	Morphological	Parsimony, 4541 characters
		Molecular	Parsimony, 35,603bp, 27 nuclear genes
Feliformia	Gaubert *et al.* 2005[59]	Morphological	Parsimony, 349 characters, 99 craniomandibular, 62 external soft parts, 57 internal soft parts, 74 dentary, 57 postcranial
		Molecular	Biosystematic consensus, DNA 4026bp, 2 nuclear genes (897bp transthyretin intron I, 945bp IRBP) 2 mitochondrial genes (1,140bp CYTB, 1,044bp ND2)
Glires	Asher *et al.* 2005[60]	Morphological	Parsimony, 196 characters, 79 dentary, 73 craniomandibular, 19 inner ear, 54 postcranial, 4 soft part
		Molecular	Parsimony,5623bp, mtDNA (1146bp CYTB), nuclear genes (1131bp A2AB, 1227bp IRBP, 1233bp vWF, 886bp GHR)
Heliconius	Brown Jr. 1981[61]	Morphological	Biosystematic consensus, egg, larva, pupa, imago, behavioural, biogeographic, karyological
	Brower 1994[62]	Molecular	Parsimony, mtDNA fragment 950bp, 3 genes
Iguanidae 1	Schulte *et al.* 2003[63]	Morphological	Parsimony, 67 characters, 28 craniomandibular, 12 postcranial, 26 soft part
		Molecular	Parsimony, mtDNA 1200bp, ND1&2 876bp, tRNA 324bp
Iguanidae 2	Sites *et al.* 1996[64]	Morphological	Parsimony, 90 characters, 47 craniomandibular, 22 postcranial, 21 soft part
		Molecular	Parsimony, mtDNA 959bp, ND4 gene 742bp, tRNAs 217bp
Josiini	Miller 1996[65]	Morphological	Parsimony, 86 characters, 59 adult, 27 larval & pupal
	Miller *et al.* 1997[66]	Molecular	Parsimony, DNA 774bp, rDNA (313bp 28S, 202bp 18S), mtDNA (461bp COII)
Krigia	Kim & Jansen 1994[67]	Morphological	Parsimony, 35 characters, growth, leaves, pollen, chromosomal
		Molecular	Parsimony, 514bp, rDNA ITS region 262bp, cpDNA 252bp
Macropodidae	Prideaux & Warburton 2010[68]	Morphological	Parsimony, 83 characters, 48 craniodental, 35 postcranial
	Mitchell *et al.* 2014[69]	Molecular	Maximum likelihood, DNA 43,616bp ,101 mitochondrial genes, 26 nuclear genes
Megachiroptera	Giannini & Simmons 2005[70]	Morphological	Parsimony 236 characters, Hard part (108 craniomandibular & 64 postcranial), 62 soft part (external & internal), 2 behavioural
		Molecular	Parsimony (direct optimization) 4 mitochondrial genes, 1 nuclear gene 3,500bp
Mormoopidae	Simmons & Conway 2001[71]	Morphological	Parsimony, 209 characters, hard parts (47 craniodental & 60 postcranial), 102 soft parts (external & internal organs)
	Lewis-Oritt *et al.* 2001[72]	Molecular	Maximum likelihood, 2,538bp, 1 mitochondrial gene 1,140bp, 1 nuclear gene 1,398bp
Neckeraceae	Sotiaux *et al.* 2009[73]	Morphological	Parsimony, 14 characters, leaves
		Molecular	Bayesian, nuclear rDNA 242bp, 5.8S gene, rpl16 group II intron, rps4-trnT-trnL-trnF
Ophraella	Futuyma & McCafferty 1990[74]	Morphological	Parsimony, 88 characters, 50 imago, 3 egg, 27 larva, 6 pupa
	Funk *et al.* 1995[75]	Molecular	Parsimony, 866bp, 1 rRNA, 1 mitochondrial gene
Opluridae	Titus & Frost 1996[76]	Morphological	Parsimony, 34 characters, 10 craniomandibular, 7 postcranial, 17 soft part
		Molecular	Parsimony, mtDNA 1129bp, 12S rDNA, valine tDNA, 16S rDNA
Phyllostomidae 1	Dávalos et al. 2012[77]	Morphological	Parsimony, 220 characters, hard part (craniomandibular & postcranial), soft part (external & internal), karyological
		Molecular	Maximum likelihood,5,705bp, CytB 1,140bp, 12S, tRNA-Val & 16S 2608bp, COX1 657bp, RAG2 nuclear fragment 1,300bp

Supplementary Table 11 continued

Clade	Author	Category	Data Type
Phyllostomidae 2	Carstens *et al.* 2002[78]	Morphological	Parsimony, 119 characters,16 craniomandibular, 43 dentary, 54 internal soft parts, 3 postcranial, 3 skin
		Molecular	Maximum likelihood DNA 1362bp (RAG-2 gene)
Phrynosomatidae	Reeder & Wiens 1996[79]	Morphological	Parsimony, 155 characters, 60 scalation, 55 osteology, 15 colouration, 9 behaviour, 9 myology, 4 karyology, 2 protein electrophoresis, 1 life history
		Molecular	Parsimony, mtDNA 779bp, 12S rRNA gene 253bp, 16S rRNA gene 429bp
Physalaemus species group	Cannatella *et al.* 1998[80]	Morphological	Parsimony, 12 characters, 5 craniomandibular, 2 postcranial, 5 soft part
		Molecular	Maximum likelihood, 1,757bp, 12S 1214bp, COI 543bp
Pinacea	Klymiuk & Stockey 2012[81]	Morphological	Parsimony, 54 characters, 23 bract, 17 ovuliferous scale, 8 seed structure, 6 seed position and arrangement
	Wang *et al.* 2000[82]	Molecular	Parsimony, 686bp, Chloroplast gene (545bp matK), mitochondrial gene (141bp nad5)
Pinales	Hart 1987[83]	Morphological	Parsimony 123 characters, 3 growth, 23 stem and wood anatomy, 16 leaf, 5 chemistry, 1 sex distribution, 7 microsporangiate strobilus, 15 microgametophyte, 27 embryo, 16 ovulate strobilus, 9 ovule and seeds, 1 cytology
	Tsumura *et al.* 1995[84]	Molecular	Parsimony, 6 chloroplast genes 8091bp, frxC 779bp, rbcL 1387bp, psbA 939bp, psbD 1042bp, trnK 2569bp, 16S 1375bp
Platynini	Liebherr & Zimmerman 1998[85]	Morphological	Parsimony, 206 characters, 44 female reproductive tract, 23 male genitalia, 139 external
	Cryan *et al*. 2001[86]	Molecular	Parsimony, mtDNA & nuclear 2516bp, cytochrome oxidase II 624bp, cytochrome b 783bp, 28S rDNA 668bp, wingless 441bp
Plecotini	Bogdanowicz *et al.* 1998[87]	Morphological	Parsimony 56 characters, 37 hard part (craniomandibular), 8 soft part (external), 11 karyological
	Hoofer & Bussche 2001[88]	Molecular	Parsimony 3 mitochondrial genes 2,700bp
Ratites	Worthy & Scofield 2012[89]	Morphological	Parsimony 179 characters, 63 craniomandibular, 116 post cranial
	Mitchell *et al.* 2014[90]	Molecular	Parsimony, mitochondrial genome 15,731bp
Rhopalocera	Wahlberg *et al.* 2005[91]	Morphological	Parsimony, 99 characters, 39 wing venation, 19 leg, 14 head, 21 thoracic, 2 abdominal
		Molecular	Bayesian,3159bp, COI 1531bp, EF-1a 1225bp, wingless 403bp
Sciuridae	Cardini 2003[92]	Morphological	UPGMA dendrogram, 9 landmarks
	Steppan *et al.* 1999[93]	Molecular	Maximum likelihood, cytB gene 507bp
Sphenisciformes	Bertelli & Giannini 2005[94]	Morphological	Parsimony, 159 characters, 66 integument, 70 osteology, 15 myology, 7 breeding behaviour, 1 digestive tract
		Molecular	Parsimony, mtDNA 2,100bp, 12S rDNA 958bp, cytB 1142bp
Sphenostylis	Potter & Doyle 1994[95]	Morphological	Parsimony, 16 characters, 1 leaf, 4 inflorescence, 4 petals, 5 stamen & anther, 2 seed
		Molecular	Parsimony, cpDNA 53 mutation characters
Squamata	Estes *et al.* 1988[96]	Morphological	Parsimony, 148 characters, 88 craniomandibular, 42 postcranial, 17 soft part, 1 developmental
	Wiens *et al.* 2012[97]	Molecular	Maximum likelihood, DNA 33,717bp, 44 nuclear genes
Talpidae	Sánchez-Villagra 2006[98]	Morphological	Parsimony, 157 characters, 47 dental, 25 cranial, 80 postcranial, 3 soft part
	Shinohara *et al.* 2004[99]	Molecular	Parsimony, 2979bp, 1,140bp CYTB, 829bp 12S rRNA, 1,010bp RAG-1

Supplementary Table 11 continued

Clade	Type	CI	RI	CI & RI p-value	bHER
Anas	Morph	0.40625	0.309091	0.09609	0.12881503
	Mol	0.440678	0.4	0.014499	0.24851405
Andira	Morph	0.375	0.090909	0.026497	0.08122014
	Mol	0.4	0.181818	0.031697	0.13644214
Anolis	Morph	0.23913	0.102564	0.008899	0.06964875
	Mol	0.323529	0.410256	0.0001	0.36348654
Arctoidea	Morph	0.232653	0.173815	0.80482	-0.072929
	Mol	0.230181	0.162517	0.128687	0.08844907
Bothropis	Morph	0.174419	0.236559	0.010999	0.11361684
	Mol	0.178571	0.258065	0.0038	0.14092556
Canidae	Morph	0.33913	0.146067	0.024698	0.10101089
	Mol	0.325	0.089888	0.090691	0.06543848
Ceboidea	Morph	0.27907	0.261905	0.166983	0.0859906
	Mol	0.25	0.142857	0.558044	-0.0374401
Chiroptera 1	Morph	0.3158	0.6667	0.0001	0.46232598
	Mol	0.2791	0.6026	0.0001	0.46284477
Chiroptera 2	Morph	0.189043	0.176704	0.0002	0.33230828
	Mol	0.189042	0.176875	0.0043	0.204287
Chrysochloridae	Morph	0.298688	0.078498	0.275472	0.00919093
	Mol	0.302866	0.096061	0.072993	0.08613425
Crocodylia	Morph	0.405797	0.254545	0.0007	0.23064943
	Mol	0.41791	0.290909	0.001	0.23431224
Cupressaceae	Morph	0.294118	0.076923	0.065893	0.04394849
	Mol	0.30303	0.115385	0.026397	0.07553112
Didelphidae	Morph	0.104651	0.129944	0.339366	0.0106166
	Mol	0.113924	0.20904	0.053395	0.10720812
Didelphinae	Morph	0.12561	0.136958	0.012899	0.11091583
	Mol	0.126972	0.14747	0.012199	0.11156585
Diprotodontia	Morph	0.428571	0.578947	0.001	0.49023806
	Mol	0.545455	0.736842	0.0005	0.67972738
Drosophila	Morph	0.444444	0	0.310169	-0.076519
	Mol	0.5	0.2	0.106789	0.0990991
Echymyidae	Morph	0.268208	0.164104	0.568143	-0.0402742
	Mol	0.268953	0.167063	0.19978	0.06042756
Chilabothrus/Epicrates	Morph	0.3636	0	0.481	-0.102
	Mol	0.571	0.571	0.002	0.517
Erinaceidae	Morph	0.391801	0.019175	0.0003	0.14142363
	Mol	0.402674	0.061739	0.0024	0.15856552
Eutheria	Morph	0.1912	0.2667	0.284272	0.05252622
	Mol	0.26	0.5067	0.002	0.36167166
Feliformia	Morph	0.109836	0.092048	0.0033	0.13918495
	Mol	0.111745	0.109266	0.0004	0.19197351
Glires	Morph	0.237389	0.119981	0.014199	0.15190304
	Mol	0.23737	0.119659	0.017698	0.15221366
Heliconius	Morph	0.1128	0.2575	0.120788	0.05555107
	Mol	0.108374	0.223176	0.205379	0.03648322
Iguanidae 1	Morph	0.30303	0.432099	0.0001	0.35777601
	Mol	0.31746	0.469136	0.001	0.39045913
Iguanidae 2	Morph	0.4375	0.250	0.039096	0.15275777
	Mol	0.466667	0.333333	0.016498	0.23018447
Josiini	Morph	0.226415	0.145833	0.450555	-0.0076161
	Mol	0.214286	0.083333	0.872213	-0.0398582

Supplementary Table 12: Summary of fit metrics for the 96 phylogenetic trees included in the analysis. Type (morphological or molecular phylogeny), consistency index (CI), retention index (RI), probability of CI & RI values falling within the null distribution (CI & RI p-value) and biogeographic homoplasy excess ratio (bHER).

Clade	Type	CI	RI	CI & RI p-value	bHER
Krigia	Morph	0.434783	0.133333	0.334667	-0.0061686
	Mol	0.47619	0.266667	0.224778	0.08991627
Macropodidae	Morph	0.212302	0.206428	0.021998	0.19808151
	Mol	0.213773	0.212985	0.028797	0.19139957
Megachiroptera	Morph	0.207317	0.22619	0.0007	0.15873614
	Mol	0.223684	0.297619	0.001	0.22455964
Mormoopidae	Morph	0.273684	0.316832	0.09989	0.12910787
	Mol	0.270833	0.306931	0.124388	0.11753657
Neckeraceae	Morph	0.4375	0.217391	0.516548	-0.0353971
	Mol	0.378378	0	0.013199	0.14045861
Ophraella	Morph	0.363636	0	0.79752	-0.2281779
	Mol	0.363636	0	0.80062	-0.2230279
Opluridae	Morph	0.571429	0.625	0.0035	0.52680642
	Mol	0.571429	0.625	0.005599	0.51799486
Phrynosomatidae	Morph	0.184874	0.208163	0.013099	0.09724488
	Mol	0.176707	0.163265	0.114389	0.04677011
Phyllostomidae 1	Morph	0.091335	0.333333	0.0006	0.14220533
	Mol	0.098985	0.390034	0.0001	0.22209001
Phyllostomidae 2	Morph	0.202952	0.187454	0.025297	0.15841902
	Mol	0.201217	0.178712	0.012799	0.18528281
Physalaemus	Morph	0.68	0.2	0.077592	0.10472482
	Mol	0.708333	0.3	0.041296	0.17009497
Pinacea	Morph	0.234043	0.370968	0.001	0.33296894
	Mol	0.22	0.419355	0.001	0.2790861
Pinales	Morph	0.161157	0.8607	0.0003	0.14857743
	Mol	0.152941	0.149606	0.005999	0.09134048
Platynini	Morph	0.222222	0.086957	0.348765	-0.0046309
	Mol	0.25	0.217391	0.010399	0.15442541
Plecotini	Morph	0.5	0.333333	0.019498	0.23859801
	Mol	0.521739	0.388889	0.010799	0.28356031
Ratites	Morph	0.538462	0.538462	0.0009	0.48014591
	Mol	0.538462	0.538462	0.001999	0.47920735
Rhopalocera	Morph	0.089314	0.124233	0.210179	0.02828491
	Mol	0.08903	0.121166	0.486551	-0.0022117
Sciuridae	Morph	0.428571	0.4	0.0006	0.32919671
	Mol	0.391304	0.3	0.0023	0.24116362
Sphenisciformes	Morph	0.268293	0.166667	0.267073	0.03598352
	Mol	0.275	0.194444	0.140286	0.08450638
Sphenostylis	Morph	0.368421	0.076923	0.337066	-0.0145075
	Mol	0.368421	0.076923	0.476052	-0.0488044
Squamata	Morph	0.153846	0.179104	0.741326	-0.0753655
	Mol	0.16129	0.223881	0.49705	-0.0150679
Talpidae	Morph	0.401045	0.120195	0.024698	0.24225203
	Mol	0.403791	0.129329	0.0002	0.52143951

Supplementary Table 12 continued

Metric	Data partition	Sample size (N)	Shapiro-Wilk W value	P-value	Normally distributed
Size	All	96	0.953	0.002	No
	Morphological	48	0.223	3.222×10^{-14}	No
	Molecular	48	0.536	4.33×10^{-11}	No
Taxa	All	96	0.849	2.331×10^{-8}	No
Regions	All	48	0.752	2.689×10^{-11}	No
Year	All	96	0.968	0.018	No
	Morphological	48	0.962	0.122	Yes
	Molecular	48	0.965	0.160	Yes
C	All	96	0.932	0.008	No
	Morphological	48	0.957	0.076	Yes
	Molecular	48	0.939	0.014	No
Res	All	96	0.707	1.506×10^{-12}	No
	Morphological	48	0.780	4.604×10^{-7}	No
	Molecular	48	0.694	1.058×10^{-8}	No
CI	All	96	0.927	5.697×10^{-5}	No
	Morphological	48	0.960	0.102×10^{-8}	No
	Molecular	48	0.898	0.001	No
RI	All	96	0.872	1.649×10^{-7}	No
	Morphological	48	0.868	6.851×10^{-5}	No
	Molecular	48	0.875	0.001	No
CI & RI p-value	All	96	0.676	4.265×10^{-13}	No
	Morphological	48	0.743	8.167×10^{-8}	No
	Molecular	48	0.600	3.278×10^{-10}	No
bHER	All	96	0.945	0.001	No
	Morphological	48	0.743	8.167×10^{-8}	No
	Molecular	48	0.600	3.278×10^{-10}	No

Supplementary Table 13: results of Shapiro-Wilks tests for normality on metrics of interest: number of phylogenetic characters (Size), number of terminal taxa (Taxa), number of region characters (Regions), year in which the source paper for the tree was published (Year), Colless's index of tree balance (*C*), the proportion of resolved nodes (Res), consistency index (CI), retention index (RI), probability of CI & RI values falling within the null distribution (CI & RI p-value) and the biogeographic homoplasy excess ratio (bHER).

Metric	Data partition	Sample size (N)	Taxa		Regions:Taxa		Phylogenetic characters		Publication year	
			BP	P-value	BP	P-value	BP	P-value	BP	P-value
Taxa	All	96	NA	NA	2.452	0.117	0.367	0.545	0.125	0.724
	Morphological	48	NA	NA	1.226	0.268	0.273	0.601	0.066	0.797
	Molecular	48	NA	NA	1.226	0.268	0.396	0.529	0.067	0.796
Regions	All	96	0.105	0.746	4.429	0.035	0.306	0.580	5.234	0.022
	Morphological	48	0.053	0.819	2.215	0.137	< 0.001	0.992	2.867	0.090
	Molecular	48	1.028	0.311	2.215	0.137	0.372	0.542	2.399	0.121
Regions:Taxa	All	96	1.661	0.197	NA	NA	0.245	0.621	2.654	0.103
	Morphological	48	0.831	0.362	NA	NA	0.064	0.801	1.759	0.185
	Molecular	48	0.831	0.362	NA	NA	0.242	0.623	0.985	0.321
Size	All	96	0.800	0.371	0.771	0.380	NA	NA	5.510	0.019
	Morphological	48	0.166	0.684	0.113	0.737	NA	NA	2.427	0.119
	Molecular	48	1.028	0.311	0.826	0.364	NA	NA	4.672	0.031
Year	All	96	0.014	0.905	1.528	0.216	1.220	0.269	NA	NA
	Morphological	48	0.114	0.736	1.715	0.190	0.805	0.370	NA	NA
	Molecular	48	0.006	0.937	0.289	0.591	0.552	0.457	NA	NA
C	All	96	6.523	0.011	2.062	0.151	2.264	0.132	0.919	0.338
	Morphological	48	4.793	0.029	1.216	0.270	0.538	0.463	0.052	0.819
	Molecular	48	3.707	0.054	2.005	0.157	1.295	0.255	3.040	0.081
CI	All	96	2.982	0.084	0.261	0.609	0.483	0.487	6.873	0.009
	Morphological	48	3.552	0.059	0.085	0.771	0.707	0.401	2.074	0.150
	Molecular	48	1.714	0.191	0.195	0.659	0.647	0.421	5.682	0.017
RI	All	96	0.216	0.642	2.920	0.088	0.039	0.844	4.073	0.044
	Morphological	48	0.993	0.319	1.826	0.177	0.156	0.693	2.944	0.086
	Molecular	48	1.826	0.177	1.216	0.270	0.101	0.750	1.380	0.240
CI & RI p-value	All	96	2.781	0.095	0.118	0.732	0.130	0.719	1.494	0.222
	Morphological	48	2.931	0.087	1.726	0.189	0.294	0.588	0.106	0.745
	Molecular	48	0.527	0.468	1.219	0.270	0.068	0.794	2.060	0.151
bHER	All	96	7.140	0.008	3.456	0.063	0.023	0.878	1.196	0.274
	Morphological	48	4.698	0.030	0.885	0.347	0.337	0.561	0.285	0.593
	Molecular	48	3.271	0.071	2.676	0.102	0.036	0.850	1.937	0.164

Supplementary Table 14: Results of Breusch-Pagan tests for heteroskedasticity on metrics of interest: number of terminal taxa (Taxa), number of region characters (Regions), the ratio of regions to terminal taxa (Regions:Taxa), number of phylogenetic characters (Size), year in which the source paper for the tree was published (Year), Colless's index of tree balance (*C*), consistency index (CI), retention index (RI), probability of CI & RI values falling within the null distribution (CI & RI p-value) and the biogeographic homoplasy excess ratio (bHER). Statistically significant results are highlighted in green.

Metric	Data partition	Spearman's rho (R_S)	P-value	Spearman's rho (R_S), no outliers	P-value	Pearson's *r*, no outliers	P-value
Taxa	All	0.200	0.051	0.260	0.013	0.247	0.018
	Morphological	0.306	0.034	0.369	0.013	0.270	0.073
	Molecular	0.280	0.054	0.438	0.003	0.432	0.004
Regions	All	0.173	0.092	0.256	0.014	0.201	0.057
	Morphological	0.277	0.057	0.328	0.028	0.457	0.002
	Molecular	0.215	0.142	0.407	0.007	0.336	0.028
Year	All	0.406	<0.001	0.338	0.001	0.302	0.004
	Morphological	0.435	0.002	0.344	0.021	0.303	0.043
	Molecular	0.663	<0.001	0.583	<0.001	0.516	<0.001
C	All	-0.081	0.435	-0.096	0.365	-0.121	0.252
	Morphological	0.006	0.968	0.096	0.530	0.056	0.714
	Molecular	-0.109	0.459	-0.159	0.309	-0.178	0.254
Res	All	0.380	<0.001	0.348	0.001	0.188	0.066
	Morphological	0.443	0.002	0.369	0.015	0.329	0.031
	Molecular	0.114	0.441	0.017	0.915	0.191	0.220
CI	All	-0.212	0.038	-0.220	0.036	-0.183	0.083
	Morphological	-0.319	0.027	-0.384	0.009	-0.389	0.008
	Molecular	-0.329	0.022	-0.354	0.020	-0.332	0.030
RI	All	0.144	0.160	0.071	0.503	0.007	0.945
	Morphological	0.124	0.401	0.172	0.259	0.131	0.390
	Molecular	0.133	0.369	-0.025	0.871	-0.053	0.736
CI & RI p-value	All	-0.232	0.023	-0.232	0.027	-0.167	0.114
	Morphological	-0.325	0.024	-0.358	0.016	-0.254	0.093
	Molecular	-0.208	0.156	-0.211	0.173	-0.220	0.156
bHER	All	0.263	0.010	0.230	0.028	0.106	0.319
	Morphological	0.321	0.026	0.343	0.021	0.274	0.069
	Molecular	0.159	0.280	0.081	0.605	0.039	0.804

Supplementary Table 15: Results of tests for correlation between number of phylogenetic characters and the following metrics: number of taxa (Taxa), number of region characters (Regions), year in which the source paper for the tree was published (Year), Colless's index of tree balance (*C*), consistency index (CI), retention index (RI), probability of CI & RI values falling within the null distribution (CI & RI p-value) and the biogeographic homoplasy excess ratio (bHER). Spearman's rank-order correlations were calculated for the whole dataset, as well as for a subset of the data in which outlying high values were removed before calculating correlation coefficients. Pearson correlation coefficients were only calculated for the dataset with outliers removed. Statistically significant results are highlighted in green. N = 96 morphological and molecular phylogenies (all data), n = 90 morphological and molecular phylogenies (no outliers).

Metric	Data partition	Spearman's rho (R_S)	P-value	Pearson's *r*	P-value
Regions	All	0.6097748	$4.214x10^{6}$	0.4111228	0.003698
Regions: Taxa	All	-0.1505231	0.3072	-0.1690282	0.2508
Size	All	0.2150108	0.03742	-0.03565	0.733
	Morphological	0.3486572	0.01756	-0.0249569	0.8692
	Molecular	0.2802186	0.05372	-0.0542908	0.714
Year	All	0.2840222	0.005041	0.13714	0.1827
	Morphological	0.3082583	0.03304	0.1771175	0.2285
	Molecular	0.2584444	0.07613	0.0974459	0.51
C	All	-0.4917134	$3.628x10^{7}$	-0.4889275	$4.318x10^{7}$
	Morphological	-0.5086812	0.0002228	-0.537109	$8.285x10^{5}$
	Molecular	-0.4637834	0.0008999	-0.4411145	0.0017
Res	All	-0.08618136	0.4038	0.01685446	0.8705
	Morphological	-0.09755169	0.5095	-0.0280663	0.8498
	Molecular	-0.103741	0.4829	0.1149934	0.4364
CI	All	-0.7468723	$2.2x10^{16}$	-0.6574519	$3.437x10^{13}$
	Morphological	-0.75699	$4.805x10^{10}$	-0.7043572	$2.318x10^{8}$
	Molecular	-0.7299358	$3.942x10^{9}$	-0.6269879	$1.859x10^{6}$
RI	All	-0.06550757	0.526	-0.0006098	0.9953
	Morphological	0.04190105	0.7774	0.135983	0.3568
	Molecular	-0.1700857	0.2478	-0.1262137	0.3927
CI & RI p-value	All	-0.3278507	0.001111	-0.217427	0.03334
	Morphological	-0.3812062	0.007513	-0.2961744	0.04096
	Molecular	-0.2783912	0.05537	-0.1359442	0.3569
bHER	All	0.0003667186	0.9972	-0.057820	0.5758
	Morphological	0.1244329	0.3994	0.01440711	0.9226
	Molecular	-0.1048171	0.4783	-0.1223862	0.4073

Supplementary Table 16: Results of tests for correlation between number of terminal taxa and the following metrics: number of region characters (Regions), the ratio of regions to terminal taxa (Regions:Taxa), number of phylogenetic characters (Size), year in which the source paper for the tree was published (Year), Colless's index of tree balance (*C*), proportion of resolved nodes (Res), consistency index (CI), retention index (RI), probability of CI & RI values falling within the null distribution (CI & RI p-value) and the biogeographic homoplasy excess ratio (bHER). Statistically significant results are highlighted in green. N = 96 morphological and molecular phylogenies.

Metric	Data partition	Spearman's rho (R_S)	P-value	Pearson's *r*	P-value
Taxa	All	-0.1505231	0.3072	-0.1690282	0.2508
Regions	All	0.6335398	$1.344x10^{6}$	0.7634851	$2.783x10^{10}$
Size	All	0.05913363	0.5713	-0.0773227	0.4588
	Morphological	0.157687	0.2953	-0.02597051	0.864
	Molecular	0.01156163	0.9378	-0.1111952	0.4518
Year	All	0.07449566	0.4707	0.1167169	0.2574
	Morphological	0.08586612	0.5617	0.138731	0.347
	Molecular	0.06587597	0.6564	0.09492784	0.521
C	All	0.08971911	0.3847	0.1575838	0.1252
	Morphological	0.1814135	0.2172	0.1947561	0.1847
	Molecular	-0.02730357	0.8538	0.1121406	0.4479
Res	All	0.06687311	0.5174	0.1704276	0.0969
	Morphological	0.1000948	0.4985	0.2143518	0.1435
	Molecular	0.06310125	0.67	0.1363707	0.3554
CI	All	0.02499941	0.809	-0.0524897	0.6115
	Morphological	0.04253067	0.7741	-0.03870252	0.794
	Molecular	-0.00162844	0.9912	-0.06436984	0.6638
RI	All	-0.05671988	0.5831	-0.1263729	0.2199
	Morphological	0.0740228	0.6171	-0.09797914	0.5076
	Molecular	-0.1791336	0.2231	-0.153801	0.2966
CI & RI p-value	All	0.07712512	0.4551	0.000731188	0.9944
	Morphological	0.01522884	0.9182	0.09328853	0.5283
	Molecular	0.1442504	0.328	-0.09867838	0.5046
bHER	All	-0.00470802	0.9637	-0.05516373	0.5935
	Morphological	0.03886446	0.7931	-0.00835293	0.9551
	Molecular	-0.06502741	0.6606	-0.0976651	0.509

Supplementary Table 17: Results of tests for correlation between the ratio of regions to terminal taxa and the following metrics: number of taxa (Taxa), number of region characters (Regions), number of phylogenetic characters (Size), year in which the source paper for the tree was published (Year), Colless's index of tree balance (*C*), proportion of resolved nodes (Res), consistency index (CI), retention index (RI), probability of CI & RI values falling within the null distribution (CI & RI p-value) and the biogeographic homoplasy excess ratio (bHER). Statistically significant results are highlighted in green. N = 96 morphological and molecular phylogenies.

Metric	Data partition	Spearman's rho (R_S)	P-value	Spearman's rho (R_S), no outliers	P-value	Pearson's *r*, no outliers	P-value
Taxa	All	-0.0730599	0.4841	-0.05696931	0.5917	-0.1417601	0.1801
	Morphological	-0.2022539	0.1777	-0.2022539	0.1777	-0.09719947	0.5205
	Molecular	-0.1629039	0.2686	-0.1336808	0.3813	-0.2190511	0.1483
Regions	All	0.005688736	0.9566	0.04249402	0.6892	-0.04527466	0.67
	Morphological	-0.06529939	0.6664	-0.06529939	0.6664	-0.05821717	0.7008
	Molecular	-0.06463897	0.6625	0.003365008	0.9825	-0.07076057	0.6442
Regions: Taxa	All	0.0970932	0.3519	0.1278146	0.2273	0.02876586	0.7866
	Morphological	0.1983655	0.1863	0.1983655	0.1863	-0.02584736	0.8646
	Molecular	0.07485209	0.6131	0.1401278	0.3586	0.04497281	0.7693
Size	All	0.948978	$< 2.2x10^{-16}$	0.9437631	$< 2.2x10^{-16}$	0.9014477	$< 2.2x10^{-16}$
	Morphological	0.8018752	$2.124x10^{-11}$	0.8018752	$2.124\ x10^{-11}$	0.9959297	$< 2.2x10^{-16}$
	Molecular	0.8739687	$< 2.2x10^{-16}$	0.8470356	$< 2.2x10^{-16}$	0.8781236	$2.337x10^{-15}$
Year	All	0.277526	0.006766	0.2191488	0.03688	0.2765894	0.007954
	Morphological	0.2015343	0.1792	0.2015343	0.1792	0.2314577	0.1217
	Molecular	0.5489094	$5.35x10^{-05}$	0.4719041	0.001066	0.417605	0.004314
C	All	0.09326966	0.3712	0.08620642	0.4165	0.03454118	0.7451
	Morphological	0.3869117	0.007901	0.3869117	0.007901	-0.02838833	0.8514
	Molecular	0.09650718	0.5141	0.07193676	0.6376	0.1224647	0.4229
Res	All	0.402723	$4.745\ x\ 10^{-5}$	0.3651052	0.0005474	0.2385515	0.02697
	Morphological	0.4760757	0.0006257	0.3831965	0.0112	0.3579267	0.01844
	Molecular	0.1253834	0.3958	0.02452057	0.876	0.1110335	0.4784
CI	All	-0.02422169	0.8167	0.01598659	0.8804	0.1243427	0.2403
	Morphological	0.03139553	0.8359	0.03139553	0.8359	-0.1136319	0.4521
	Molecular	0.008847396	0.9524	0.1046807	0.4938	0.1577679	0.3006
RI	All	0.1748563	0.09187	0.1485686	0.1599	0.2263685	0.03095
	Morphological	0.1067826	0.48	0.1067826	0.48	0.04290317	0.7771
	Molecular	0.1802095	0.2203	0.1367679	0.3703	0.2837668	0.05889
CI & RI p-value	All	-0.09990568	0.338	-0.1229968	0.2454	-0.1345534	0.2035
	Morphological	-0.04090194	0.7872	-0.04090194	0.7872	0.07473912	0.6216
	Molecular	-0.04167008	0.7785	-0.0813191	0.5954	-0.1560513	0.306
bHER	All	0.2395648	0.02004	0.2445721	0.01947	0.2893585	0.005404
	Morphological	0.2265396	0.13	0.2265396	0.13	-0.03663787	0.809
	Molecular	0.165762	0.2593	0.1860343	0.2204	0.331806	0.02597

Supplementary Table 18: Results of tests for correlation between the ratio of phylogenetic characters to terminal taxa and the following metrics: number of taxa (Taxa), number of region characters (Regions), the ratio of regions to terminal taxa (Regions:Taxa), number of phylogenetic characters (Size), year in which the source paper for the tree was published (Year), Colless's index of tree balance (*C*), proportion of resolved nodes (Res), consistency index (CI), retention index (RI), probability of CI & RI values falling within the null distribution (CI & RI p-value) and the biogeographic homoplasy excess ratio (bHER). Spearman's rank-order correlations were calculated for the whole dataset, as well as for a subset of the data in which outlying high values (> 9,000 characters) were removed before calculating correlation coefficients. Pearson correlation coefficients were only calculated for the dataset with outliers removed. Statistically significant results are highlighted in green. N = 96 morphological and molecular phylogenies (all data), n = 90 morphological and molecular phylogenies (no outliers).

Supplementary References

1 IUCN. *The IUCN Red List of Threatened Species. Version 2019-2.*, <https://www.iucnredlist.org> (2019).

2 GBIF.org. *GBIF Home Page*, <https://www.gbif.org/> (2019).

3 Uetz, P., Freed, P., Aguilar, R. & Hošek, J. *The reptile database.*, <http://www.reptiledatabase.org> (2019).

4 Holt, B. G. *et al.* An update of Wallace's zoogeographic regions of the world. *Science* **339**, 74-78 (2013).

5 Colless, D. H. Review of phylogenetics: the theory and practice of phylogenetic systematics. *Systematic Zoology* **31**, 100-104 (1982).

6 O'Connor, A. & Wills, M. A. Measuring stratigraphic congruence across trees, higher taxa, and time. *Systematic Biology* **65**, 792-811 (2016).

7 Swofford, D. PAUP*: Phylogenetic Analysis Using Parsimony, Version 4.0a.154. (2017).

8 Hollander, M., Wolfe, D. A. & Chicken, E. *Nonparametric statistical methods*. Vol. 751 (John Wiley & Sons, 2013).

9 Kluge, A. G. & Farris, J. S. Quantitative phyletics and evolution of anurans. *Systematic Zoology* **18**, 1-32 (1969).

10 Sanderson, M. J. & Donoghue, M. J. Patterns of variation in levels of homoplasy. *Evolution* **43**, 1781-1795 (1989).

11 Archie, J. W. in *Homoplasy: The recurrence of similarity in evolution* (eds M. J. Sanderson & L. Hufford) 153-188 (Academic Press, 1996).

12 Farris, J. S. The retention index and the rescaled consistency index. *Cladistics* **5**, 417-419 (1989).

13 Archie, J. W. Homoplasy excess ratios: new indices for measuring levels of homoplasy in phylogenetic systematics and a critique of the consistency index. *Systematic Zoology* **38**, 253-269 (1989).

14 Naylor, G. & Kraus, F. The relationship between *s* and *m* and the retention index. *Systematic Biology* **44**, 559-562 (1995).

15 Farris, J. S. The retention index and homoplasy excess. *Systematic Zoology* **38**, 406-407 (1989).

16 Archie, J. W. Homoplasy excess statistics and retention indices: a reply to Farris. *Systematic Zoology* **39**, 169-174 (1990).

17 Wilkinson, M. *PICA 4.0: software and documentation.*, (Department of Zoology, The Natural History Museum, London, 2001).

18 R Core Team. *R: a language and environment for statistical computing.*, <https://www.R-project.org/> (2021).

19 Alroy, J. *Fossilworks: Gateway to the Paleobiology Database*, <http://fossilworks.org> (2019).

20 Benton, M. J. *The Fossil Record 2*. (Chapman & Hall, 1993).

21 Cohen, K. M., Harper, D. A. T. & Gibbard, P. L. *ICS International Chronostratigraphic Chart 2021/02*, <http://www.stratigraphy.org/> (2021).

22 Gradstein, F. & Ogg, J. Geologic time scale 2004–why, how, and where next! *Lethaia* **37**, 175-181 (2004).

23 Rohde, R. A. *The GeoWhen Database*, 2005).

24 Bell, M. A. & Lloyd, G. T. strap: an R package for plotting phylogenies against stratigraphy and assessing their stratigraphic congruence. Report No. 0031-0239, (Wiley Online Library, 2015).

25 Wills, M. A., Barrett, P. M. & Heathcote, J. F. The modified gap excess ratio (GER*) and the stratigraphic congruence of dinosaur phylogenies. *Systematic Biology* **57**, 891-904 (2008).

26 Huelsenbeck, J. P. Comparing the stratigraphic record to estimates of phylogeny. *Paleobiology* **20**, 470-483 (1994).

27 O'Connor, A., Moncrieff, C. & Wills, M. A. Variation in stratigraphic congruence (GER) through the Phanerozoic and across higher taxa is partially determined by sources of bias. *Geological Society of London Special Publication* **358**, 31-52 (2011).

28 Siddall, M. E. Stratigraphic fit to phylogenies: a proposed solution. *Cladistics* **14**, 201-208 (1998).

29 Wills, M. A. Congruence between phylogeny and stratigraphy: randomization tests and the gap excess ratio. *Systematic Biology* **48**, 559-580 (1999).

30 Clopper, C. J. & Pearson, E. S. The use of confidence or fiducial limits illustrated in the case of the binomial. *Biometrika* **26**, 404-413 (1934).

31 Omland, K. E. Character Congruence Between a Molecular and a Morphological Phylogeny for Dabbling Ducks (*Anas*). *Systematic Biology* **43**, 369-386 (1994).

32 Pennington, R. T. Molecular and morphological data provide phylogenetic resolution at different hierarchical levels in *Andira*. *Systematic Biology* **45**, 496-515 (1996).

33 Jackman, T. R., Larson, A., de Queiroz, K. & Losos, J. B. Phylogenetic relationships and tempo of early diversification in *Anolis* lizards. *Systematic Biology* **48**, 254-285 (1999).

34 Finarelli, J. A. A total evidence phylogeny of the Arctoidea (Carnivora: Mammalia): relationships among basal taxa. *Journal of Mammalian Evolution* **15**, 231-259 (2008).

35 Flynn, J. J., Finarelli, J. A., Zehr, S., Hsu, J. & Nedbal, M. A. Molecular phylogeny of the Carnivora (Mammalia): assessing the impact of increased sampling on resolving enigmatic relationships. *Systematic Biology* **54**, 317-337 (2005).

36 Fenwick, A. M., Gutberlet, R. L., Evans, J. A. & Parkinson, C. L. Morphological and molecular evidence for phylogeny and classification of South American pitvipers, genera *Bothrops*, *Bothriopsis*, and *Bothrocophias* (Serpentes: Viperidae). *Zoological Journal of the Linnean Society* **156**, 617-640 (2009).

37 Zrzavý, J. & Řlčánková, V. Phylogeny of recent Canidae (Mammalia, Carnivora): relative reliability and utility of morphological and molecular datasets. *Zoologica Scripta* **33**, 311-333 (2004).

38 Kay, R. F. The phyletic relationships of extant and fossil Pitheciinae (Platyrrhini, Anthropoidea). *The Platyrrhine Fossil Record*, 175-208 (1990).

39 Schneider, H. *et al.* Molecular phylogeny of the New World monkeys (Platyrrhini, primates). *Molecular Phylogenetics and Evolution* **2**, 225-242 (1993).

40 Simmons, N. B., Seymour, K. L., Habersetzer, J. & Gunnell, G. F. Primitive Early Eocene bat from Wyoming and the evolution of flight and echolocation. *Nature* **451**, 818-821 (2008).

41 Teeling, E. C. *et al.* A molecular phylogeny for bats illuminates biogeography and the fossil record. *Science* **307**, 580-584 (2005).

42 Fracasso, M. P. A., de Oliveira Salles, L. & Perini, F. A. Upper molar morphology and relationships among higher taxa in bats. *Journal of mammalogy* **92**, 421-432 (2011).

43 Agnarsson, I., Zambrana-Torrelio, C. M., Flores-Saldana, N. P. & May-Collado, L. J. A time-calibrated species-level phylogeny of bats (Chiroptera, Mammalia). *PLoS currents* **3**, doi:10.1371%2Fcurrents.RRN1212 (2011).

44 Asher, R. J. *et al.* .A phylogenetic estimate for golden moles (Mammalia, Afrotheria, Chrysochloridae). *BMC evolutionary biology* **10**, 1-13 (2010).

45 Gatesy, J., Baker, R. H. & Hayashi, C. Inconsistencies in arguments for the supertree approach: supermatrices versus supertrees of Crocodylia. *Systematic Biology* **53**, 342-355 (2004).

46 Oaks, J. R. A time-calibrated species tree of Crocodylia reveals a recent radiation of the true crocodiles. *Evolution* **65**, 3285-3297 (2011).

47 Gadek, P. A., Alpers, D. L., Heslewood, M. M. & Quinn, C. J. Relationships within Cupressaceae sensu lato: a combined morphological and molecular approach. *American Journal of Botany* **87**, 1044-1057 (2000).

48 Jansa, S. A. & Voss, R. S. Phylogenetic relationships of the marsupial genus *Hyladelphys* based on nuclear gene sequences and morphology. *Journal of Mammalogy* **86**, 853-865 (2005).

49 Oliveira, E. V., Nova, P. V., Goin, F. J. & Avilla, L. D. A new hyladelphine marsupial (Didelphimorphia, Didelphidae) from cave deposits of northern Brazil. *Zootaxa* **3041**, 51-62 (2011).

50 Voss, R. S. & Jansa, S. A. Phylogenetic relationships and classification of didelphid marsupials, an extant radiation of New World metatherian mammals. *Bulletin of the American Museum of Natural History* **2009**, 1-177 (2009).

51 Horovitz, I. & Sánchez-Villagra, M. R. A morphological analysis of marsupial mammal higher-level phylogenetic relationships. *Cladistics* **19**, 181-212 (2003).

52 Meredith, R. W., Westerman, M. & Springer, M. S. A phylogeny of Diprotodontia (Marsupialia) based on sequences for five nuclear genes. *Molecular Phylogenetics and Evolution* **51**, 554-571 (2009).

53 Piano, F., Craddock, E. M. & Kambysellis, M. P. Phylogeny of the island populations of the Hawaiian *Drosophila grimshawi* complex: evidence from combined data. *Molecular Phylogenetics and Evolution* **7**, 173-184 (1997).

54 Olivares, A. I. & Verzi, D. H. Systematics, phylogeny and evolutionary pattern of the hystricognath rodent *Eumysops* (Echimyidae) from the Plio-Pleistocene of southern South America. *Historical Biology* **27**, 1042-1061 (2015).

55 Kluge, A. G. A concern for evidence and a phylogenetic hypothesis of relationships among *Epicrates* (Boidae, Serpentes). *Systematic Biology* **38**, 7-25 (1989).

56 Tolson, P. J. *Phylogenetics of the boid snake genus Epicrates and Caribbean vicariance theory.*, University of Michigan, (1987).

57 He, K. *et al.* An estimation of Erinaceidae phylogeny: a combined analysis approach. *PLoS One* **7**, e39304, doi:10.1371/journal.pone.0039304 (2012).

58 O'Leary, M. A. *et al.* The placental mammal ancestor and the post–K-Pg radiation of placentals. *Science* **339**, 662-667 (2013).

59 Gaubert, P., Wozencraft, W. C., Cordeiro-Estrela, P. & Veron, G. Mosaics of convergences and noise in morphological phylogenies: what's in a viverrid-like carnivoran? *Systematic Biology* **54**, 865-894 (2005).

60 Asher, R. J. *et al.* Stem lagomorpha and the antiquity of Glires. *Science* **307**, 1091-1094 (2005).

61 Brown Jr, K. S. The biology of *Heliconius* and related genera. *Annual review of entomology* **26**, 427-457 (1981).

62 Brower, A. V. Z. Phylogeny of *Heliconius* butterflies inferred from mitochondrial DNA sequences (Lepidoptera: Nymphalidae). *Molecular Phylogenetics and Evolution* **3**, 159-174 (1994).

63 Schulte, J. A., Valladares, J. P. & Larson, A. Phylogenetic relationships within Iguanidae inferred using molecular and morphological data and a phylogenetic taxonomy of iguanian lizards. *Herpetologica* **59**, 399-419 (2003).

64 Sites Jr, J. W., Davis, S. K., Guerra, T., Iverson, J. B. & Snell, H. L. Character congruence and phylogenetic signal in molecular and morphological data sets: a case study in the living iguanas (Squamata, Iguanidae). *Molecular Biology and Evolution* **13**, 1087-1105 (1996).

65 Miller, J. A. Phylogeny of the Neotropical moth tribe Josiini (Notodontidae: Dioptinae): a hidden case of Müllerian mimicry. *Zoological Journal of the Linnean Society* **118**, 1-45 (1996).

66 Miller, J. S., Brower, A. V. & Desalle, R. Phylogeny of the neotropical moth tribe Josiini (Notodontidae: Dioptinae): comparing and combining evidence from DNA sequences and morphology. *Biological Journal of the Linnean Society* **60**, 297-316 (1997).

67 Kim, K. J. & Jansen, R. K. Comparisons of phylogenetic hypotheses among different data sets in dwarf dandelions (*Krigia*, Asteraceae): additional information from internal transcribed spacer sequences of nuclear ribosomal DNA. *Plant Systematics and Evolution* **190**, 157-185 (1994).

68 Prideaux, G. J. & Warburton, N. M. An osteology-based appraisal of the phylogeny and evolution of kangaroos and wallabies (Macropodidae: Marsupialia). *Zoological Journal of the Linnean Society* **159**, 954-987 (2010).

69 Mitchell, K. J. *et al.* Molecular phylogeny, biogeography, and habitat preference evolution of marsupials. *Molecular Biology and Evolution* **31**, 2322-2330 (2014).

70 Giannini, N. P. & Simmons, N. B. Conflict and congruence in a combined DNA–morphology analysis of megachiropteran bat relationships (Mammalia: Chiroptera: Pteropodidae). *Cladistics* **21**, 411-437 (2005).

71 Simmons, N. B. & Conway, T. M. Phylogenetic relationships of mormoopid bats (Chiroptera: Mormoopidae) based on morphological data. *Bulletin of the American Museum of Natural History* **2001**, 1-101 (2001).

72 Lewis-Oritt, N., Porter, C. A. & Baker, R. J. Molecular systematics of the family Mormoopidae (Chiroptera) based on cytochrome b and recombination activating gene 2 sequences. *Molecular Phylogenetics and Evolution* **20**, 426-436 (2001).

73 Sotiaux, A., Enroth, J., Olsson, S., Quandt, D. & Vanderpoorten, A. When morphology and molecules tell us different stories: a case-in-point with *Leptodon corsicus*, a new and unique endemic moss species from Corsica. *Journal of Bryology* **31**, 186-196 (2009).

74 Futuyma, D. J. & McCafferty, S. S. Phylogeny and the evolution of host plant associations in the leaf beetle genus *Ophraella* (Coleoptera, Chrysomelidae). *Evolution* **44**, 1885-1913 (1990).

75 Funk, D. J., Futuyma, D. J., Ortí, G. & Meyer, A. Mitochondrial DNA sequences and multiple data sets: a phylogenetic study of phytophagous beetles (Chrysomelidae: Ophraella). *Molecular Biology and Evolution* **12**, 627-640 (1995).

76 Titus, T. A. & Frost, D. R. Molecular homology assessment and phylogeny in the lizard family Opluridae (Squamata: Iguania). *Molecular Phylogenetics and Evolution* **6**, 49-62 (1996).

77 Dávalos, L. M., Cirranello, A. L., Geisler, J. H. & Simmons, N. B. Understanding phylogenetic incongruence: lessons from phyllostomid bats. *Biological Reviews* **87**, 991-1024 (2012).

78 Carstens, B. C., Lundrigan, B. L. & Myers, P. A phylogeny of the neotropical nectar-feeding bats (Chiroptera: Phyllostomidae) based on morphological and molecular data. *Journal of Mammalian Evolution* **9**, 23-53 (2002).

79 Reeder, T. W. & Wiens, J. J. Evolution of the lizard family Phrynosomatidae as inferred from diverse types of data. *Herpetological Monographs* **10**, 43-84 (1996).

80 Cannatella, D. C. *et al.* Phylogeny of frogs of the *Physalaemus pustulosus* species group, with an examination of data incongruence. *Systematic Biology* **47**, 311-335 (1998).

81 Klymiuk, A. A. & Stockey, R. A. A Lower Cretaceous (Valanginian) seed cone provides the earliest fossil record for *Picea* (Pinaceae). *American Journal of Botany* **99**, 1069-1082 (2012).

82 Wang, X.-Q., Tank, D. C. & Sang, T. Phylogeny and divergence times in Pinaceae: evidence from three genomes. *Molecular Biology and Evolution* **17**, 773-781 (2000).

83 Hart, J. A. A cladistic analysis of conifers: preliminary results. *Journal of the Arnold Arboretum* **68**, 269-307 (1987).

84 Tsumura, Y., Yoshimura, K., Tomaru, N. & Ohba, K. Molecular phytogeny of conifers using RFLP analysis of PCR-amplified specific chloroplast genes. *Theoretical and Applied Genetics* **91**, 1222-1236 (1995).

85 Liebherr, J. K. & Zimmerman, E. C. Cladistic analysis, phylogeny and biogeography of the Hawaiian Platynini (Coleoptera: Carabidae). *Systematic Entomology* **23**, 137-172 (1998).

86 Cryan, J. R., Liebherr, J. K., Fetzner Jr, J. W. & Whiting, M. F. Evaluation of relationships within the endemic Hawaiian Platynini (Coleoptera: Carabidae) based on molecular and morphological evidence. *Molecular Phylogenetics and Evolution* **21**, 72-85 (2001).

87 Bogdanowicz, W., Kasper, S. & Owen, R. D. Phylogeny of plecotine bats: reevaluation of morphological and chromosomal data. *Journal of Mammalogy* **79**, 78-90 (1998).

88 Hoofer, S. R. & Van Den Bussche, R. A. Phylogenetic relationships of plecotine bats and allies based on mitochondrial ribosomal sequences. *Journal of Mammalogy* **82**, 131-137 (2001).

89 Worthy, T. H. & Scofield, R. P. Twenty-first century advances in knowledge of the biology of moa (Aves: Dinornithiformes): a new morphological analysis and moa diagnoses revised. *New Zealand Journal of Zoology* **39**, 87-153 (2012).

90 Mitchell, K. J. *et al.* Ancient DNA reveals elephant birds and kiwi are sister taxa and clarifies ratite bird evolution. *Science* **344**, 898-900 (2014).

91 Wahlberg, N. *et al.* Synergistic effects of combining morphological and molecular data in resolving the phylogeny of butterflies and skippers. *Proceedings of the Royal Society B: Biological Sciences* **272**, 1577-1586 (2005).

92 Cardini, A. The geometry of the marmot (Rodentia: Sciuridae) mandible: phylogeny and patterns of morphological evolution. *Systematic Biology* **52**, 186-205 (2003).

93 Steppan, S. J. *et al.* Molecular phylogeny of the marmots (Rodentia: Sciuridae): tests of evolutionary and biogeographic hypotheses. *Systematic Biology* **48**, 715-734 (1999).

94 Bertelli, S. & Giannini, N. P. A phylogeny of extant penguins (Aves: Sphenisciformes) combining morphology and mitochondrial sequences. *Cladistics* **21**, 209-239 (2005).

95 Potter, D. & Doyle, J. J. Phylogeny and systematics of *Sphenostylis* and *Nesphostylis* (Leguminosae: Phaseoleae) based on morphological and chloroplast DNA data. *Systematic Botany* **19**, 389-406 (1994).

96 Estes, R., de Queiroz, K. & Gauthier, J. in *Phylogenetic Relationships of the Lizard Families* 119-281 (1988).

97 Wiens, J. J. *et al.* Resolving the phylogeny of lizards and snakes (Squamata) with extensive sampling of genes and species. *Biology Letters* **8**, 1043-1046 (2012).

98 Sánchez-Villagra, M. R., Horovitz, I. & Motokawa, M. A comprehensive morphological analysis of talpid moles (Mammalia) phylogenetic relationships. *Cladistics* **22**, 59-88 (2006).

99 Shinohara, A. *et al.* Evolution and biogeography of talpid moles from continental East Asia and the Japanese islands Inferred from mitochondrial and nuclear gene sequences. *Zoological Science* **21**, 1177-1185 (2004).
