# Supplementary material for: Molecular phylogenies map to biogeography better than morphological ones
Source: Commun Biol. 2022 May 31;5:521. doi: 10.1038/s42003-022-03482-x (PMC9156683; doi:10.1038/s42003-022-03482-x)
Supplement: Supplementary file 3 — Reporting Summary [file 42003_2022_3482_MOESM3_ESM.pdf]

nature portfolio

Corresponding author(s): Jack W. Oyston
Matthew A. Wills

Last updated by author(s): Apr 24, 2022

Reporting Summary

Nature Portfolio wishes to improve the reproducibility of the work that we publish. This form provides structure for consistency and transparency in reporting. For further information on Nature Portfolio policies, see our Editorial Policies and the Editorial Policy Checklist.

Statistics

For all statistical analyses, confirm that the following items are present in the figure legend, table legend, main text, or Methods section.

n/a	Confirmed	
☐	☒	The exact sample size (*n*) for each experimental group/condition, given as a discrete number and unit of measurement
☐	☒	A statement on whether measurements were taken from distinct samples or whether the same sample was measured repeatedly
☐	☒	The statistical test(s) used AND whether they are one- or two-sided<br>*Only common tests should be described solely by name; describe more complex techniques in the Methods section.*
☐	☒	A description of all covariates tested
☐	☒	A description of any assumptions or corrections, such as tests of normality and adjustment for multiple comparisons
☐	☒	A full description of the statistical parameters including central tendency (e.g. means) or other basic estimates (e.g. regression coefficient) AND variation (e.g. standard deviation) or associated estimates of uncertainty (e.g. confidence intervals)
☐	☒	For null hypothesis testing, the test statistic (e.g. *F*, *t*, *r*) with confidence intervals, effect sizes, degrees of freedom and *P* value noted<br>*Give P values as exact values whenever suitable.*
☒	☐	For Bayesian analysis, information on the choice of priors and Markov chain Monte Carlo settings
☐	☒	For hierarchical and complex designs, identification of the appropriate level for tests and full reporting of outcomes
☐	☒	Estimates of effect sizes (e.g. Cohen's *d*, Pearson's *r*), indicating how they were calculated

Our web collection on statistics for biologists contains articles on many of the points above.

Software and code

Policy information about availability of computer code

Data collection	No software was used.
Data analysis	Unless otherwise stated data analyses were conducted in R (https://www.r-project.org/). Optimization of biogeographical region matrices onto phylogenies and calculation of Consistency Index and Retention index values was performed in PAUP*: Phylogenetic Analysis Using Parsimony, Version 4.0a.154 (https://paup.phylosolutions.com). CI & RI randomisation tests and calculation of the Biogeographical Homoplasy Excess Ratio (bHER) was carried out in PAUP* 4.0a.154 using a custom BASIC script run though Chipmunk Basic v3.6.4 (http://www.nicholson.com/rhn/basic/). Independent matrix compatibility permutation tail probability (MCPTP) tests were carried out using the MATRIX program in PICA 4.0 (http://www.bmnh.org/PICA.html). Phylogenies were time-calibrated using the package 'Strap' in R and other indices of stratigraphic fit calculated using the GHOSTS 2.4 program.

For manuscripts utilizing custom algorithms or software that are central to the research but not yet described in published literature, software must be made available to editors and reviewers. We strongly encourage code deposition in a community repository (e.g. GitHub). See the Nature Portfolio guidelines for submitting code & software for further information.

Data

Policy information about availability of data

All manuscripts must include a data availability statement. This statement should provide the following information, where applicable:

- Accession codes, unique identifiers, or web links for publicly available datasets
- A description of any restrictions on data availability
- For clinical datasets or third party data, please ensure that the statement adheres to our policy

The data that support the findings of this study are available from the corresponding authors upon request and on figshare with the identifier https://
